# Template-Directed Selective Photodimerization Reactions of 5-Arylpenta-2,4-dienoic Acids

**DOI:** 10.1021/acs.joc.4c01374

**Published:** 2024-07-10

**Authors:** Badar Munir, Bilge Banu Yagci, Yunus Zorlu, Yunus E. Türkmen

**Affiliations:** † Department of Chemistry, Faculty of Science, Bilkent University, Ankara 06800, Türkiye; § Department of Chemistry, Gebze Technical University, Gebze, Kocaeli 41400, Türkiye; ‡ UNAM — National Nanotechnology Research Center, Institute of Materials Science and Nanotechnology, Bilkent University, Ankara 06800, Türkiye

## Abstract

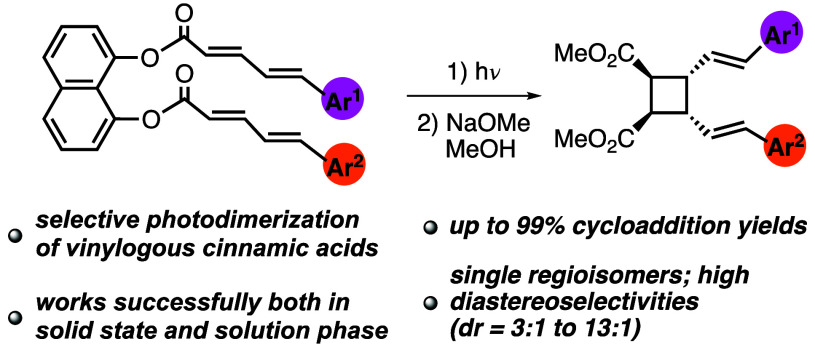

We developed an efficient method that enables selective photodimerization of 5-arylpenta-2,4-dienoic acids (i.e., vinylogous cinnamic acids). The use of 1,8-dihydroxynaphthalene as a template ensures proximity of the two reacting olefins so that irradiation of template-bound dienoic acids gives mono [2 + 2] cycloaddition products in good to excellent yields (up to 99%), as single regioisomers, and with high diastereoselectivities (dr = 3:1 to 13:1). The geometrical and stereochemical features of compounds **12a**, **16a**, and **22a** were analyzed by X-ray crystallography.

Photochemical [2 + 2] cycloadditions of olefins provide direct access to multisubstituted cyclobutanes.^1^ Although remarkable advances have been witnessed since the early studies of Ciamician,^2^ analogous reactions of dienes and higher polyenes are surprisingly underdeveloped despite their synthetic potential.^3^ In one pathway, photodimerization of diene **1** may provide [3]-ladderane product **2** via double [2 + 2] cycloaddition reactions (Scheme 1). [n]-Ladderanes^4^ are a class of structurally intriguing polycyclobutanes, which are also present in naturally occurring phospholipids in anammox bacteria.^5,6^ Pioneering work of Hopf and co-workers demonstrated that [2,2]-paracyclophane core could be used to suitably orient two polyenes for the synthesis of [3]- and [5]-ladderanes (Scheme 2a).^7^ In an elegant design reported in 2004, MacGillivray and co-workers used 5-methoxyresorcinol (**3**) as a hydrogen bonding template to build supramolecular assemblies with bis(4-pyridyl)polyenes **4**, the irradiation of which afforded quantitatively [3]- and [5]-ladderanes **5a** and **5b** (Scheme 2b).^8^

**Scheme 1:**
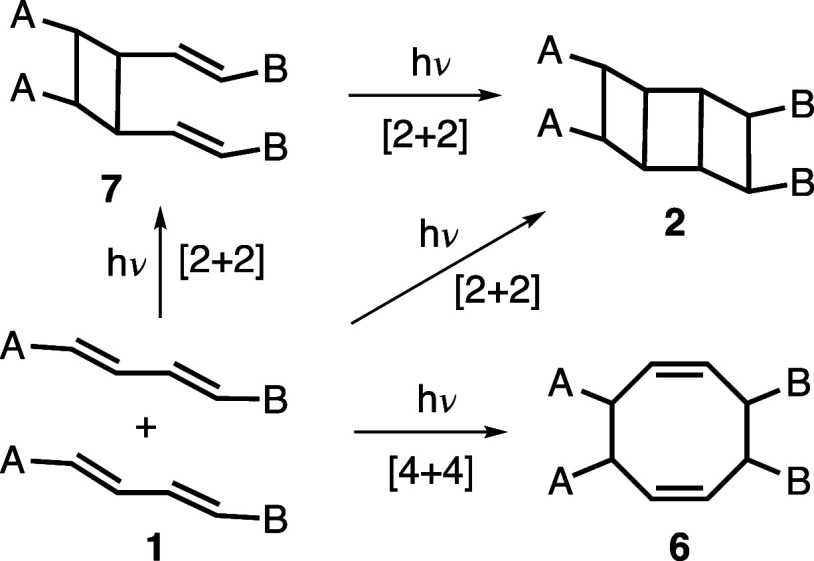
Possible Photodimerization Pathways of **1**

**Scheme 2:**
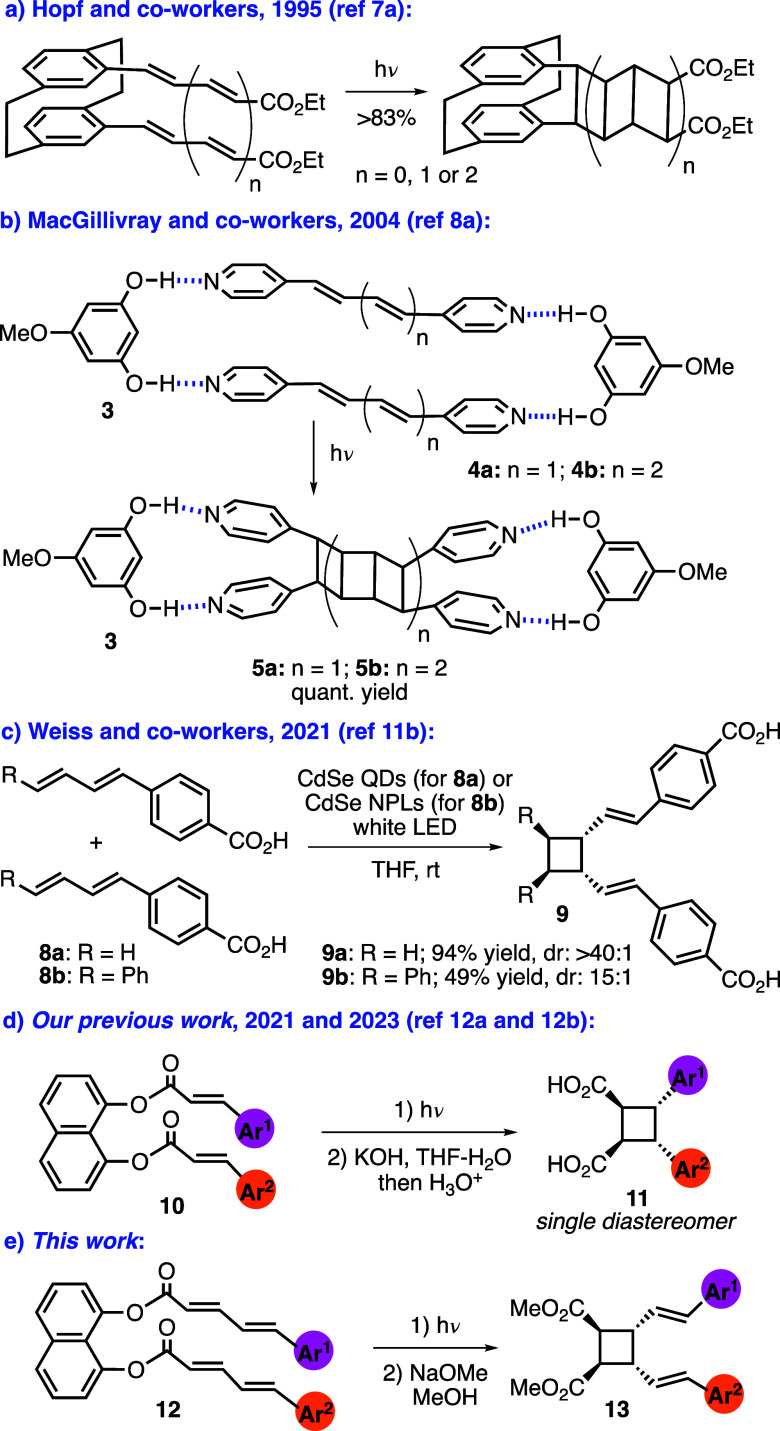
Examples of [2 + 2] Cycloadditions of Alkenes and Dienes

Alternatively, irradiation of **1** may give cyclooctadiene **6** either via a direct [4 + 4] cycloaddition or an initial [2 + 2] cycloaddition followed by a thermal Cope rearrangement of divinylcyclobutane **7** (Scheme 1).^9^ As a third possibility, due to certain electronic and/or geometrical factors, photodimerization of **1** may proceed via a single [2 + 2] cycloaddition affording divinylcyclobutane **7** (Scheme 1).^10^ In their elegant studies, Weiss and co-workers showed that intermolecular [2 + 2] cycloadditions can be photocatalyzed using quantum dots via triplet–triplet energy transfer.^11^ In particular, subjecting dienes **8** to photocatalysis by CdSe quantum dots or nanoplatelets gave divinylcyclobutanes **9** with high diastereoselectivities (Scheme 2c).^11b^

We recently developed a general solution for the selective photodimerization of cinnamic acids using 1,8-dihydroxynaphthalene (1,8-DHN) as a covalent template.^12^ In this design, irradiation of template-bound cinnamic acids **10** followed by hydrolysis gave symmetrical and unsymmetrical β-truxinic acids **11** in high yields and as single diastereomers (Scheme 2d). Despite the rich background^1b^ and recent advances^12,13^ in the photodimerization of cinnamic acid derivatives, the analogous reactions of vinylogous cinnamic acids, namely 5-arylpenta-2,4-dienoic acids, are scarce. Indeed, besides the work of Hopf mentioned above,^7^ and an early report from 1913,^14^ to our knowledge, there are only a few studies which involve photodimerization of 5-arylpenta-2,4-dienoic acids. In 1971, Schmidt and co-workers reported the formation of a complex mixture of four- and eight-membered products (seven spots on TLC other than the reactant) upon solid-state irradiation of 5-phenylpenta-2,4-dienoic acid.^9a^ In a second study, irradiation of 5-(3-methoxyphenyl)penta-2,4-dienoic acid in solid state was reported by Mascitti and Corey to proceed with a head-to-tail [2 + 2] cycloaddition between the two different olefins of the reactants.^6b^ Recently, Yoon and co-workers reported a single example of an *anti*-head-to-head [2 + 2] cycloaddition of a vinylogous cinnamamide derivative for the synthesis of nigramide R.^13b^ The lack of success in selective photodimerization of vinylogous cinnamic acids is not surprising given the challenges associated with the presence of two types of olefins that can react with one another, and due to the cycloaddition possibilities in *syn* and *anti* head-to-head and head-to-tail orientations resulting in 12 possible mono [2 + 2] cycloadducts without considering enantiomers (Figure S1). In this work, we applied our template-directed strategy to develop a general solution for the first time to the selective homo- and heterodimerization of 5-arylpenta-2,4-dienoic acids (Scheme 2e). This way, out of the 12 possible cycloaddition products, the *syn*-head-to-head cycloadducts **13** were obtained selectively.

The synthesis of [2 + 2] cycloaddition precursors is described in Scheme 3. The Horner-Wadsworth-Emmons reaction between cinnamaldehydes **14** and triethyl phosphonoacetate gave esters **15** with (*E*,*E*) configuration in 78–98% yields, and their subsequent hydrolysis afforded 5-arylpenta-2,4-dienoic acids **16** in uniformly high yields (89–98%). The synthesis of symmetrical diesters **12a**-**c** was accomplished via the reaction of 1,8-DHN (**17**) with excess dienoic acids using DCC (dicyclohexylcarbodiimide) in 66–72% yields. For the synthesis of unsymmetrical diesters **12d** and **21**, monoester **19** was prepared first by reacting 1,8-DHN (**17**) with acyl chloride **18** under basic conditions (71%). A subsequent coupling of **19** with dienoic acid **16d** afforded **12d** in moderate yield (48%). Finally, treatment of a 1:1 mixture of **19** and *trans*-cinnamoyl chloride (**20**) with one equivalent of NaH afforded diester **21** in 70% yield.

**Scheme 3:**
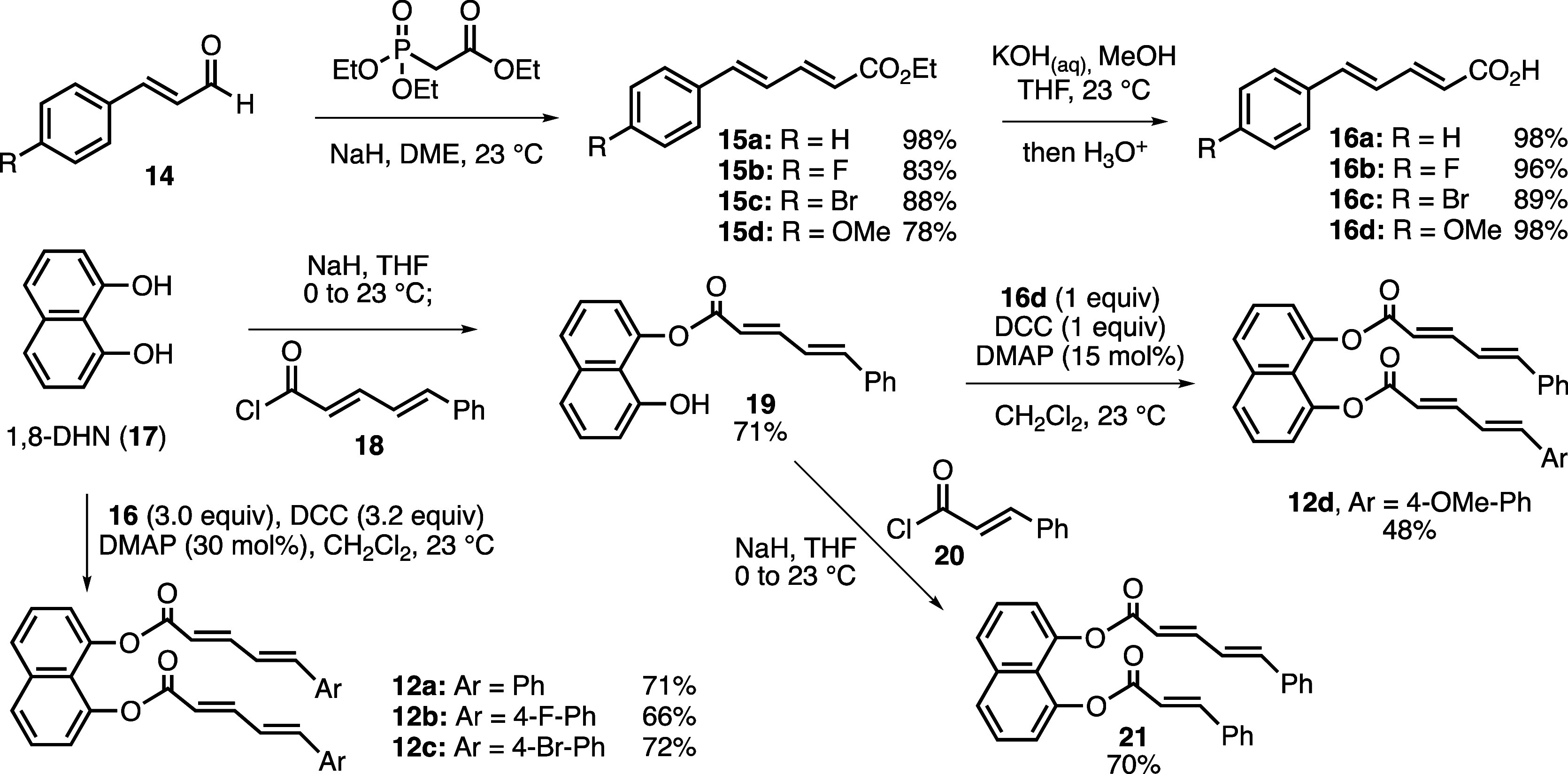
Synthesis of Template-Bound Cycloaddition Precursors

Our studies on the targeted photocycloaddition commenced by investigating the irradiation of diester **12a** (Tables S1 and S2). Initially, when a powder sample of **12a** was irradiated with UV light (365 nm) for 16 h, cycloadduct **22a** was isolated in 30% yield and with a diastereomeric ratio (dr) of 8:1 (Table S1, entry 1). Increasing the irradiation time to 48 h had a limited effect on the yield (52%, entry 2). The crystal structure of **12a** revealed that the two alkenes neighboring the carbonyl groups have criss-crossed geometry with a distance of 4.00 Å between their centroids (Figure 1a). However, *syn*-head-to-head photocycloaddition process was confirmed by the crystal structure of **22a** (Figure 1b). Pedal motion^15^ in solid state was previously proposed to account for the reactivity of such criss-crossed alkenes in [2 + 2] cycloadditions.^12a,16^ The distance of 4.00 Å between the reacting alkene centroids fullfills Schmidt criteria (<4.2 Å),^17^ and provides a rationale for the success of this reaction. Conversely, the distance between the second set of alkenes is 5.06 Å (Figure 1a). Even though this distance may be shorter after pedal motion, it is anticipated that it will still be greater than 4.2 Å rendering these olefins photoresistant.

**Figure 1:**
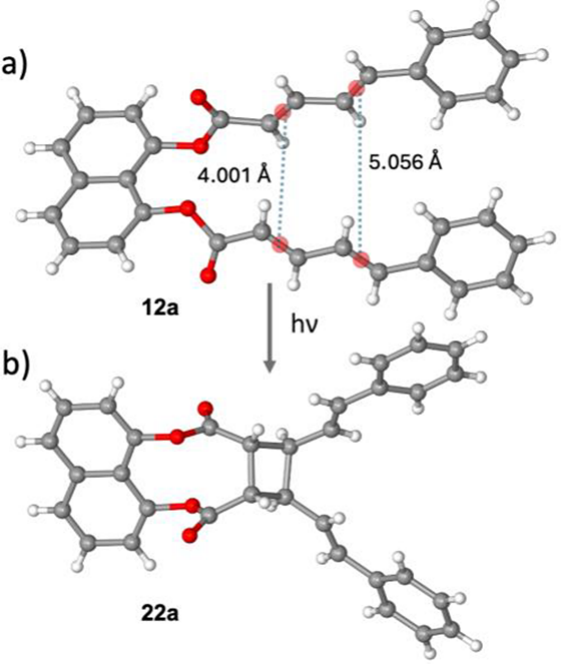
Crystal structures of **12a** and **22a**.

Since grinding was proposed to facilitate pedal motion in solid state,^16d^ we opted to check its effect on the reaction performance. However, when ground powder samples of **12a** were irradiated for 16 and 48 h, almost no improvement was observed (Table S1, entries 3 and 4). The powder XRD patterns of the powder and ground powder forms match the simulated pattern generated from its single-crystal XRD data meaning that structure of **12a** is retained in the bulk powder and ground powder forms (Figure S2). To our delight, running the cycloaddition in CHCl_3_ appeared to be superior providing **22a** in 88% yield (dr: 8:1, entry 5). When this reaction was performed on 1.0 mmol scale, **22a** was isolated in 61% yield (71% yield based on recovered starting material, entry 6). Finally, irradiation of a powder sample of untemplated **16a** gave a complex mixture of products, in agreement with Schmidt’s observation.^9a^ Our X-ray crystallographic analysis of **16a** shows a dimeric structure governed by hydrogen bonds, and matches the structure reported in 1980 (Figure S6).^18^ Its crystal packing reveals several olefin orientations which can potentially give [2 + 2] cycloadditions, providing an explanation for the formation of multiple products.

Next, we focused on the photochemical [2 + 2] reactions of other substrates (Scheme 4), which were tested both in solid state and solution (CHCl_3_), and subsequently, all cycloadducts were converted to their dimethyl esters via transesterification. For instance, cyclobutane dimethyl ester **13a** was obtained in 89% yield when **22a** was treated with NaOMe in MeOH. The [2 + 2] cycloaddition of 4-fluorophenyl-substituted diester **12b** proceeded with excellent yields in both solid state and solution (99 and 96%, respectively). However, reaction of the analogous 4-bromophenyl-substituted diester **12c** was inefficient in solid state affording cycloadduct **22c** in only 17% yield, possibly due to an unsuitable olefin orientation in crystal structure. Pleasingly, the same product was isolated in 63% yield and with high dr (11:1) when the reaction was performed in solution. Gratifyingly, reaction of the unsymmetrical diester **12d** proceeded successfully both in solid state and solution to provide the heterodimerization product **22d** in 53 and 84% yields, respectively. To our knowledge, this is the first example of a photochemical heterodimerization between two different 5-arylpenta-2,3-dienoic acids. Finally, the reaction of **21**, which possesses a dienoic acid and cinnamic acid units, gave [2 + 2] cycloadduct **23** in moderate yields of 47 and 42%, respectively, in solid state and solution. All cycloadducts were detached from the template by the aforementioned transesterification reaction affording cyclobutane dimethyl esters in 65–89% yields.

**Scheme 4:**
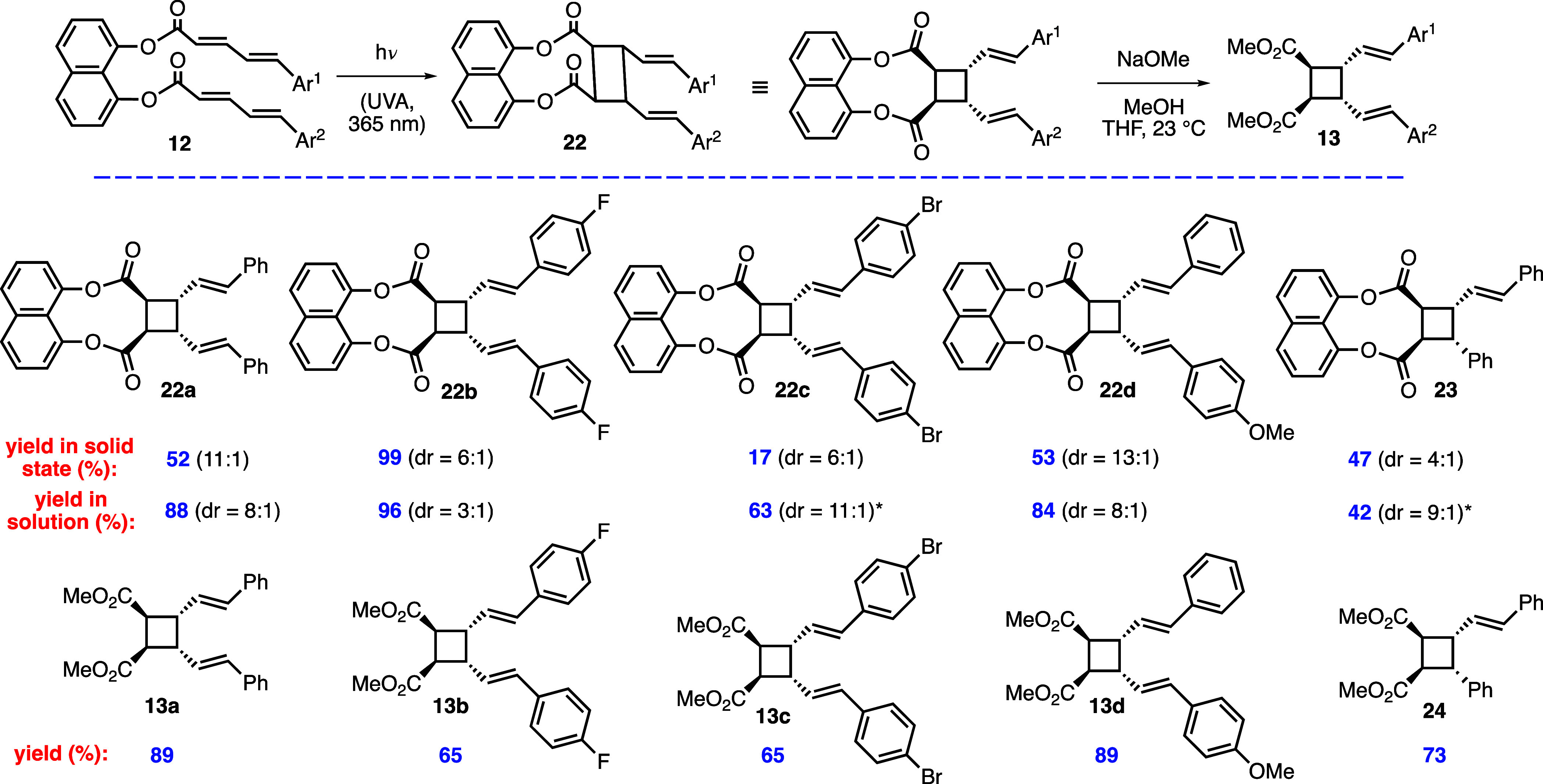
Synthesis of Cyclobutane Diesters **13** and **24** Diastereomeric ratio (dr) values were determined by ^1^H-NMR analysis of crude reaction mixtures. *These dr values belong to purified products.

In addition to the transesterification reactions, detachment from the template could also be achieved by other transformations. In this respect, hydrolysis of **22a** afforded dicarboxylic acid **25** in 69% yield (Scheme 5). Moreover, diol **26** was isolated in 65% yield upon reduction of the two ester groups of **22a**. In control experiments, when toluene solutions of diesters **12a** and **21** were heated at 100 °C under dark, no reaction was observed (Scheme S1). Finally, Cope rearrangement of **22a** and **13a** to access 1,5-cyclooctadiene products was attempted under thermal conditions (Tables S3 and S4). Disappointingly, none of the screened conditions gave the desired cyclooctadienes, possibly due to the steric clash of the two bulky phenyl rings in the transition states.

**Scheme 5:**
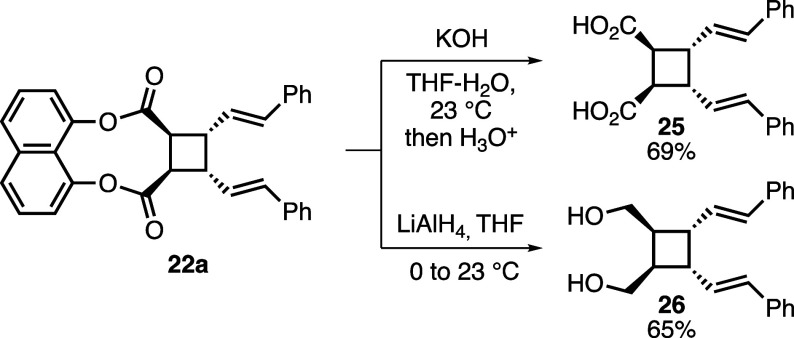
Hydrolysis and Reduction Reactions of **22a**

In conclusion, we developed the first general method for the selective photodimerization of vinylogous cinnamic acids. Attachment of two dienoic acids to 1,8-DHN (**17**) brings the two reactants spatially close to each other enabling an efficient [2 + 2] cycloaddition. Irradiation of diesters **12a**-**d** proceeded effectively both in solid state and solution affording the homo- and heterodimerization products **22a**-**d** in up to 99% yields, with full regiocontrol and high diastereoselectivities. Cyloadducts were demonstrated to be easily convertable to cyclobutane dicarboxylic ester, dicarboxylic acid and diol products. X-ray crystallographic analysis of diester **12a** provided a rationale for the observed regioselectivity, whereas the X-ray structure of cycloadduct **22a** confirmed its stereochemistry.

## Experimental Section

### General Information

All air or water sensitive reactions were performed using oven-dried glassware under nitrogen. Reactions were monitored by thin-layer chromatography (TLC) using aluminum-backed plates precoated with silica gel (Silicycle, 60 Å, F_254_). UV light (254 nm) and KMnO_4_ staining solution were used for TLC visualization. Flash column chromatography was carried out using Silicycle 40–63 μm (200–400 mesh) flash silica gel. NMR spectra were recorded using a Bruker spectrometer at 400 MHz for ^1^H NMR spectra and 100 MHz for ^13^C{^1^H} spectra, and calibrated from internal standard (TMS, 0 ppm) or residual solvent signals (chloroform at 7.26, DMSO at 2.50, and methanol at 3.31 ppm for ^1^H NMR spectra; chloroform at 77.16, DMSO at 39.52, and methanol at 49.00 ppm for ^13^C{^1^H}-NMR spectra). For ^19^F{^1^H}-NMR experiments, trifluoroacetic acid (CF_3_CO_2_H) was used as external reference (−76.55 ppm). ^1^H NMR data are reported as follows: chemical shift (ppm, parts per million), integration, multiplicity (s = singlet, d = doublet, t = triplet, dd = doublet of doublets, m = multiplet, br s = broad signal, app = apparent), coupling constant (Hz). Infrared (FTIR-ATR) spectra were recorded using a Bruker Alpha-Platinum-ATR spectrometer, and selected peaks are reported. HRMS (high resolution mass spectrometry) analyses were carried out at UNAM-National Nanotechnology Research Center and Institute of Materials Science and Nanotechnology, Bilkent University, using Agilent Technologies 6224 TOF LC/MS instrument. Single-crystal XRD analysis was performed at Gebze Technical University, Türkiye. Melting points are uncorrected. Photochemical reactions were performed using a commercial UV gel nail dryer (Elle by Beurer, MPE58) equipped with four 9W UV-A (365 nm) fluorescent lamps (Philips PL-S).^12^ Anhydrous CH_2_Cl_2_ and THF were purchased from Acros Organics (AcroSeal). 1,8-Dihydroxynaphthalene was purchased from abcr and used as received. All commercially available reagents were used without further purification, unless stated otherwise. **Caution!** Reactions which require oxalyl chloride, and its subsequent evaporation using a rotary evaporator should be conducted inside a well-ventilated fume hood. Also, caution should be taken when working with ultraviolet radiation.

### Ethyl (2*E*,4*E*)-5-Phenylpenta-2,4-dienoate (**15a**)

Triethylphosphonoacetate (2.21 g, 9.84 mmol) was dissolved in 11 mL of DME (1,2-dimethoxyethane) in an oven-dried 50 mL round-bottom flask under nitrogen at 23 °C. NaH (453 mg, 11.36 mmol, 60% dispersion in mineral oil) was added slowly to this solution cooled in an ice bath. Upon the addition of NaH, gas evolution was observed. The reaction mixture was allowed to stir for 25 min in ice bath. Then, *trans*-cinnamaldehyde (1.00 g, 7.57 mmol) was added to the reaction mixture, and the walls of the flask were rinsed with 2 mL of DME. The reaction mixture was stirred at 23 °C for 2 h, and the progress of the reaction was monitored using TLC (EtOAc/hexanes = 1:19). The reaction mixture was then quenched with a saturated aqueous solution of NH_4_Cl (15 mL). The aqueous phase was extracted thrice with CH_2_Cl_2_. The organic phases were combined, dried over anhydrous Na_2_SO_4_, filtered, and concentrated under vacuum. Purification by flash column chromatography (EtOAc/hexanes = 1:19) gave pure product **15a** (1.50 g, 98%) as a pale yellow oil. *R*_*f*_*=* 0.53 (EtOAc/hexanes = 1:19). ^1^H NMR (400 MHz, CDCl_3_) δ: 7.44–7.36 (3H, m), 7.30–7.21 (3H, m), 6.82–6.74 (2H, m), 5.96 (1H, d, *J* = 15.3 Hz), 4.21 (2H, q, *J* = 7.1 Hz), 1.28 (3H, t, *J* = 7.1 Hz). ^13^C{^1^H}-NMR (100 MHz, CDCl_3_) δ: 167.2, 144.6, 140.5, 136.2, 129.1, 128.9, 127.3, 126.4, 121.5, 60.5, 14.5. The NMR data are in agreement with the values reported in the literature.^19^

### Ethyl (2*E*,4*E*)-5-(4-Fluorophenyl)penta-2,4-dienoate (**15b**)

Compound **15b** was prepared from (*E*)-3-(4-fluorophenyl)acrylaldehyde (500 mg, 435 μL, 3.33 mmol), triethylphosphonoacetate (1.12 g, 5.00 mmol), NaH (200 mg, 5.00 mmol, 60% dispersion in mineral oil) and DME (5 mL) following the same procedure as used for compound **15a**. The crude product was purified using flash column chromatography (EtOAc/hexanes = 1:19) to afford **15b** (612 mg, 83%) as a white solid. *R*_*f*_*=* 0.42 (EtOAc/hexanes = 1:19) ^1^H NMR (400 MHz, CDCl_3_) δ: 7.41–7.35 (3H, m), 6.99 (2H, t, *J* = 8.6 Hz), 6.81–6.69 (2H, m), 5.94 (1H, d, *J* = 15.2 Hz), 4.19 (2H, q, *J* = 7.2 Hz), 1.27 (3H, t, *J* = 7.1 Hz). ^13^C{^1^H}-NMR (100 MHz, CDCl_3_) δ: 166.9, 163.0 (d, *J* = 249.7 Hz), 144.3, 138.9, 132.3 (d, *J* = 3.4 Hz), 128.9 (d, *J* = 8.2 Hz), 126.0 (d, *J* = 2.4 Hz), 121.4, 115.8 (d, *J* = 21.9 Hz), 60.3, 14.3. ^19^F{^1^H}-NMR (376 MHz, CDCl_3_) δ: −110.4. The NMR data are in agreement with the values reported in the literature.^20^

### Ethyl (2*E*,4*E*)-5-(4-Bromophenyl)penta-2,4-dienoate (**15c**)

Compound **15c** was prepared from (*E*)-3-(4-bromophenyl)acrylaldehyde (450 mg, 2.13 mmol), triethylphosphonoacetate (621 mg, 2.77 mmol), NaH (128 mg, 3.19 mmol, 60% dispersion in mineral oil) and DME (7 mL) using the same procedure as used for compound **15a**. The crude product was purified using flash column chromatography (EtOAc/hexanes = 1:19) to afford **15c** (523 mg, 88%) as a white solid. *R*_*f*_*=* 0.42 (EtOAc/hexanes = 1:9) ^1^H NMR (400 MHz, CDCl_3_) δ: 7.46 (2H, d, *J* = 8.4 Hz), 7.41 (1H, ddd, *J* = 15.4, 8.7, 1.4 Hz), 7.30 (2H, d, *J* = 8.5 Hz), 6.89–6.75 (2H, m), 5.99 (1H, d, *J* = 15.3 Hz), 4.22 (2H, q, *J* = 7.1 Hz), 1.30 (2H, t, *J* = 7.1 Hz). ^13^C{^1^H}-NMR (100 MHz, CDCl_3_) δ: 167.0, 144.2, 138.9, 135.1, 132.1, 128.7, 127.1, 123.1, 122.1, 60.5, 14.4. The NMR data are in agreement with the values reported in the literature.^19^

### Ethyl (2*E*,4*E*)-5-(4-Methoxyphenyl)penta-2,4-dienoate (**15d**)

Compound **15d** was prepared from (*E*)-3-(4-methoxyphenyl)acrylaldehyde (500 mg, 3.08 mmol), triethylphosphonoacetate (1.04 g, 917 μL, 4.62 mmol), NaH (111 mg, 4.62 mmol, 60% dispersion in mineral oil) and DME (10 mL) using the same procedure as used for compound **15a**. The crude product was purified using flash column chromatography (EtOAc/hexanes = 1:19) to afford **15d** (557 mg, 78%) as a white solid. *R*_*f*_*=* 0.30 (EtOAc/hexanes = 1:19). ^1^H NMR (400 MHz, CDCl_3_) δ: 7.49–7.36 (3H, m), 6.93–6.82 (3H, m), 6.75 (1H, dd, *J* = 15.5, 10.8 Hz), 5.94 (1H, d, *J* = 15.3 Hz), 4.22 (2H, q, *J* = 7.1 Hz), 3.83 (3H, s), 1.31 (3H, t, *J* = 7.1 Hz). ^13^C{^1^H}-NMR (100 MHz, CDCl_3_) δ: 167.4, 160.6, 145.1, 140.2, 129.1, 128.8, 124.4, 120.2, 114.4, 60.4, 55.5, 14.5. The NMR data are in agreement with the values reported in the literature.^19^

### (2*E*,4*E*)-5-Phenylpenta-2,4-dienoic Acid (**16a**)

To a solution of compound **15a** (1.00 g, 4.94 mmol) in 1:2 mixture of MeOH and THF (15 mL) at 23 °C in a 100 mL round-bottom flask, 5 M aqueous solution of KOH (5 mL) was added, and the reaction mixture was allowed to stir at 23 °C. Progress of the reaction was monitored using TLC (EtOAc/hexanes = 1:1). Full consumption of **15a** was observed after 1 h. The solvents were removed directly under reduced pressure, and a white slurry was obtained. This white slurry was dissolved in fresh CHCl_3_, and then, conc. HCl was added dropwise until the pH of the solution turned 1–2. The organic phase was washed once with distilled water, and then the aqueous phase was extracted thrice with EtOAc. Organic phases were combined, dried over anhydrous Na_2_SO_4_, filtered, and concentrated under vacuum to give pure product **16a** (841 mg, 98%) as a shiny white solid. *R*_*f*_*=* 0.69 (EtOAc/hexanes = 1:1). ^1^H NMR (400 MHz, CDCl_3_) δ: 7.55 (1H, dd, *J* = 15.1, 9.7 Hz), 7.49 (2H, app d, *J* = 6.9 Hz), 7.39–7.31 (m, 3H), 6.99–6.87 (m, 2H), 6.01 (1H, d, *J* = 15.3 Hz). ^13^C{^1^H}-NMR (100 MHz, CDCl_3_) δ: 172.5, 147.1, 141.8, 136.0, 129.5, 129.0, 127.5, 126.1, 120.5. The NMR data are in agreement with the values reported in the literature.^21^

### (2*E*,4*E*)-5-(4-Fluorophenyl)penta-2,4-dienoic Acid (**16b**)

Compound **16b** was prepared using **15b** (620 mg, 2.82 mmol), 5 M KOH aqueous solution (10 mL), MeOH (5 mL) and THF (10 mL) using the same procedure as used for compound **16a**. After workup, **16b** (520 mg, 96%) was obtained as a white solid. *R*_*f*_*=* 0.55 (EtOAc/hexanes = 1:1). ^1^H NMR (400 MHz, CDCl_3_) δ: 7.55–7.44 (3H, m), 7.06 (2H, t, *J* = 8.2 Hz), 6.91 (1H, d, *J* = 15.7 Hz), 6.82 (1H, dd, *J* = 15.7, 10.7 Hz) 5.99 (1H, d, *J* = 15.1 Hz). ^13^C{^1^H}-NMR (100 MHz, DMSO-*d*_*6*_) δ: 167.6, 162.3 (d, *J* = 247.0 Hz), 143.9, 138.3, 132.7 (d, *J* = 3.1 Hz), 129.2 (d, *J* = 8.4 Hz), 126.6 (d, *J* = 2.4 Hz), 122.6, 115.7 (d, *J* = 21.6 Hz). ^19^F{^1^H}-NMR (376 MHz, CDCl_3_) δ: −109.9. The NMR data are in agreement with the values reported in the literature.^20,21^

### (2*E*,4*E*)-5-(4-Bromophenyl)penta-2,4-dienoic Acid (**16c**)

Compound **16c** was prepared using **15c** (520 mg, 1.86 mmol), 5 M KOH aqueous solution (6 mL), MeOH (3 mL) and THF (6 mL) using the same procedure as used for compound **16a**. After workup, **16c** (416 mg, 89%) was obtained as a white solid. *R*_*f*_*=* 0.38 (EtOAc/hexanes = 1:1). ^1^H NMR (400 MHz, DMSO-*d*_*6*_) δ: 7.59 (2H, d, *J* = 8.5 Hz), 7.52 (2H, d, *J* = 8.5 Hz), 7.33 (1H, dd, *J* = 15.1, 10.7 Hz), 7.15 (1H, dd, *J* = 15.5, 10.7 Hz), 7.03 (1H, d, *J* = 15.6 Hz), 6.03 (1H, d, *J* = 15.1 Hz). ^13^C{^1^H}-NMR (100 MHz, DMSO-*d*_*6*_) δ: 167.4, 144.0, 138.4, 135.3, 131.8, 129.0, 127.5, 122.8, 122.0. The NMR data are in agreement with the values reported in the literature.^22^

### (2*E*,4*E*)-5-(4-Methoxyphenyl)penta-2,4-dienoic Acid (**16d**)

Compound **16d** was prepared using **15d** (495 mg, 2.13 mmol), 5 M KOH aqueous solution (20 mL), MeOH (10 mL) and THF (20 mL) using the same procedure as used for compound **16a**. After workup, **15d** (425 mg, 98%) was obtained as a pale yellow solid. *R*_*f*_*=* 0.42 (EtOAc/hexanes = 1:1). ^1^H NMR (400 MHz, DMSO-*d*_*6*_) δ: 12.15 (1H, s), 7.51 (2H, d, *J* = 8.8 Hz), 7.32 (1H, ddd, *J* = 15.2, 9.0, 1.2 Hz), 7.02–6.92 (4H, m), 5.93 (1H, d, *J* = 15.2 Hz), 3.78 (3H, s). ^13^C{^1^H}-NMR (100 MHz, DMSO-*d*_*6*_) δ: 167.6, 160.0, 144.8, 139.7, 128.7, 124.3, 120.8, 114.3, 56.2. The NMR data are in agreement with the values reported in the literature.^23^

### Naphthalene-1,8-diyl (2*E*,2’*E*,4*E*,4’*E*)-Bis-5-phenylpenta-2,4-dienoate (**12a**)

Carboxylic acid **16a** (530 mg, 3.04 mmol) was dissolved in 8 mL of anhydrous CH_2_Cl_2_ under nitrogen at 23 °C. To this solution, 1,8-DHN (**17**) **(**162 mg, 1.01 mmol), DCC (667 mg, 3.23 mmol), and DMAP (37 mg, 0.30 mmol) were added sequentially. The reaction mixture was stirred at 23 °C for 24 h. The reaction mixture was filtered through Celite (CH_2_Cl_2_ was used to aid filtration). The organic phase was washed once with distilled water, and the aqueous phase was extracted thrice with CH_2_Cl_2_. Organic phases were combined, dried over anhydrous Na_2_SO_4_, filtered, and concentrated under vacuum. Purification by column chromatography (CH_2_Cl_2_/hexanes = 1:1) gave pure product **12a** (335 mg, 71%) as a white solid. **Note:** The compound is light-sensitive so it should be kept in the dark or wrapped with Al foil. Mp: 207–209 °C. *R*_*f*_*=* 0.22 (DCM/hexanes = 1:1). ^1^H NMR (400 MHz, CDCl_3_) δ: 7.80 (2H, d, *J* = 8.3 Hz,), 7.62 (2H, dd, *J* = 15.3 Hz, 10.3 Hz), 7.48 (2H, t, *J* = 7 0.9 Hz), 7.27–7.21 (6H, m), 7.19–7.14 (6H, m), 6.92–6.80 (4H, m), 6.15 (2H, d, *J* = 15.3 Hz). ^13^C{^1^H}-NMR (100 MHz, CDCl_3_) δ: 166.0, 146.9, 145.4, 142.0, 137.0, 135.7, 129.3, 128.9, 127.4, 127.0, 126.2, 126.0, 121.6, 120.8. FTIR ν_max_ (ATR, film)/cm^–1^: 3055, 3024, 1728, 1621, 1448, 1345, 1312, 1225, 1167. HRMS (ESI+) calcd for C_32_H_24_NaO_4_ [M + Na]^+^: 495.1567, found 495.1543.

### Naphthalene-1,8-diyl (2*E*,2’*E*,4*E*,4’*E*)-Bis(5-(4-fluorophenyl)penta-2,4-dienoate) (**12b**)

Compound **12b** was prepared using **16b** (179 mg, 0.93 mmol), 1,8-DHN (**17**) (50 mg, 0.31 mmol), DCC (204 mg, 0.99 mmol), DMAP (11 mg, 0.09 mmol) and anhydrous CH_2_Cl_2_ (10 mL) was the same procedure as used for compound **12a**. Purification by flash column chromatography (CH_2_Cl_2_/hexanes = 1:1) afforded **12b** (103 mg, 66%) as a white solid. **Note:** The compound is light-sensitive so it should be kept in the dark or wrapped with Al foil. Mp: 274–276 °C. *R*_*f*_*=* 0.63 (DCM/hexanes = 1:1). ^1^H NMR (400 MHz, CDCl_3_) δ: 7.82 (2H, d, *J* = 8.3 Hz), 7.58 (2H, dd, *J* = 15.3, 10.7 Hz), 7.49 (2H, t, *J* = 7.9 Hz), 7.23–7.16 (6H, m), 6.88–6.81 (6H, m), 6.72 (2H, dd, *J* = 15.6, 10.7 Hz), 6.14 (2H, d, *J* = 15.3 Hz). ^13^C{^1^H}-NMR (100 MHz, CDCl_3_) δ: 165.9, 163.4 (d, *J* = 250.7 Hz), 146.6, 145.4, 140.4, 137.0, 131.9 (d, *J* = 2.9 Hz), 129.0 (d, *J* = 8.2 Hz), 127.0, 126.2, 125.80, 125.78, 121.0, 120.8, 116.0 (d, *J* = 21.9 Hz). ^19^F{^1^H}-NMR (376 MHz, CDCl_3_) δ: −109.9. FTIR ν_max_ (ATR, film)/cm^–1^: 2957, 2920, 2851, 1744, 1723, 1625, 1597, 1508, 1234, 1156. HRMS (ESI+) calcd for C_32_H_22_NaO_4_F_2_ [M + Na]^+^: 531.1378, found 531.1380.

### Naphthalene-1,8-diyl (2*E*,2’*E*,4*E*,4’*E*)-Bis(5-(4-bromophenyl)penta-2,4-dienoate) (**12c**)

Compound **12c** was prepared using **16c** (50 mg, 0.21 mmol), 1,8-DHN (**17**) (11.2 mg, 0.069 mmol), DCC (43.3 mg, 0.21 mmol), DMAP (2.8 mg, 0.02 mmol) and anhydrous CH_2_Cl_2_ (5 mL) was the same procedure as used for compound **12a**. Purification by flash column chromatography (CH_2_Cl_2_:hexanes = 1:1) afforded **12c** (31.6 mg, 72%) as a white solid. **Note:** The compound is light-sensitive so it should be kept in the dark or wrapped with Al foil. Mp: 248–249 °C. *R*_*f*_*=* 0.45 (DCM/hexanes = 1:1). ^1^H NMR (400 MHz, CDCl_3_) δ: 7.82 (2H, d, *J* = 9.1 Hz), 7.57 (2H, ddd, *J* = 15.3, 7.1, 3.2 Hz), 7.51–7.47 (2H, m), 7.28 (4H, d, *J* = 8.5 Hz), 7.17 (2H, d, *J* = 8.3 Hz), 7.05 (4H, d, *J* = 8.5 Hz), 6.78–6.77 (4H, m), 6.15 (2H, d, *J* = 15.3 Hz). ^13^C{^1^H}-NMR (100 MHz, CDCl_3_) δ: 165.8, 146.4, 145.3, 145.1, 140.3, 137.0, 134.4, 132.1, 128.6, 127.0, 126.6, 126.2, 123.7, 121.5, 120.8. FTIR ν_max_ (ATR, film)/cm^–1^: 1726, 1620, 1599, 1581, 1484, 1313, 1264, 1122. HRMS (APCI+) calcd for C_32_H_23_O_4_^79^Br_2_ [M + H]^+^: 628.9958, found 628.9960; calcd for C_32_H_23_O_4_^79^Br^81^Br [M + H]^+^: 630.9938, found 630.9942; calcd for C_32_H_23_O_4_^81^Br_2_ [M + H]^+^: 632.9917, found 632.9917.

### 8-Hydroxynaphthalen-1-yl-(2*E*,4*E*)-5-phenylpenta-2,4-dienoate (**19**)

In a 25 mL round-bottom flask, **16a** (250 mg, 1.44 mmol) was dissolved in 3 mL of oxalyl chloride at 23 °C under nitrogen atmosphere. This solution was stirred in a preheated oil bath at 60 °C for 2 h. Afterward, the reaction mixture was cooled to room temperature, and all volatiles were removed by a rotary evaporator to give acyl chloride **18** (271 mg, 98%) as a yellow solid. In another 50 mL round-bottom flask, 1,8-DHN (**17**) (226 mg, 1.41 mmol) was dissolved in 5 mL of anhydrous THF under an inert atmosphere of nitrogen. This solution was cooled to 0 °C in an ice bath, and NaH (62 mg, 1.55 mmol, 60% dispersion in mineral oil) was added portionwise. The reaction mixture was then stirred at this temperature for 20 min. Acyl chloride **18** (271 mg, 1.41 mmol), which was prepared as described above, was dissolved in 5 mL of anhydrous THF, and this solution was added slowly to the reaction mixture. Then, the reaction mixture was stirred at 23 °C for 3 h. After full consumption of 1,8-DHN (**17**), the reaction mixture was quenched with 10 mL of saturated aqueous NH_4_Cl solution. The aqueous phase was extracted thrice with EtOAc. Combined organic phases were dried over anhydrous Na_2_SO_4_, filtered, and concentrated under vacuum. Purification by flash column chromatography (EtOAc/hexanes = 1:5) afforded pure product **19** (316 mg, 71%) as an orange solid. *R*_*f*_*=* 0.66 (EtOAc/hexanes = 1:5). ^1^H NMR (400 MHz, CDCl_3_) δ: 7.75–7.68 (2H, m), 7.51–7.48 (3H, m), 7.45–7.31 (6H, m), 7.24 (1H, d, *J* = 7.5 Hz), 7.04–6.93 (2H, m), 6.87 (1H, d, *J* = 6.9 Hz), 6.28 (1H, d, *J* = 15.2 Hz). ^13^C{^1^H}-NMR (100 MHz, CDCl_3_) δ: 164.7, 152.2, 148.1, 146.2, 142.9, 137.0, 135.8, 129.7, 129.0, 127.6, 127.3, 126.5, 125.9, 125.5, 120.3, 119.2, 118.5, 117.1, 111.5. FTIR ν_max_ (ATR, film)/cm^–1^: 3385, 3057, 1702, 1621, 1600, 1580, 1393, 1278, 1263, 1174. HRMS (APCI+) calcd for C_21_H_17_O_3_ [M + H]^+^: 317.1172, found 317.1175.

### 8-(((2*E*,4*E*)-5-(4-Methoxyphenyl)penta-2,4-dienoyl)oxy)naphthalen-1-yl (2*E*,4*E*)-(5-Phenylpenta-2,4-dienoate) (**12d**)

In an oven-dried 50 mL round-bottom flask, **16d** was dissolved in anhydrous CH_2_Cl_2_ (8 mL) under a nitrogen atmosphere at 23 °C. Monoester **19** (234 mg, 0.74 mmol), DCC (153 mg, 0.74 mmol), and DMAP (13.4 mg, 0.11 mmol) were added sequentially. The reaction mixture was stirred at 23 °C for 21 h. Then, the reaction mixture was quenched with saturated aqueous NH_4_Cl solution, and the aquesous phase was extracted thrice with CH_2_Cl_2_. The combined organic phase was dried over anhydrous Na_2_SO_4_, filtered and concentrated under vacuum. Purification by flash column chromatography (CH_2_Cl_2_/hexanes = 1:1) afforded pure product **12d** (178 mg, 48%) as a white solid. **Note:** The compound is light-sensitive so it should be kept in the dark or wrapped with Al foil. Mp: 177–179 °C. *R*_*f*_*=* 0.39 (CH_2_Cl_2_/hexanes = 1:1) ^1^H NMR (400 MHz, CDCl_3_) δ: 7.80 (2H, d, *J* = 8.3 Hz), 7.61 (2H, ddd, *J* = 14.9, 10.6, 4.1 Hz), 7.48 (2H, t, *J* = 7.9 Hz), 7.25–7.22 (3H, m), 7.21–7.16 (6H, m), 6.91–6.79 (3H, m), 6.74–6.65 (3H, m), 6.16 (1H, d, *J* = 15.2 Hz), 6.10 (1H, d, *J* = 15.3 Hz), 3.78 (3H, s). ^13^C{^1^H}-NMR (100 MHz, CDCl_3_) δ: 166.1, 166.0, 160.7, 147.4, 146.8, 145.5, 141.8, 137.0, 135.8, 131.0, 129.2, 128.9, 128.8, 128.6, 127.4, 126.91, 126.85, 126.2, 126.13, 126.08, 123.9, 121.7, 120.9, 120.8, 120.7, 119.5, 114.4, 55.4. FTIR ν_max_ (ATR, film)/cm^–1^: 3058, 2837, 1725, 1624, 1597, 1509, 1448, 1348, 1250, 1228, 1175. HRMS (APCI+) calcd for C_33_H_27_O_5_ [M + H]^+^: 503.1853, found 503.1859.

### 8-(Cinnamoyloxy)naphthalen-1-yl (2*E*,4*E*)-5-Phenylpenta-2,4-dienoate (**21**)

In a round-bottom flask, *trans-*cinnamic acid (50 mg, 0.34 mmol) was dissolved in 2 mL of oxalyl chloride at 23 °C under nitrogen atmosphere. This solution was stirred in a preheated oil bath at 60 °C for 1.5 h. Afterward, the reaction mixture was cooled to room temperature, and all volatiles were removed by a rotary evaporator to give cinnamoyl chloride (**21**) (51 mg, 90%). In another 50 mL round-bottom flask, monoester **19** (92 mg, 0.29 mmol) was dissolved in 3 mL of anhydrous THF under an inert atmosphere of nitrogen. To this solution, which was cooled to 0 °C in an ice bath, was added a solution of the cinnamoyl chloride (**21**) in 2 mL of anhydrous THF. Afterward, to the reaction mixture, NaH (13 mg, 0.32 mmol, 60% dispersion in mineral oil) was added portionwise at 0 °C. The reaction mixture was then stirred at this temperature for 20 min, and at 23 °C for 3 h. At the end of this time, the reaction mixture was quenched with 10 mL of saturated aqueous NH_4_Cl solution. The aqueous phase was extracted thrice with EtOAc. The combined organic phases were dried over anhydrous Na_2_SO_4_, filtered, and concentrated under vacuum. Purification by flash column chromatography (CH_2_Cl_2_/hexanes = 1:2) afforded pure product **21** (90 mg, 70%) as a white solid. Mp: 156–158 °C. *R*_*f*_*=* 0.34 (CH_2_Cl_2_/hexanes = 1:1). ^1^H NMR (400 MHz, CDCl_3_) δ: 7.89 (1H, d, *J* = 16.1 Hz), 7.83 (2H, dt, *J* = 8.4, 1.3 Hz), 7.64 (1H, ddd, *J* = 15.3, 11.1, 0.4 Hz), 7.54–7.49 (4H, m), 7.36–7.33 (3H, m), 7.27–7.17 (7H, m), 6.84 (1H, d, *J* = 15.6 Hz), 6.68 (1H, d, *J* = 16.1 Hz), 6.57 (1H, dd, *J* = 15.7, 11.4 Hz), 6.15 (1H, d, *J* = 15.3 Hz). ^13^C{^1^H}-NMR (100 MHz, CDCl_3_) δ: 165.9, 146.85, 146.79, 145.35, 145.33, 141.7, 137.0, 135.8, 134.1, 130.7, 129.4, 129.1, 128.8, 128.4, 127.5, 127.0, 126.9, 126.18, 126.15, 126.0, 121.5, 120.8, 120.73, 120.72, 117.7. FTIR ν_max_ (ATR, film)/cm^–1^: 3059, 3026, 1729, 1623, 1603, 1576, 1448, 1344, 1328, 1229, 1174. HRMS (APCI+) calcd for C_30_H_23_O_4_ [M + H]^+^: 447.1591, found 447.1570.

### General Procedure A for the Photochemical [2 + 2] Cycloaddition in Solid State

Cycloaddition precursor (ca. 20 mg) was placed between two quartz microscopic glass slides as a solid powder (for ground samples, grinding was done in mortar and pestle for 5 min), and irradiated inside a UV gel nail dryer having four 9-W UV-A (365 nm) fluorescent lamps. For the irradiation experiments for 8, 16, and 24 h, the reaction powder was mixed with a spatula every 4 h, and for the irradiation experiments which required 48 h, the reaction powder was mixed every 8 h. At the end of the reaction, the solid mixture was transferred into a clean vial using CHCl_3_. The diastereomeric ratio was determined via the ^1^H NMR analysis of the crude mixture. Purification was performed by flash column chromatography.

### General Procedure B for the Photochemical [2 + 2] Cycloaddition in Solution Phase

Cycloaddition precursor (ca. 20 mg) was dissolved in 2 mL of CHCl_3_ in a quartz test tube, and irradiated inside a UV gel nail dryer having four 9-W UV-A (365 nm) fluorescent lamps. Progress of the reaction was monitored using TLC. After the reaction is over, the solvent was removed under reduced pressure, and the diastereomeric ratio was determined via the ^1^H NMR analysis of the crude mixture. Purification was performed by flash column chromatography.

### (8a*R*,9*S*,10*R*,10a*S*)-9,10-Di((*E*)-styryl)-8a,9,10,10a-tetrahydrocyclobuta[*g*]naphtho[1,8 *bc*][1,5]dioxonine-8,11-dione (**22a**)

Cycloadduct **22a** was synthesized using diester **12a** (20.3 mg, 0.043 mmol) following General Procedure A with an irradiation time of 48 h (crude dr = 11:1). Purification by flash column chromatography (CH_2_Cl_2_/hexanes = 1:1) afforded product **22a** (10.5 mg, 52%, dr = 25:1) as an orange-yellow solid.

In a second experiment, cycloadduct **22a** was synthesized using diester **12a** (24.6 mg, 0.052 mmol) following General Procedure B with an irradiation time of 4 h (crude dr = 8:1). Purification by flash column chromatography (CH_2_Cl_2_/hexanes = 1:1) afforded product **22a** (21.7 mg, 88%, dr = 97:3) as an orange-yellow solid.

In a third experiment, diester **12a** (500 mg, 1.06 mmol) was dissolved in 15 mL of CHCl_3_ in a beaker and irradiated inside a UV gel nail dryer having four 9-W UV-A (365 nm) fluorescent lamps. Progress of the reaction was monitored using TLC (CH_2_Cl_2_/hexanes = 1:1). After 7 h, the solvent was removed under reduced pressure, and the diastereomeric ratio was determined via the ^1^H NMR analysis of the crude mixture (dr = 13:1). Purification by flash column chromatography (CH_2_Cl_2_/hexanes = 1:1) afforded product **22a** (304 mg, 61%) as a yellow solid, along with recovered starting material **12a** (53 mg). *R*_*f*_*=* 0.48 (CH_2_Cl_2_/hexanes = 1:1). ^1^H NMR (400 MHz, CDCl_3_) δ: 7.81 (2H, d, *J* = 8.4 Hz), 7.52 (2H, t, *J* = 7.9 Hz), 7.39 (4H, d, *J* = 7.2 Hz), 7.34–7.28 (6H, m), 7.27–7.23 (2H, m), 6.59 (2H, d, *J* = 15.9 Hz), 6.39 (2H, ddd, *J* = 15.9, 5.2, 2.4 Hz), 4.15–4.11 (2H, m), 3.87 (2H, app d, *J* = 5.1 Hz). ^13^C{^1^H}-NMR (100 MHz, CDCl_3_) δ: 170.1, 145.5, 137.1, 136.8, 132.6, 128.8, 127.9, 127.6, 127.1, 126.53, 126.51, 121.1, 119.6, 45.0, 42.1. FTIR ν_max_ (ATR, film)/cm^–1^: 3058, 3026, 2925, 1764, 1607, 1577, 1494, 1448, 1364, 1217, 1176. HRMS (APCI+) calcd. for C_32_H_25_O_4_ [M + H]^+^: 473.1747, found 473.1758.

### (8a*R*,9*S*,10*R*,10a*S*)-9,10-Bis((*E*)-4-fluorostyryl)-8a,9,10,10a-tetrahydrocyclobuta[*g*]naphtho[1,8-*bc*][1,5]dioxonine-8,11-dione (**22b**)

Cycloadduct **22b** was synthesized using diester **12b** (20.4 mg, 0.040 mmol) following General Procedure A with an irradiation time of 24 h (crude dr = 6:1). Purification by flash column chromatography (CH_2_Cl_2_/hexanes = 1:1) afforded product **22b** (20.2 mg, 99%, dr = 6:1) as an orange-yellow solid.

In a second experiment, cycloadduct **22b** was synthesized using diester **12b** (20.1 mg, 0.039 mmol) following General Procedure B with an irradiation time of 2 h (crude dr = 3:1). Purification by flash column chromatography (CH_2_Cl_2_/hexanes = 1:1) afforded product **22b** (19.3 mg, 96%) as an orange-yellow solid. *R*_*f*_*=* 0.43 (1:5 EtOAc: hexanes). ^1^H NMR (400 MHz, CDCl_3_) δ: 7.81 (2H, d, *J* = 8.2 Hz), 7.51 (2H, t, *J* = 7.9 Hz), 7.34 (4H, dd, *J* = 8.4, 5.5 Hz), 7.29 (2H, d, *J* = 7.4 Hz), 7.01 (4H, t, *J* = 8.6 Hz), 6.54 (2H, d, *J* = 15.9 Hz), 6.28 (2H, ddd, *J* = 15.8, 5.1, 2.2 Hz), 4.11 (2H, br s), 3.85 (2H, app d, *J* = 4.9 Hz). ^13^C{^1^H}-NMR (100 MHz, CDCl_3_) δ: 170.0, 162.6 (d, *J* = 247.3 Hz), 145.4, 132.9 (d, *J* = 3.4 Hz), 131.5, 128.0 (d, *J* = 8.0 Hz), 127.2 (d, *J* = 2.0 Hz), 127.1, 126.5, 121.1, 115.9, 115.6, 114.9 (d, *J* = 21.2 Hz), 45.0, 42.1. ^19^F{^1^H}-NMR (376 MHz, CDCl_3_) δ: **-**112.4. FTIR ν_max_ (ATR, film)/cm^–1^: 3041, 2956, 2927, 1759, 1605, 1507, 1363, 1213, 1175. HRMS (APCI+): calcd for C_32_H_23_O_4_F_2_ [M + H]^+^: 509.1559, found 509.1556.

### (8a*R*,9*S*,10*R*,10a*S*)-9,10-Bis((*E*)-4-bromostyryl)-8a,9,10,10a-tetrahydrocyclobuta[*g*]naphtho[1,8-*bc*][1,5]dioxonine-8,11-dione (**22c**)

Cycloadduct **22c** was synthesized using diester **12c** (18.2 mg, 0.029 mmol) following General Procedure A with an irradiation time of 24 h (crude dr = 6:1). Purification by flash column chromatography (CH_2_Cl_2_/hexanes = 1:1) afforded product **22c** (3.1 mg, 17%, dr = 5:1) as a yellow solid.

In a second experiment, cycloadduct **22c** was synthesized using diester **12c** (15.0 mg, 0.024 mmol) following General Procedure B with an irradiation time of 1.5 h. Purification by flash column chromatography (CH_2_Cl_2_/hexanes = 1:1) afforded product **22c** (9.4 mg, 63%, dr = 11:1) as a yellow solid. *R*_*f*_*=* 0.56 (CH_2_Cl_2_/hexanes = 1:1) ^1^H NMR (400 MHz, CDCl_3_) δ: 7.81 (2H, d, *J* = 8.0 Hz), 7.51 (2H, t, *J* = 7.8 Hz), 7.44 (4H, d, *J* = 8.4 Hz), 7.28 (2H, d, *J* = 7.5 Hz), 7.23 (4H, d, *J* = 8.5 Hz), 6.52 (2H, d, *J* = 15.9 Hz), 6.34 (2H, ddd, *J* = 15.8, 5.2, 2.4 Hz), 4.10 (2H, br s), 3.85 (2H, app d, *J* = 5.1 Hz). ^13^C{^1^H}-NMR (100 MHz, CDCl_3_) δ: 169.9, 145.4, 137.1, 135.6, 132.0, 131.6, 130.6, 128.2, 128.0, 127.1, 126.5, 121.8, 121.1, 44.9, 42.0. FTIR ν_max_ (ATR, film)/cm^–1^: 2953, 2923, 2852, 1764, 1607, 1487, 1460, 1364, 1214, 1176. HRMS (APCI+) calcd for C_32_H_23_O_4_^79^Br_2_ [M + H]^+^: 628.9958, found 628.9944; calcd for C_32_H_23_O_4_^79^Br^81^Br [M + H]^+^: 630.9938, found 630.9921; calcd for C_32_H_23_O_4_^81^Br_2_ [M + H]^+^: 632.9917, found 632.9915.

### (8a*R*,9*S*,10*R*,10a*S*)-9((*E*))-4-Methoxystyryl)-10-((*E*))-styryl)-8a,9,10,10a-tetrahydrocyclobuta[*g*]naphtho[1,8-*bc*][1,5]dioxonine-8,11-dione (**22d**)

Cycloadduct **22d** was synthesized using diester **12d** (20.6 mg, 0.041 mmol) following General Procedure A with an irradiation time of 24 h (crude dr = 13:1). Purification by flash column chromatography (CH_2_Cl_2_/hexanes = 1:1) afforded product **22d** (11.0 mg, 53%, dr = 20:1) as a yellow solid.

In a second experiment, cycloadduct **22d** was synthesized using diester **12d** (20.4 mg, 0.041 mmol) following General Procedure B with an irradiation time of 1 h (crude dr = 8:1). Purification by flash column chromatography (CH_2_Cl_2_/hexanes = 1:1) afforded product **22d** (17.1 mg, 84%, dr = 16:1) as a yellow solid. *R*_*f*_*=* 0.53 (CH_2_Cl_2_/hexanes = 1:1) ^1^H NMR (400 MHz, CDCl_3_) δ: 7.81 (2H, d, *J* = 7.6 Hz), 7.51 (2H, d, *J* = 7.6 Hz), 7.38 (2H, app d, *J* = 7.1 Hz), 7.34–7.28 (7H, m), 6.86 (2H, d, *J* = 8.7 Hz), 6.58 (1H, d, *J* = 15.8 Hz), 6.53 (1H, d, *J* = 15.8 Hz), 6.41–6.36 (1H, m), 6.26–6.21 (1H, m), 4.12–4.09 (2H, m), 3.85 (2H, d, *J* = 4.7 Hz), 3.81 (3H, s). ^13^C{^1^H}-NMR (100 MHz, CDCl_3_) δ: 170.19, 170.17, 159.5, 145.5, 137.1, 136.9, 132.5, 132.0, 129.6, 129.0, 128.8, 128.0, 127.8, 127.72, 127.70, 127.0, 126.52, 126.50, 125.3, 121.0, 120.7, 119.6, 114.2, 113.4, 55.5, 45.2, 45.0, 42.20, 42.16. FTIR ν_max_ (ATR, film)/cm^–1^**:** 3058, 2954, 2851, 1761, 1606, 1577, 1510, 1364, 1216, 1174. HRMS (APCI+) calcd for C_33_H_26_NaO_5_ [M + Na]^+^: 525.1672, found 525.1677.

### (8a*R*,9*S*,10*R*,10a*S*)-9-Phenyl-10-((*E*)-styryl)-8a,9,10,10a-tetrahydrocyclobuta[*g*]naphtho[1,8-*bc*][1,5]dioxonine-8,11-dione (**23**)

Cycloadduct **23** was synthesized using diester **21** (20.0 mg, 0.045 mmol) following General Procedure A with an irradiation time of 24 h (crude dr = 4:1). Purification by flash column chromatography (CH_2_Cl_2_/hexanes = 1:1) afforded product **23** (9.3 mg, 47%, dr = 14:1) as an orange-yellow solid.

In a second experiment, cycloadduct **23** was synthesized using diester **21** (20.0 mg, 0.045 mmol) following General Procedure B with an irradiation time of 3.5 h. Purification by flash column chromatography (CH_2_Cl_2_/hexanes = 1:1) afforded product **23** (8.4 mg, 42%; dr = 9:1) as a yellow solid. *R*_*f*_*=* 0.50 (CH_2_Cl_2_/hexanes = 1:1) ^1^H NMR (400 MHz, CDCl_3_) δ: 7.84 (2H, d, *J* = 8.3 Hz), 7.57–7.52 (2H, m), 7.40 (2H, t, *J* = 7.5 Hz), 7.35–7.28 (7H, m), 7.24–7.18 (3H, m), 6.53 (1H, d, *J* = 15.9 Hz), 5.97 (1H, dd, *J* = 15.8, 8.2 Hz), 4.66 (1H, dd, *J* = 9.2, 8.4 Hz), 4.30–4.21 (2H, m), 3.92 (1H, dd, *J* = 10.4, 5.8 Hz). ^13^C{^1^H}-NMR (100 MHz, CDCl_3_) δ: 170.2, 170.0, 145.52, 145.50, 138.4, 137.1, 137.0, 132.4, 128.8, 128.7, 128.0, 127.9, 127.7, 127.2, 127.06, 127.05, 126.6, 126.5, 126.4, 121.1, 119.7, 45.4, 44.5, 43.7, 42.2. FTIR ν_max_ (ATR, film)/cm^–1^: 3058, 3027, 2925, 1764, 1607, 1495, 1449, 1364, 1213, 1177. HRMS (APCI+) calcd for C_30_H_23_O_4_ [M + H]^+^: 447.1591, found 447.1587.

### Dimethyl (1*R*,2*S*,3*R*,4*S*)-3,4-Di((*E*)-styryl)cyclobutane-1,2-dicarboxylate (**13a**)

Cycloadduct **22a** (11.4 mg, 0.024 mmol) was dissolved in a mixture of MeOH (4 mL) and THF (1 mL) in a vial at 23 °C. NaOMe (3.3 mg, 0.068 mmol) was added to this solution. Upon the addition of NaOMe, the color of the solution turned from yellow to orange immediately. The reaction mixture was stirred at 23 °C, and the reaction progress, which was monitored by TLC (EtOAc/hexanes = 1:5), indicated completion of the reaction after 8 h. Afterward, all volatiles were directly evaporated. Purification by flash column chromatography (EtOAc/hexanes = 1:5) gave product **13a** (8.2 mg, 89% yield) as an orange solid. *R*_*f*_*=* 0.48 (CH_2_Cl_2_/hexanes = 1:1). ^1^H NMR (400 MHz, CDCl_3_) δ: 7.35–7.33 (4H, m), 7.29 (4H, t, *J* = 7.5 Hz), 7.21 (2H, app t, *J* = 7.1 Hz), 6.50 (2H, d, *J* = 15.8 Hz), 6.29 (2H, ddd, *J* = 15.8, 5.3, 2.4 Hz), 3.74 (6H, s), 3.73–3.67 (2H, m), 3.45 (2H, app d, *J* = 5.4 Hz). ^13^C{^1^H}-NMR (100 MHz, CDCl_3_) δ: 172.9, 137.0, 132.3, 128.7, 128.1, 127.7, 126.5, 52.2, 44.0, 43.1. FTIR ν_max_ (ATR, film)/cm^–1^: 2952, 2850, 1735, 1602, 1495, 1436, 1365, 1264, 1170. HRMS (ESI+) calcd for C_24_H_25_O_4_ [M + H]^+^: 377.1747, found 377.1743.

### Dimethyl (1*R*,2*S*,3*R*,4*S*)-3,4-Di((*E*)-4-fluorostyryl)cyclobutane-1,2-dicarboxylate (**13b**)

Cycloadduct **22b** (20.2 mg, 0.040 mmol) was dissolved in a mixture of MeOH (3 mL) and THF (3 mL) in a vial at 23 °C. NaOMe (5.4 mg, 0.1 mmol) was added to this solution. Upon the addition of NaOMe, the color of the solution turned from yellow to orange immediately. The reaction mixture was stirred at 23 °C, and the reaction progress, which was monitored by TLC (EtOAc/hexanes = 1:5), indicated completion of the reaction after 8 h. Afterward, all volatiles were directly evaporated. Purification by flash column chromatography (EtOAc/hexanes = 1:5) gave product **13b** (10.8 mg, 65% yield, dr = 10:1) as a yellow solid. *R*_*f*_*=* 0.33 (EtOAc/hexanes = 1:5). ^1^H NMR (400 MHz, CDCl_3_) δ: 7.32–7.27 (4H, m), 6.97 (4H, t, *J* = 8.7 Hz), 6.45 (2H, d, *J* = 15.8 Hz), 6.18 (2H, ddd, *J* = 15.8, 5.4, 2.5 Hz), 3.73 (6H, s), 3.73–3.71 (2H, m), 3.42 (2H, app d, *J* = 5.4 Hz). ^13^C{^1^H}-NMR (100 MHz, CDCl_3_) δ: 172.9, 162.5 (d, *J* = 246.9 Hz), 131.2, 127.9 (d, *J* = 8.0 Hz), 127.8 (d, *J* = 2.0 Hz), 125.2, 115.6 (d, *J* = 21.6 Hz), 52.2, 44.0, 43.0. ^19^F{^1^H}-NMR (376 MHz, CDCl_3_) δ: **-**113.4 (s). FTIR ν_max_ (ATR, film)/cm^–1^: 3054, 2953, 1736, 1601, 1508, 1264, 1226, 1158. HRMS (ESI+) calcd for C_24_H_23_O_4_F_2_ [M + H]^+^: 413.1559, found 413.1546.

### Dimethyl (1*R*,2*S*,3*R*,4*S*)-3,4-Di((*E*)-4-bromostyryl)cyclobutane-1,2-dicarboxylate (**13c**)

Cycloadduct **22c** (9.4 mg, 0.015 mmol) was dissolved in a mixture of MeOH (1.5 mL) and THF (1.5 mL) in a vial at 23 °C. NaOMe (2.0 mg, 0.038 mmol) was added to this solution. Upon the addition of NaOMe, the color of the solution turned from yellow to orange immediately. The reaction mixture was stirred at 23 °C, and the reaction progress, which was monitored by TLC (EtOAc/hexanes = 1:5), indicated completion of the reaction after 3 h. Afterward, all volatiles were directly evaporated. Purification by flash column chromatography (EtOAc/hexanes = 1:5) gave product **13c** (5.1 mg, 65% yield, dr = 10:1) as a yellow solid. *R*_*f*_*=* 0.38 (EtOAc/hexanes = 1:5) ^1^H NMR (400 MHz, CDCl_3_) δ: 7.40 (4H, d, *J* = 8.5 Hz), 7.18 (4H, d, *J* = 8.5 Hz), 6.43 (2H, d, *J* = 15.9 Hz), 6.24 (2H, ddd, *J* = 15.8, 5.4, 2.2 Hz), 3.73 (8H, m, overlapping signals), 3.42 (2H, app d, *J* = 5.3 Hz). ^13^C{^1^H}-NMR (100 MHz, CDCl_3_) δ: 172.8, 135.9, 131.9, 131.3, 128.9, 128.0, 121.5, 52.3, 43.9, 43.0. FTIR ν_max_ (ATR, film)/cm^–1^: 2950, 2924, 1736, 1487, 1434, 1364, 1274, 1167. HRMS (APCI+) calcd for C_24_H_23_O_4_^79^Br_2_ [M + H]^+^: 532.9958, found 532.9953; calcd for C_24_H_23_O_4_^79^Br^81^Br [M + H]^+^: 534.9938, found 534.9944; calcd for C_24_H_23_O_4_^81^Br_2_ [M + H]^+^: 536.9917, found 536.9914.

### Dimethyl (1*S*,2*R*,3*S*,4*R*)-3-((*E*)-4-Methoxystyryl)-4-((*E*)-styryl)cyclobutane-1,2-dicarboxylate (**13d**)

Cycloadduct **22d** (17.1 mg, 0.034 mmol) was dissolved in a mixture of MeOH (2 mL) and THF (2 mL) in a vial at 23 °C. NaOMe (4.6 mg, 0.085 mmol) was added to this solution. Upon the addition of NaOMe, the color of the solution turned from yellow to orange immediately. The reaction mixture was stirred at 23 °C, and the reaction progress, which was monitored by TLC (EtOAc/hexanes = 1:5), indicated completion of the reaction after 6 h. Afterward, all volatiles were directly evaporated. Purification by flash column chromatography (EtOAc/hexanes = 1:5) gave product **13d** (12.3 mg, 89% yield) as a yellow solid. *R*_*f*_*=* 0.38 (EtOAc/hexanes = 1:3) ^1^H NMR (400 MHz, CDCl_3_) δ: 7.35–7.28 (5H, m), 7.23–7.17 (2H, m), 6.82 (2H, d, *J* = 8.7 Hz), 6.49 (1H, d, *J* = 15.9 Hz), 6.44 (1H, d, *J* = 15.8 Hz), 6.28 (1H, dd, *J* = 15.8, 7.4 Hz), 6.14 (1H, dd, *J* = 15.8, 7.6 Hz), 3.79 (3H, s), 3.73 (6H, s), 3.73–3.71 (2H, m), 3.43 (2H, d, *J* = 5.1 Hz). ^13^C{^1^H}-NMR (100 MHz, CDCl_3_) δ: 173.0, 159.4, 137.1, 132.1, 131.7, 129.9, 128.70, 128.66, 128.3, 127.6, 126.5, 125.9, 114.2, 113.1, 55.4, 52.2, 44.2, 44.0, 43.12, 43.08. FTIR ν_max_ (ATR, film)/cm^–1^: 3058, 2926, 1760, 1730, 1602, 1366, 1226, 1174. HRMS (APCI+) calcd for C_25_H_27_O_5_ [M + H]^+^: 407.1853, found 407.1859.

### Dimethyl (1*S*,2*R*,3*S*,4*R*)-3-Phenyl-4-((*E*)-styryl)cyclobutane-1,2-dicarboxylate (**24**)

Cycloadduct **23** (8.4 mg, 0.019 mmol) was dissolved in a mixture of MeOH (1.5 mL) and THF (1.5 mL) in a vial at 23 °C. NaOMe (2.6 mg, 0.048 mmol) was added to this solution. Upon the addition of NaOMe, the color of the solution turned from yellow to orange immediately. The reaction mixture was stirred at 23 °C, and the reaction progress, which was monitored by TLC (EtOAc/hexanes = 1:5), indicated completion of the reaction after 2.5 h. Afterward, all volatiles were directly evaporated. Purification by flash column chromatography (EtOAc/hexanes = 1:5) gave product **24** (4.8 mg, 73%) as a yellow solid. *R*_*f*_*=* 0.27 (EtOAc/hexanes = 1:5). ^1^H NMR (400 MHz, CDCl_3_) δ: 7.33–7.29 (2H, m), 7.23–7.16 (6H, m), 7.13–7.11 (2H, m), 6.41 (1H, d, *J* = 15.8 Hz), 5.84 (1H, dd, *J* = 15.8, 8.5 Hz), 4.32 (1H, t, *J* = 8.9 Hz), 3.85–3.80 (2H, m), 3.76 (3H, s), 3.73 (3H, s), 3.45 (1H, dd, *J* = 9.9, 5.4 Hz). ^13^C{^1^H}-NMR (100 MHz, CDCl_3_) δ: 173.1, 173.0, 138.9, 137.1, 132.0, 128.7, 128.6, 128.5, 127.7, 127.5, 126.8, 126.4, 52.3, 52.2, 44.4, 44.3, 43.2, 42.9. FTIR ν_max_ (ATR, film)/cm^–1^: 2951, 2924, 1733, 1601, 1496, 1449, 1366, 1206, 1167. HRMS (APCI+) calcd for C_22_H_23_O_5_ [M + H]^+^: 351.1591, found 351.1604.

### (1*R*,2*S*,3*R*,4*S*)-3,4-Di((*E*)-styryl)cyclobutane-1,2-dicarboxylic Acid (**25**)

Cycloadduct **22a** (8.4 mg, 0.018 mmol) was dissolved in 2 mL of THF in a 25 mL round-bottom flask at 23 °C. Then distilled water (1 mL) and KOH (19 mg, 0.34 mmol) were added to the reaction vessel sequentially, and the reaction mixture was stirred at 23 °C. Reaction progress was monitored using TLC (EtOAc/hexanes = 1:1). After 2 h, full consumption of **22a** was observed. The reaction mixture was quenched with 1 M HCl solution in an ice bath until the pH became 1–2. The aqueous phase was extracted thrice with EtOAc. The combined organic phases were dried over anhydrous Na_2_SO_4_, filtered, and concentrated under vacuum. Purification by flash column chromatography (EtOAc/hexanes = 1:1, then 0.5% (v/v) AcOH in EtOAc/hexanes = 1:1) gave product **25** (4.3 mg, 69%) as a yellow solid. *R*_*f*_***=*** 0.13 (EtOAc/hexanes = 1:1 + 0.5% (v/v) Acetic acid). ^1^H NMR (400 MHz, CD_3_OD) δ: 7.36 (4H, d, *J* = 7.5 Hz), 7.26 (4H, t, *J* = 7.5 Hz), 7.17 (2H, t, *J* = 7.5 Hz), 6.51 (2H, d, *J* = 15.9 Hz), 6.41 (2H, ddd, *J* = 7.2, 4.8, 1.9 Hz), 3.66 (2H, br s), 3.48 (2H, app d, *J* = 4.7 Hz) (Signal at 4.88 ppm originates from water and the signal at 5.49 is from CH_2_Cl_2_). ^13^C{^1^H}-NMR (100 MHz, CD_3_OD) δ: 176.5, 138.6, 132.9, 130.0, 129.5, 128.3, 127.3, 45.3, 44.5. FTIR ν_max_ (ATR, film)/cm^–1^: 3028, 2955, 2921, 2851, 1707, 1600, 1449, 1258, 1176. HRMS (ESI-) Calcd for C_22_H_19_O_4_ [M-H]^−^: 347.1288, found 347.1293.

### (1*R*,2*S*,3*R*,4*S*)-3,4-Di((*E*)-styryl)cyclobutane-1,2-diyl)dimethanol (**26**)

Cycloadduct **22a** (11.8 mg, 0.025 mmol) was dissolved in THF (4 mL) in a round-bottom flask under nitrogen. This solution was cooled to 0 °C in an ice bath. LiAlH_4_ (9.5 mg, 0.25 mmol) was added to this cooled solution, and the reaction mixture was then stirred at 23 °C for 2 h. After full consumption of **22a**, the reaction was quenched with 10 mL of water, and the aqueous phase was extracted thrice with EtOAc. The organic phases were combined, dried over anhydrous Na_2_SO_4_, filtered, and concentrated under vacuum. Purification by flash column chromatography (5% MeOH in CH_2_Cl_2_) afforded product **26** (5.2 mg, 65%) as a pale yellow solid. *R*_*f*_*=* 0.52 (5% MeOH in CH_2_Cl_2_). ^1^H NMR (400 MHz, CDCl_3_) δ: 7.34–7.28 (8H, m), 7.19 (2H, tt, *J* = 7.1, 2.2 Hz), 6.41 (2H, d, *J* = 15.8 Hz), 6.34 (2H, ddd, *J* = 15.9, 4.9, 2.2 Hz), 3.96 (2H, t, *J* = 10.8 Hz), 3.83 (2H, dd, *J* = 10.8, 2.7 Hz), 3.02–2.99 (2H, m), 2.80–2.76 (4H, m). ^13^C{^1^H}-NMR (100 MHz, CDCl_3_) δ: 137.4, 130.9, 130.1, 128.7, 127.4, 126.3, 62.5, 42.0, 41.6. FTIR ν_max_ (ATR, film)/cm^–1^: 3313, 3025, 2853, 1665, 1599, 1492, 1450, 1260. HRMS (APCI+) calcd for C_22_H_25_O_2_ [M + H]^+^: 321.1849, found 321.1847.

### Crystallization of Compounds **12a**, **16a**, and **22a** for Single-Crystal XRD Analysis

Each compound (**12a**, **16a** and **22a**; ca. 5–10 mg) was dissolved in 1.0 mL of CH_2_Cl_2_ in a 2 mL vial, which was placed inside a 20 mL scintillation vial containing ca. 5 mL of pentane. The outer vial was sealed with a screw cap, and crystallization was carried out via vapor-diffusion technique under dark inside a cupboard at room temperature.

## Data Availability

The data underlying this study are available in the published article and its Supporting Information.

## Supporting Information Available

Tables S1–S5, Figures S1–S7, Scheme S1, NMR spectra for all synthesized compounds, X-ray analysis data of **12a**, **16a** and **22a** (PDF)
FAIR data, including the primary NMR FID files, for compounds **12a**-**d**, **13a**-**d**, **15a**-**d**, **16a**-**d**, **19**, **21**, **22a**-**d**, and **23**-**26** (ZIP)
