# Supplementary material for: Template-Directed Selective Photodimerization Reactions of 5-Arylpenta-2,4-dienoic Acids
Source: J Org Chem. 2024 Jul 10;89(14):10409–18. doi: 10.1021/acs.joc.4c01374 (PMC11267613; doi:10.1021/acs.joc.4c01374)
Supplement: Supplementary file 1 — jo4c01374_si_001.pdf [file jo4c01374_si_001.pdf]

Template-Directed Selective Photodimerization Reactions of 5-Arylpenta-2,4-dienoic Acids

Supporting Information

Badar Munir,[†] Bilge Banu Yagci,[†] Yunus Zorlu,[*,§] and Yunus E. Türkmen*[,†,‡]

[†]*Department of Chemistry, Faculty of Science, Bilkent University, Ankara, 06800,* Türkiye

[§]*Department of Chemistry, Gebze Technical University, Gebze, Kocaeli, 41400, Türkiye*

[‡]*UNAM — National Nanotechnology Research Center, Institute of Materials Science and Nanotechnology, Bilkent University, Ankara, 06800, Türkiye*

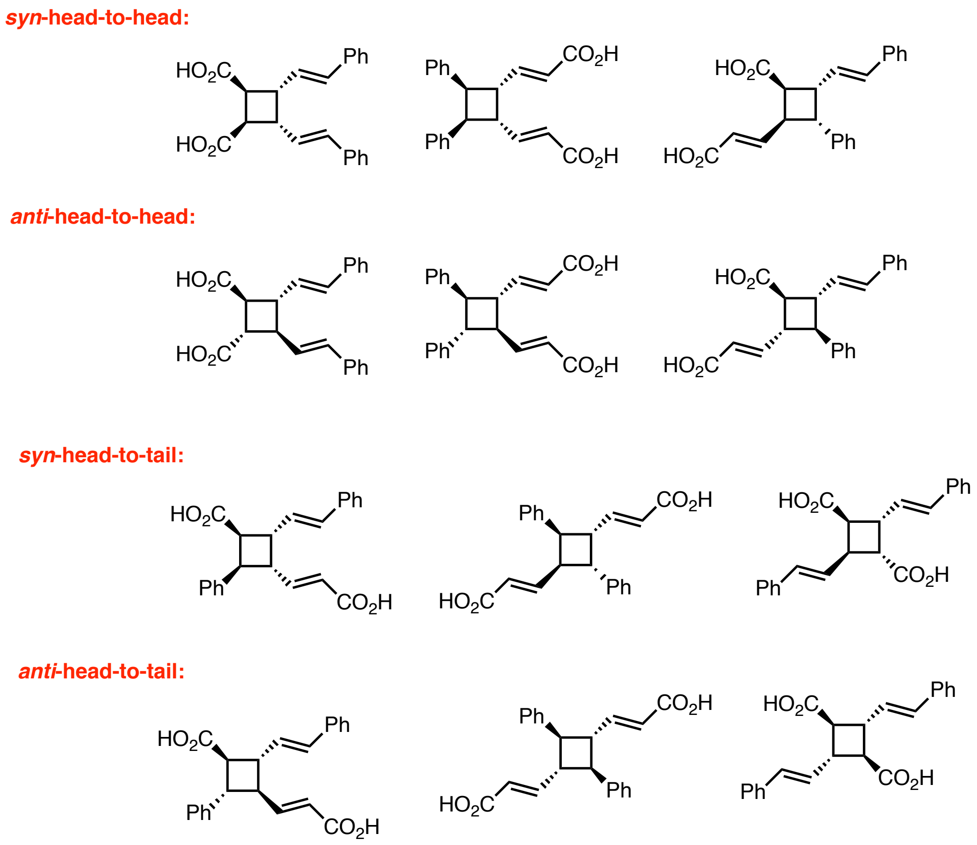

Figure S1. Structures of possible mono [2+2] cycloaddition products of (2*E*,4*E*)-5-phenylpenta-2,4-dienoic acid (**16a**) (enantiomers not shown).

Table S1. Studies on the photochemical [2+2] cycloaddition of diester **12a**.

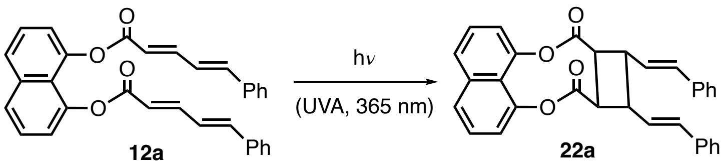

entry	sample	time (h)	yield (%)[a]	dr[b]
1	powder	16	30	8:1
2	powder	48	52	8:1
3	ground powder	16	32	10:1
4	ground powder	48	53	10:1
5	solution ($CHCl_3$)	4	88	8:1
6	solution ($CHCl_3$, 1.0 mmol scale)	10	61 (71 brsm)	13:1

[a] Isolated yields after purification by column chromatography.
[b] Diastereomeric ratios in the crude mixture before purification.

Table S2. Cycloaddition of diester **12a** under various irradiation conditions.

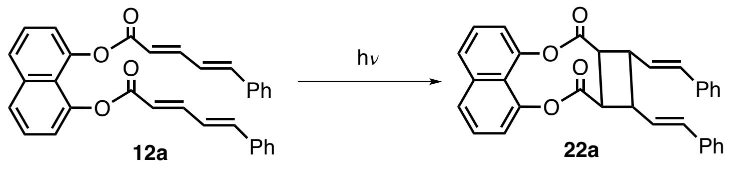

entry	light source	sample	time (h)	conversion (%)	dr (crude)
1	UV-B	powder	16	25[a]	20:1
2	daylight	powder	216	9[a]	-
3	daylight	solution ($CDCl_3$)	168	57[a]	20:1
4	UV-B	solution ($CHCl_3$)	2	58[b]	6:1

[a] The % conversion of **12a** to **22a** was determined by ^{1}H-NMR analysis of the crude mixture before purification. [b] Isolated yield after purification by column chromatography.

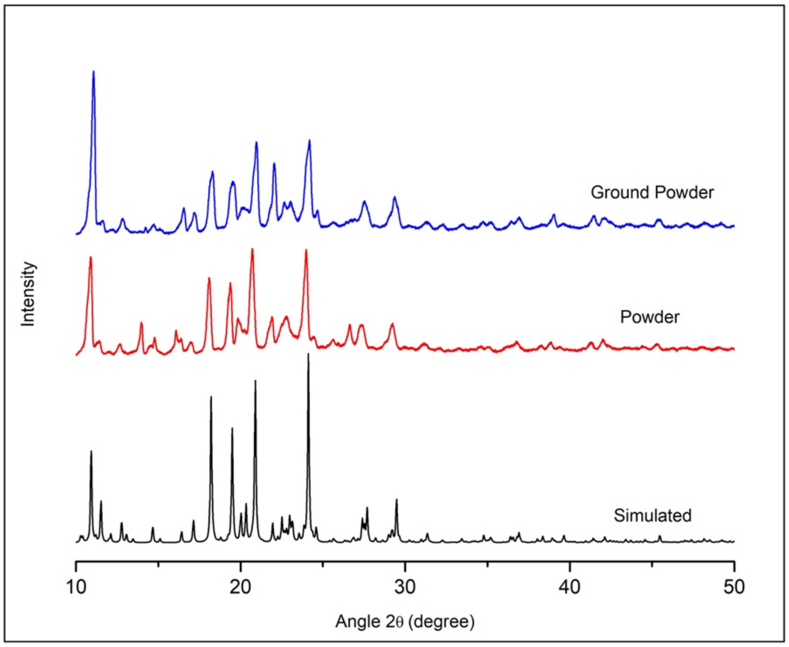

Figure S2. Powder XRD patterns of different diester **12a** samples.

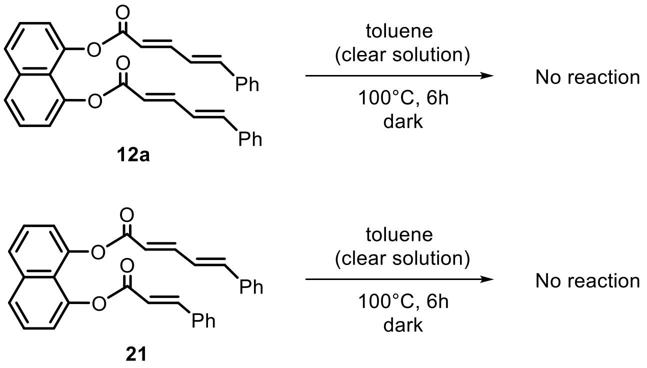

Scheme S1. Control Experiments on the thermal cycloadditions of diesters **12a** and **21**.

Table S3. Investigation of the Cope rearrangement of divinyl cyclobutane **22a**.

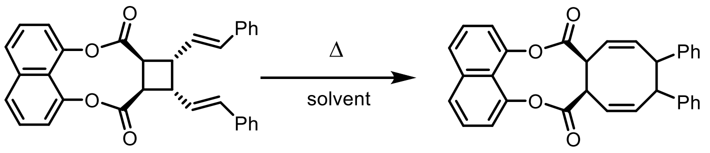

22a

entry	temperature (°C)	solvent	time (h)	result
**1**	60	$CDCl_3$	4	no conversion
**2**	100	toluene	4	no conversion
**3**	130	chlorobenzene	4	no conversion
**4**	130 (sealed tube)	benzene	4	no conversion
**5**	160 (sealed tube)	chlorobenzene	8	decomposition
**6**	160 (sealed tube)	chlorobenzene	4	**12a** obtained

Table S4. Investigation of the Cope rearrangement of divinyl cyclobutane **13a**.

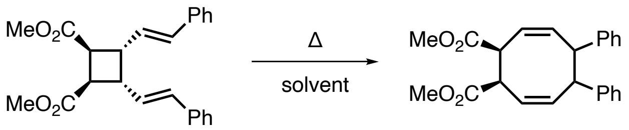

13a

entry	temperature (°C)	solvent	time (h)	result
**1**	130	benzene	4	No conversion
**2**	160	chlorobenzene	4	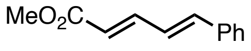 obtained

X-ray data collection and structure refinement of 12a, 16a, and 22a

Single crystal data for **12a**, **16a** and **22a** were acquired using a Bruker APEX-II CCD with MoKα (λ = 0.71073) radiation. Data indexing, integration, and absorption correction using APEX suite [APEX2, version 2014.11-0, Bruker (2014), Bruker AXS Inc., Madison, WI] were done. Crystal structures were solved using SHELXT [Sheldrick, G. M. Acta Crystallogr. Sect. A Found. Crystallogr. 2015, 71 (1), 3-8], and then refined using the SHELXL [Sheldrick, G. M. Acta Crystallogr. Sect. A Found. Crystallogr. 2015, 71 (1), 3-8] in Olex2 program package [O.V. Dolomanov, L.J. Bourhis, R.J. Gildea, J.A.K. Howard, H. Puschmann, J. Appl. Cryst., 2009, 42, 339-341]. All crystallographic data and refinement parameters are listed in **Table S5.** Additional crystallographic data with CCDC reference numbers 2354346 (compound **16a**), 2354337 (compound **12a**) and 2354338 (compound **22a**) have been deposited within the Cambridge Crystallographic Data Center via www.ccdc.cam.ac.uk/deposit.

Table S5. Crystal data and structure refinement for compounds **12a**, **16a** and **22a**.

Compound	16a	12a	22a
**CDDC**	2354346	2354337	2354338
**Empirical formula**	$C_{11}H_{10}O_2$	$C_{32}H_{24}O_4$	$C_{32}H_{24}O_4$
**Formula weight**	174.19	472.51	472.51
**Temperature/K**	298	298	298
**Crystal system**	Orthorhombic	Triclinic	Monoclinic
**Space group**	Pbca	P-1	$P2_1/n$
**a/Å**	10.484(2)	8.7378(7)	15.2852(12)
**b/Å**	9.478(2)	9.7867(8)	10.2220(8)
**c/Å**	37.488(8)	16.6929(15)	16.2643(13)
**α/°**	90	76.345(2)	90
**β/°**	90	87.788(2)	95.105(2)
**γ/°**	90	64.867(2)	90
**Volume/Å^3**	3724.9(14)	1252.72(18)	2531.1(3)
**Z**	16	2	4
**ρ_{calc}g/cm^3**	1.242	1.253	1.240
**μ/mm^{-1}**	0.085	0.082	0.081
**F(000)**	1472.0	496.0	992.0
**Crystal size/mm^3**	0.299 × 0.167 × 0.096	0.359 × 0.2 × 0.185	0.32 × 0.292 × 0.12
**2Θ range for data collection/°**	5.83 to 50.7	2.516 to 49.992	6.418 to 50.7
**Index ranges**	$-12 \leq h \leq 12, -11 \leq k \leq 10, -45 \leq l \leq 38$	$-10 \leq h \leq 10, -11 \leq k \leq 11, -19 \leq l \leq 19$	$-18 \leq h \leq 18, -12 \leq k \leq 12, -19 \leq l \leq 19$
**Reflections collected**	16375	11377	24221
**Independent reflections**	3401 [R_{int} = 0.0682, R_{sigma} = 0.0604]	4408 [R_{int} = 0.0258, R_{sigma} = 0.0340]	4636 [R_{int} = 0.0600, R_{sigma} = 0.0451]
**Data/restraints/parameters**	3401/1/240	4408/0/326	4636/0/325
**Goodness-of-fit on F^2**	1.010	1.060	1.016
**Final R indexes [I>=2σ (I)]**	R_1 = 0.0499, wR_2 = 0.1089	R_1 = 0.0460, wR_2 = 0.1148	R_1 = 0.0440, wR_2 = 0.0990
**Final R indexes [all data]**	R_1 = 0.1113, wR_2 = 0.1374	R_1 = 0.0776, wR_2 = 0.1274	R_1 = 0.0780, wR_2 = 0.1173
**Largest diff. peak/hole / e Å^{-3}**	0.14/-0.15	0.22/-0.18	0.12/-0.18

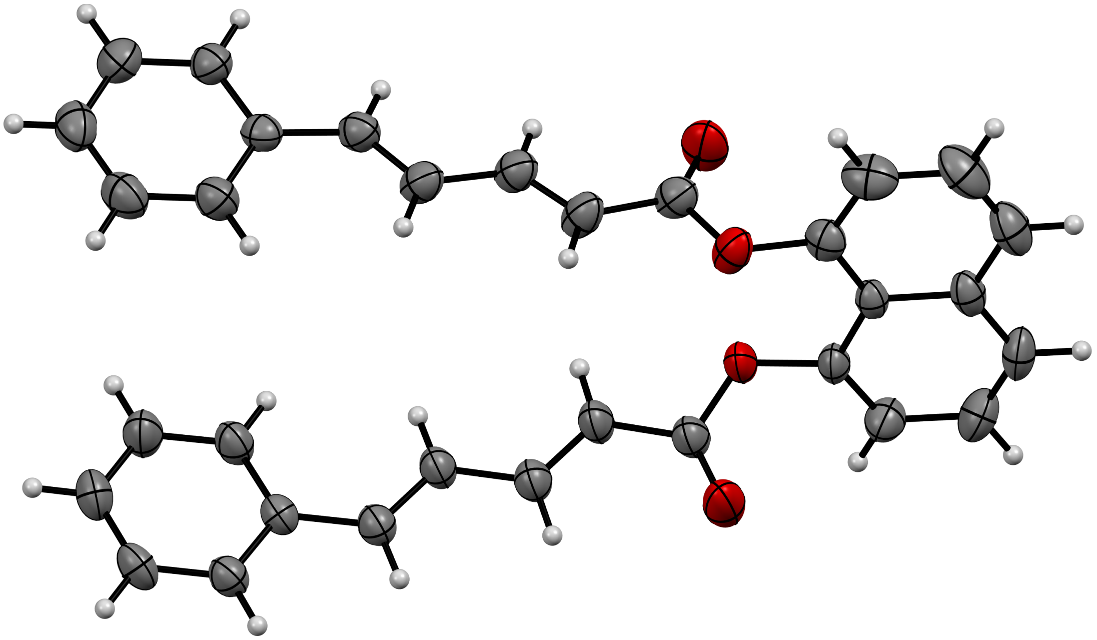

Figure S3. X-ray crystal structure of diester **12a** with 30% ellipsoids. The grey, red, and white coloured atoms represent C, O, and H, respectively.

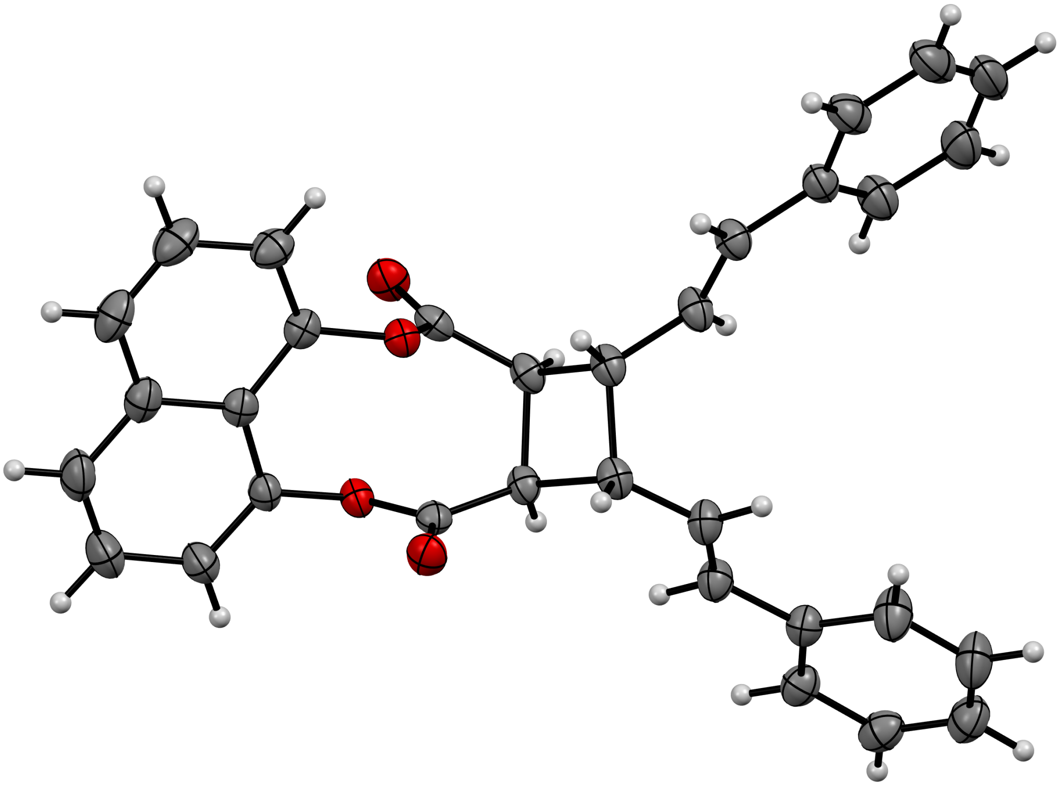

Figure S4. X-ray crystal structure of cycloadduct **22a** with 30% ellipsoids. The grey, red, and white coloured atoms represent C, O, and H, respectively.

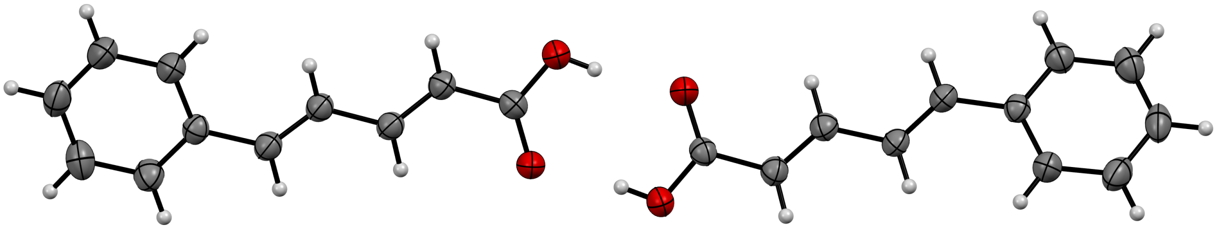

Figure S5. X-ray crystal structure of (2*E*,4*E*)-5-phenylpenta-2,4-dienoic acid (**16a**) with 30% ellipsoids. The grey, red, and white coloured atoms represent C, O, and H, respectively.

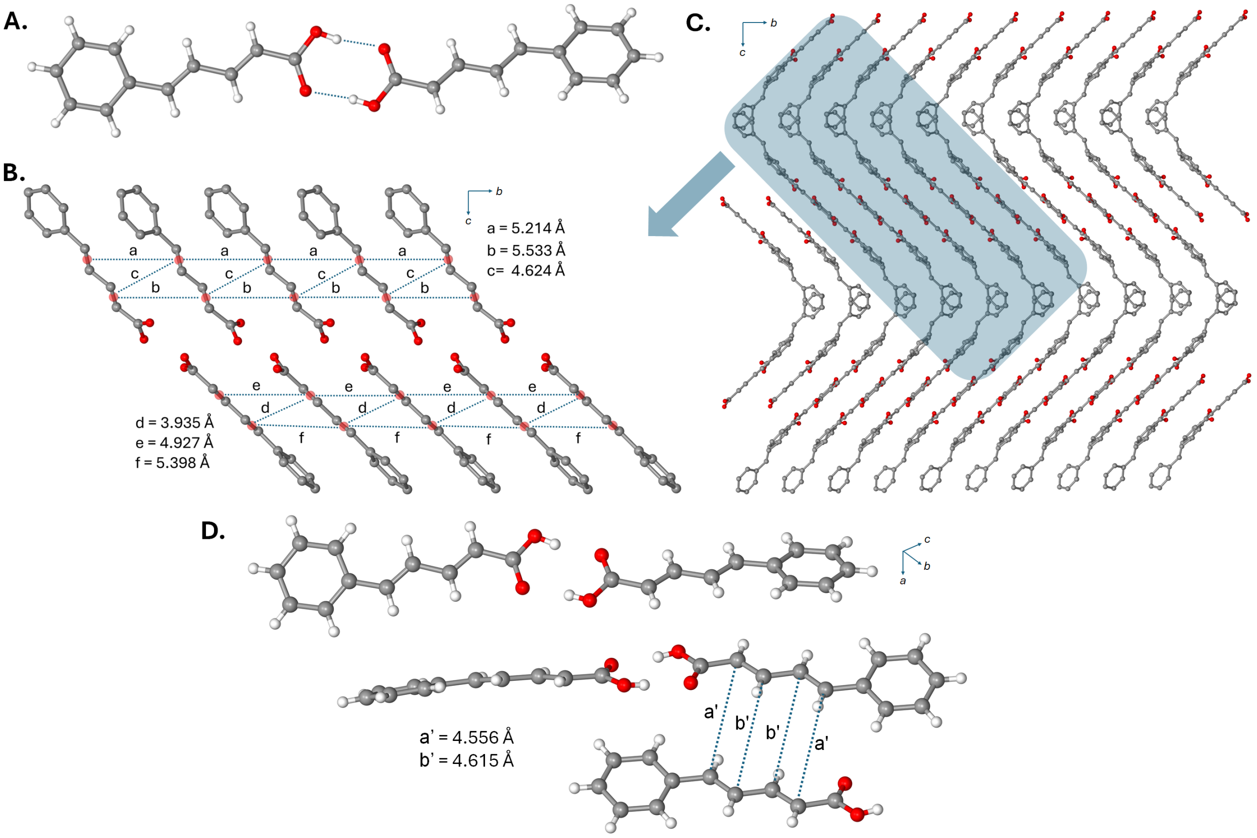

Figure S6. (A) Crystal structure of (2*E*,4*E*)-5-phenylpenta-2,4-dienoic acid (**16a**). **(B)** A perspective view showing the distances between the double bonds in **(C)** herringbone packing. **(D)** A perspective picture showing the distance between C···C atoms.

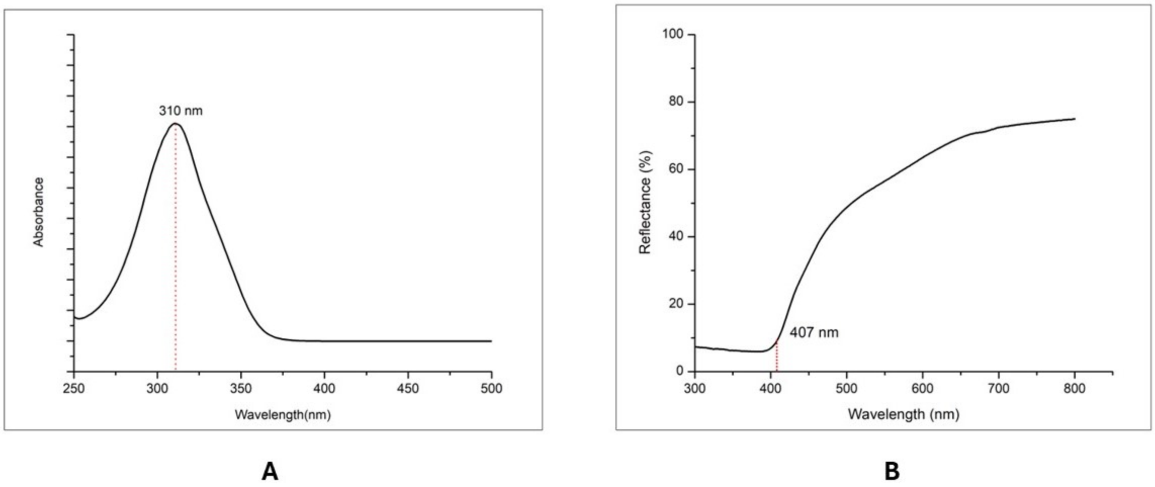

Figure S7. a) UV-Vis spectrum of compound **12a** in CH_2Cl_2; **b)** UV-Vis diffused reflectance spectrum of compound **12a**.

^{1}H-, $^{13}C\{^{1}H\}$- and $^{19}F\{^{1}H\}$-NMR spectra:

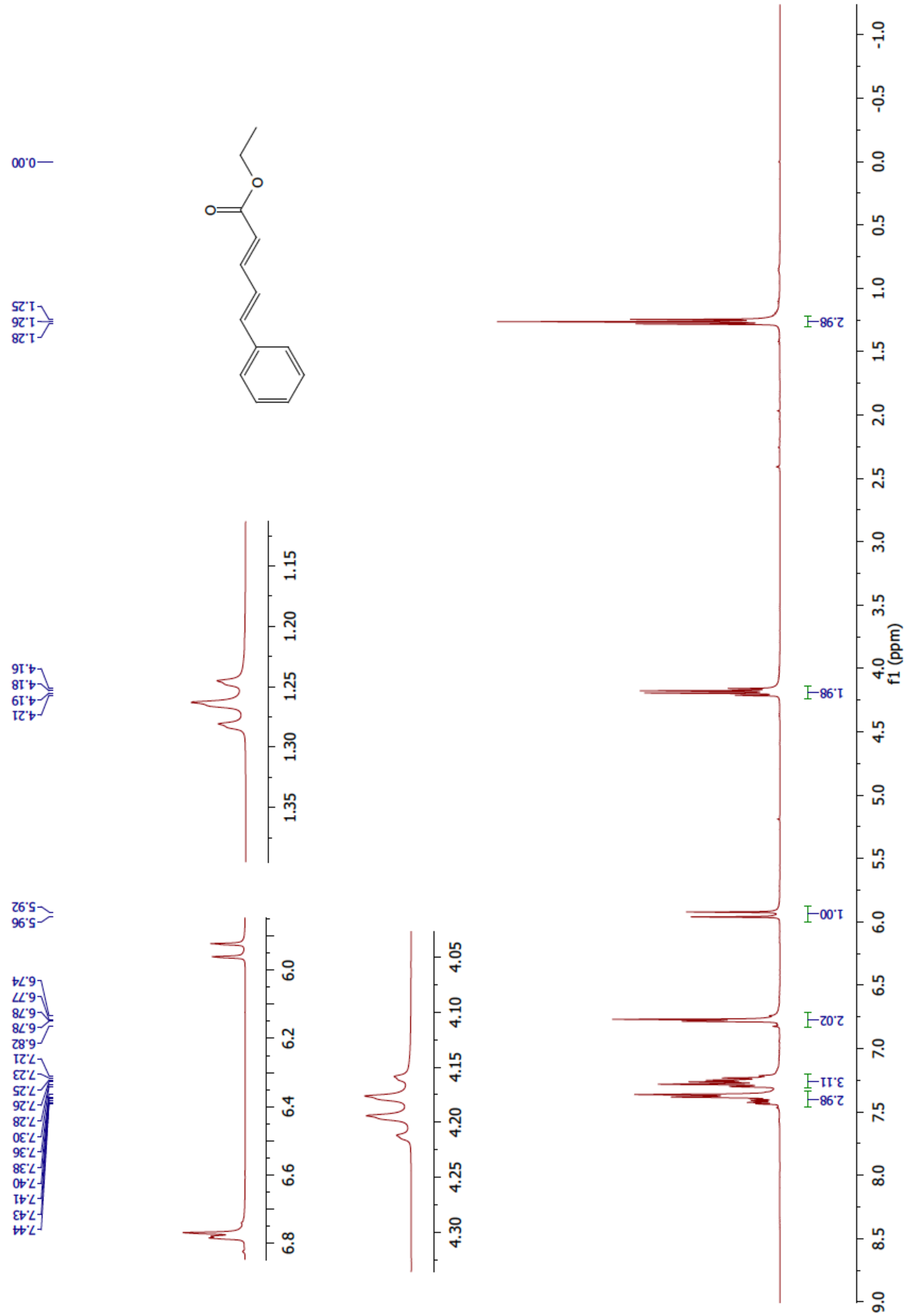

Figure S8. ^{1}H-NMR spectrum of **15a** in $CDCl_3$ (400 MHz).

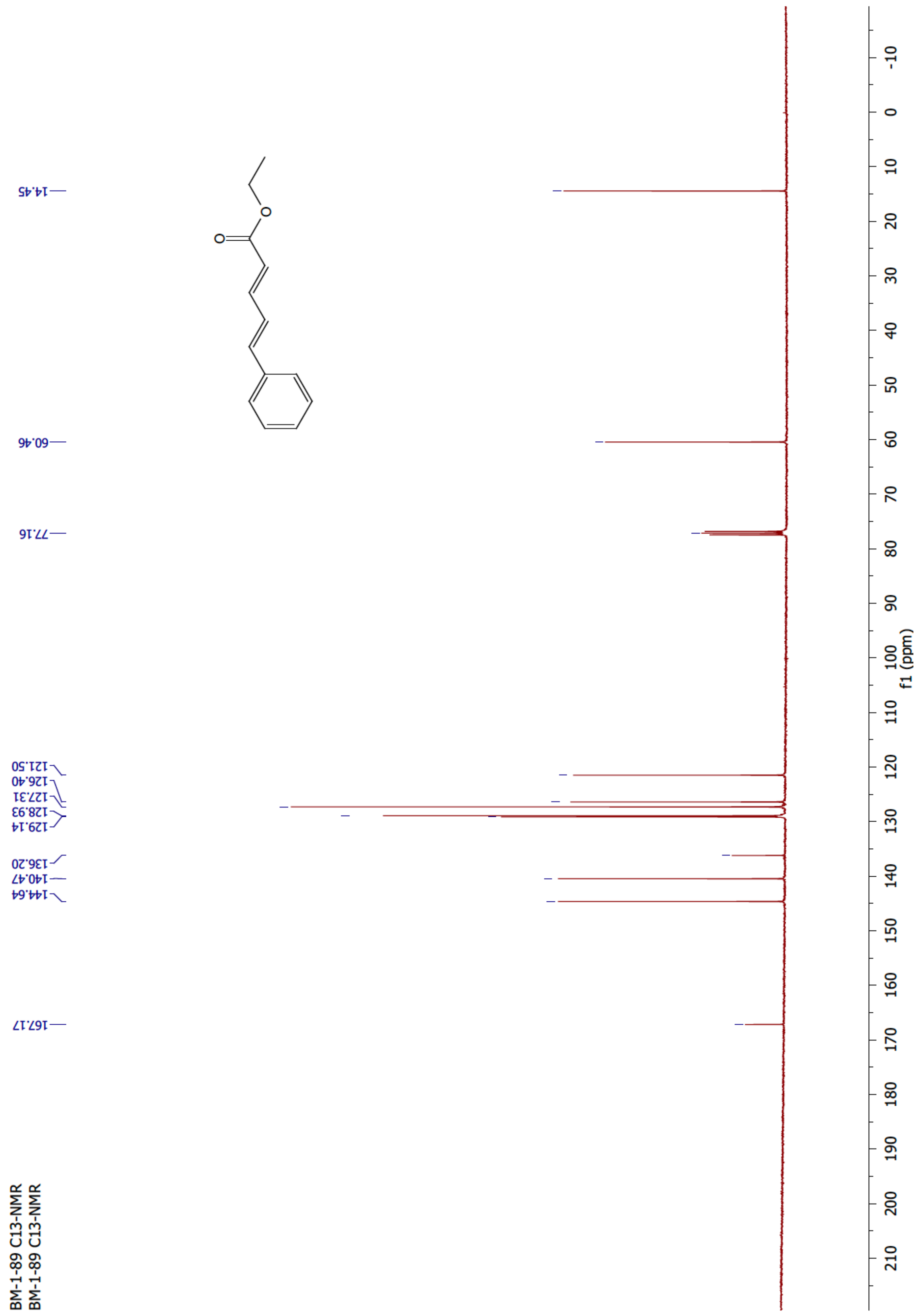

Figure S9. $^{13}C\{^{1}H\}$-NMR spectrum of **15a** in $CDCl_3$ (100 MHz).

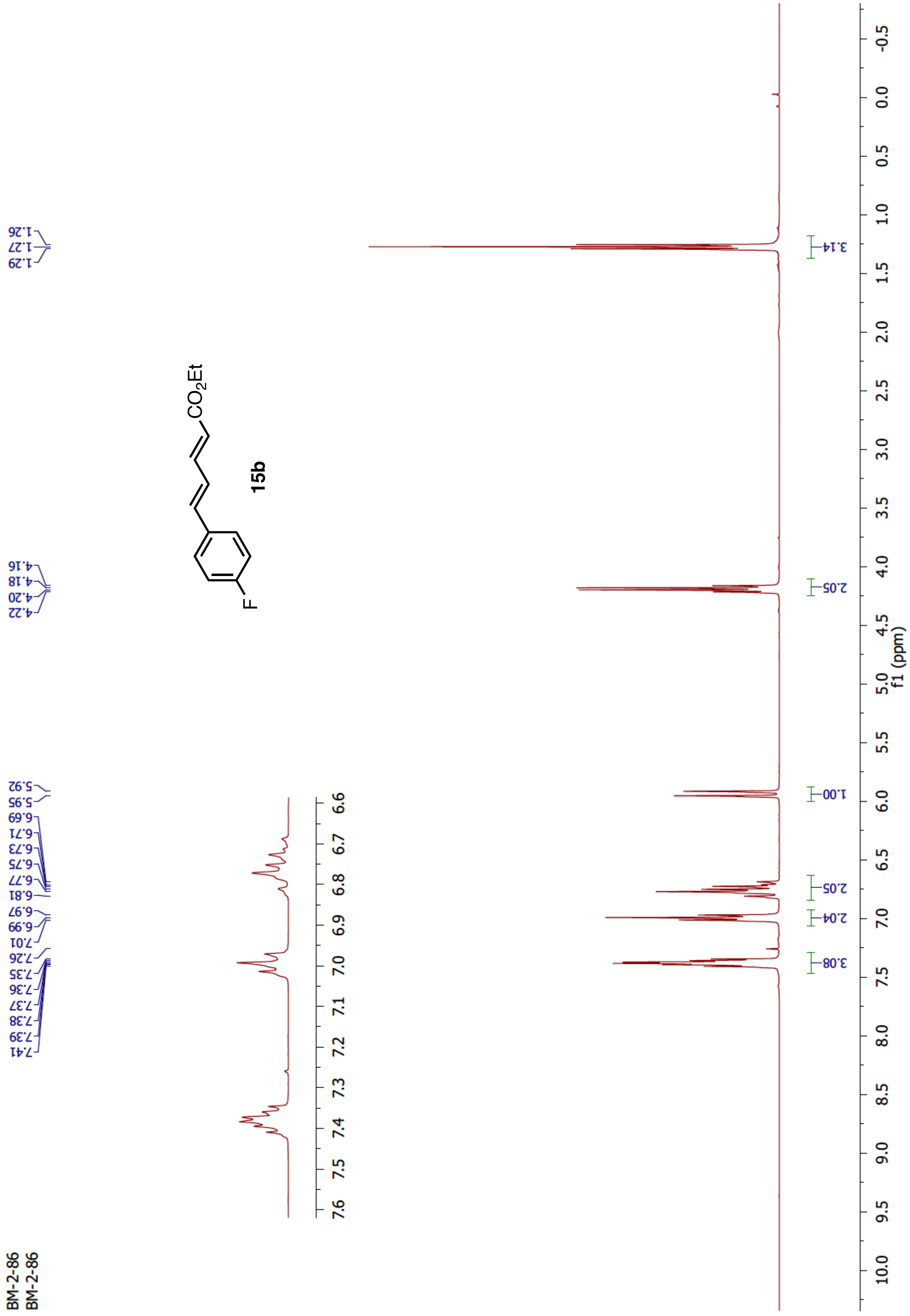

Figure S10. ^{1}H-NMR spectrum of **15b** in $CDCl_3$ (400 MHz).

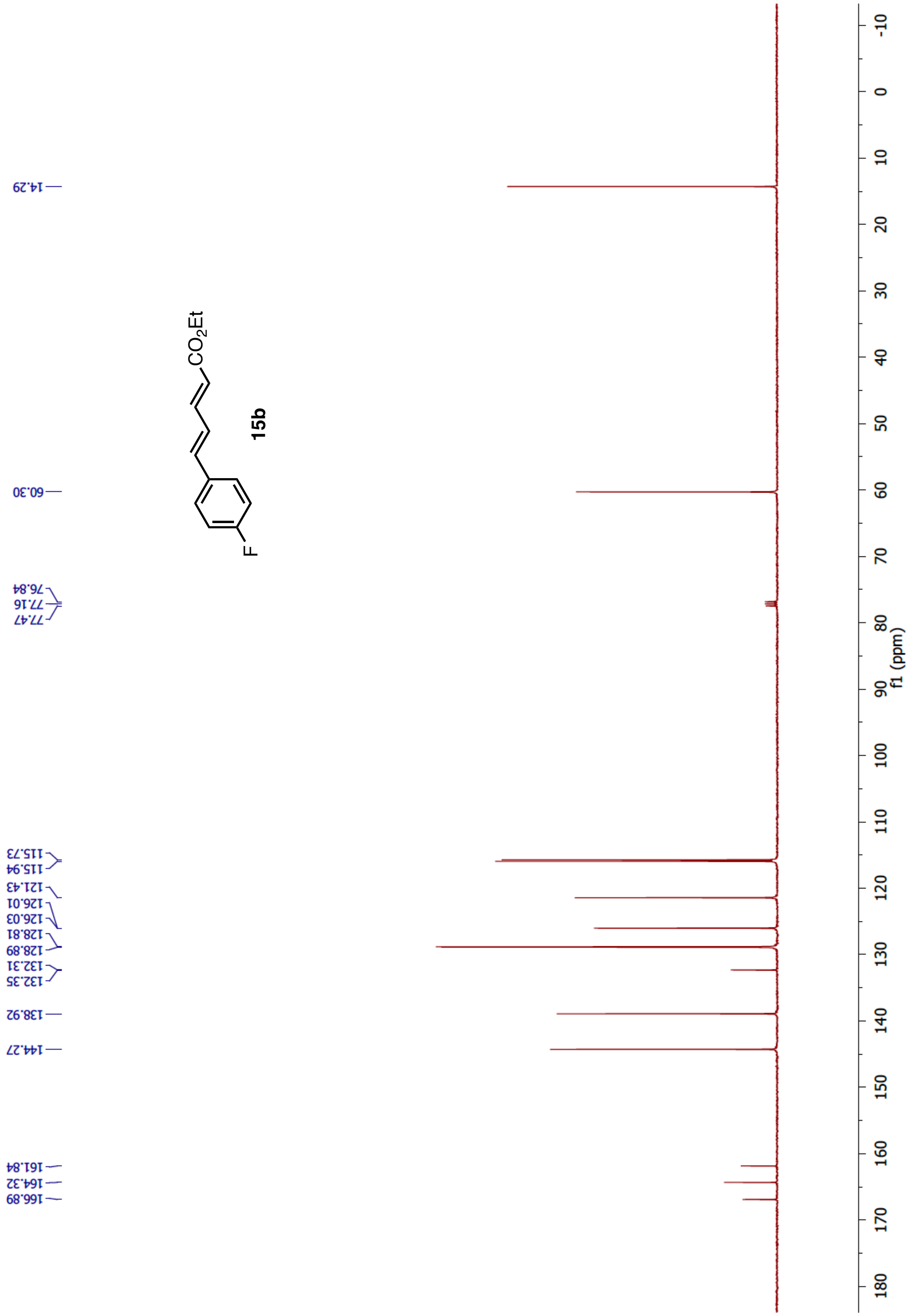

Figure S11. $^{13}C\{^1H\}$-NMR spectrum of **15b** in $CDCl_3$ (100 MHz).

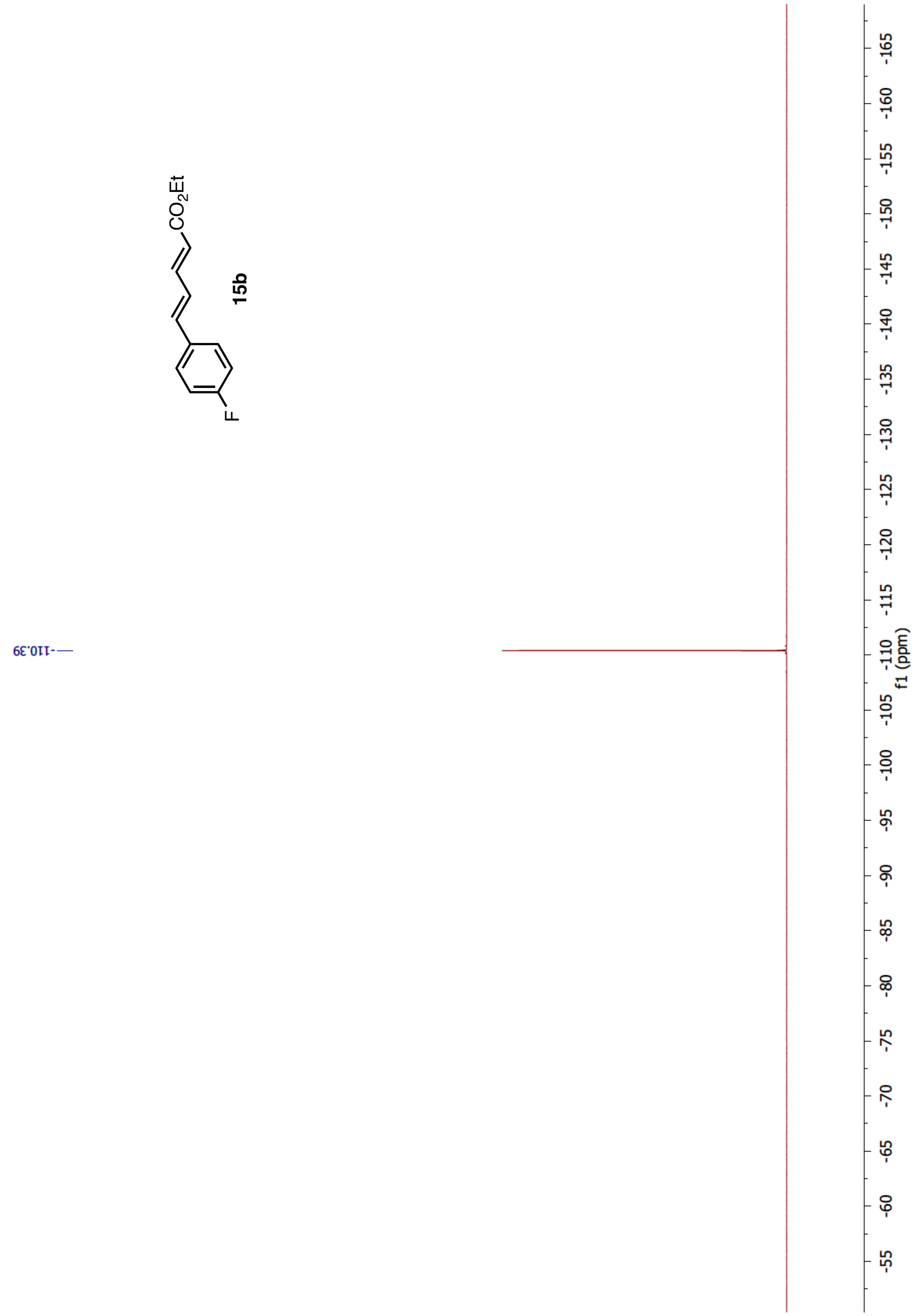

Figure S12. $^{19}F\{^{1}H\}$-NMR spectrum of **15b** in $CDCl_3$ (376 MHz).

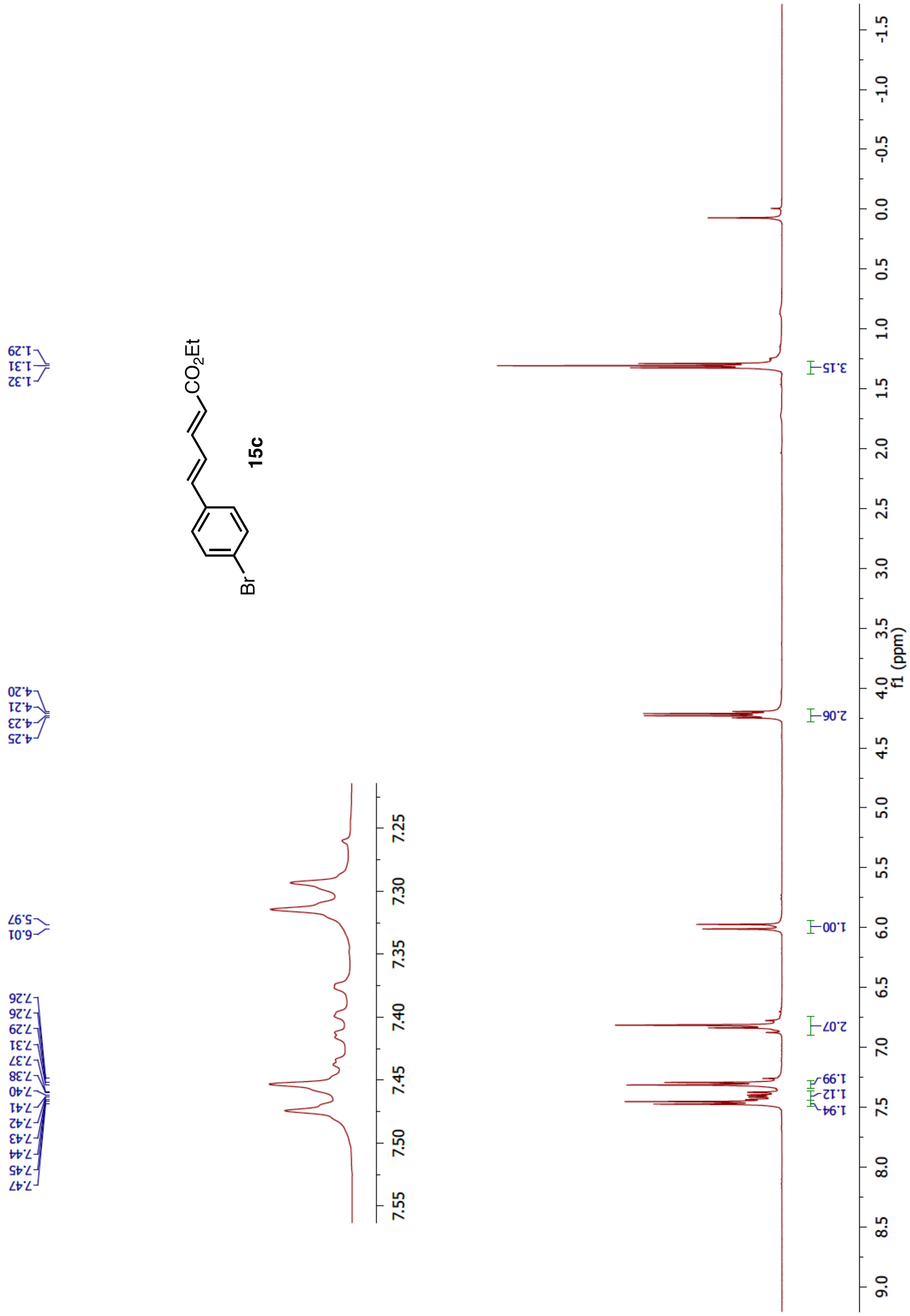

Figure S13. ^{1}H-NMR spectrum of **15c** in $CDCl_3$ (400 MHz).

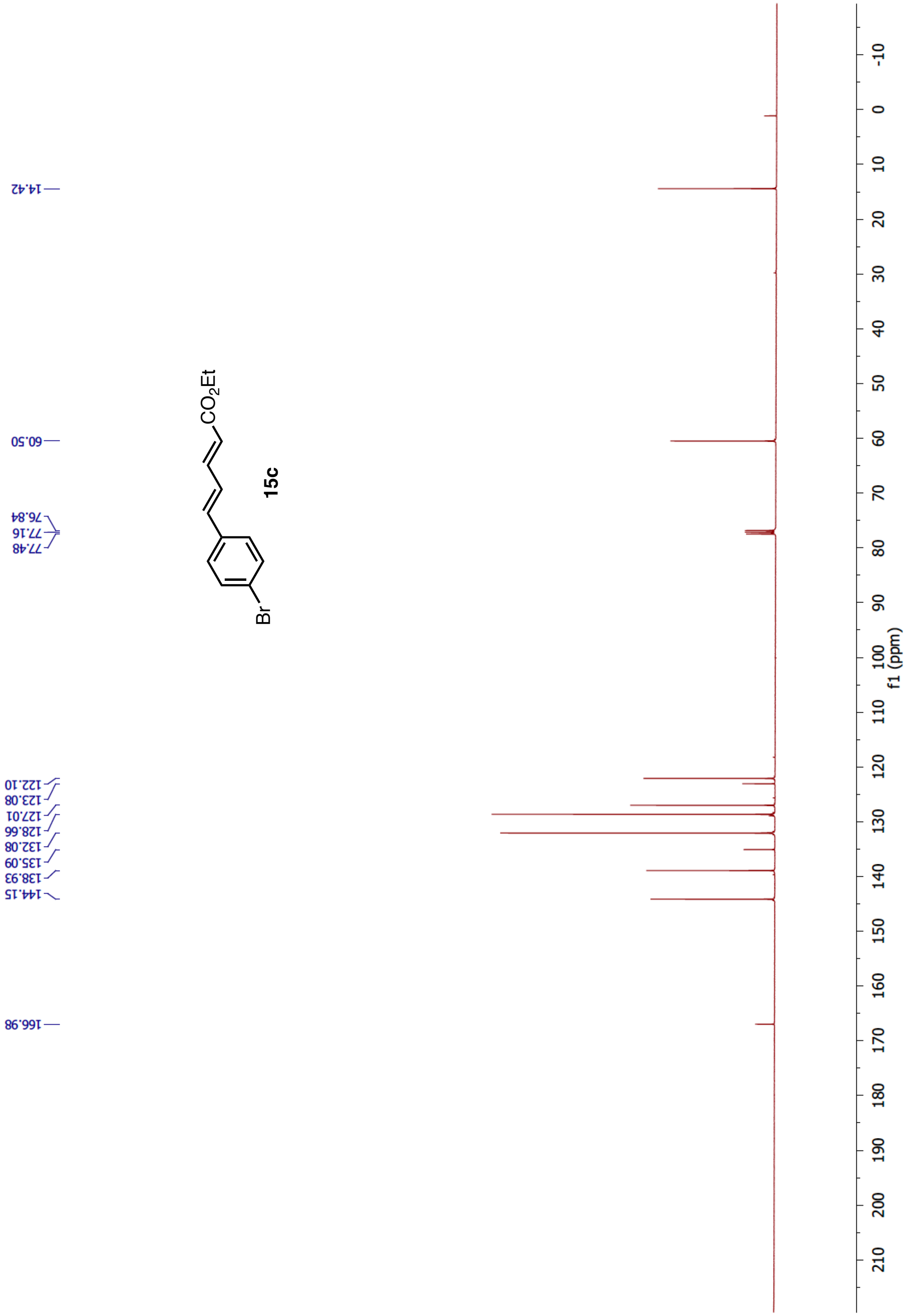

Figure S14. $^{13}C\{^{1}H\}$-NMR spectrum of **15c** in $CDCl_3$ (100 MHz).

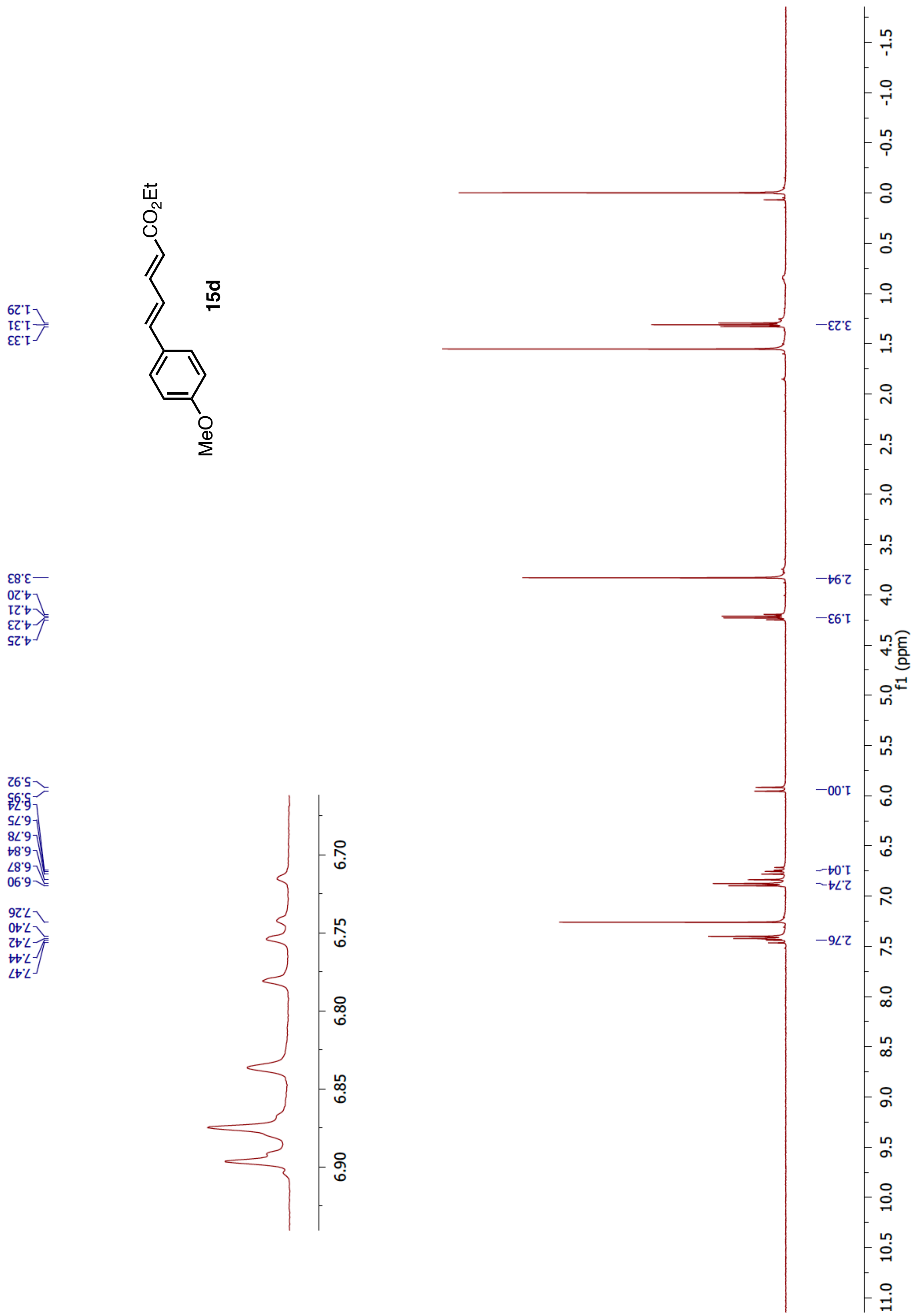

Figure S15. ^{1}H-NMR spectrum of **15d** in $CDCl_3$ (400 MHz).

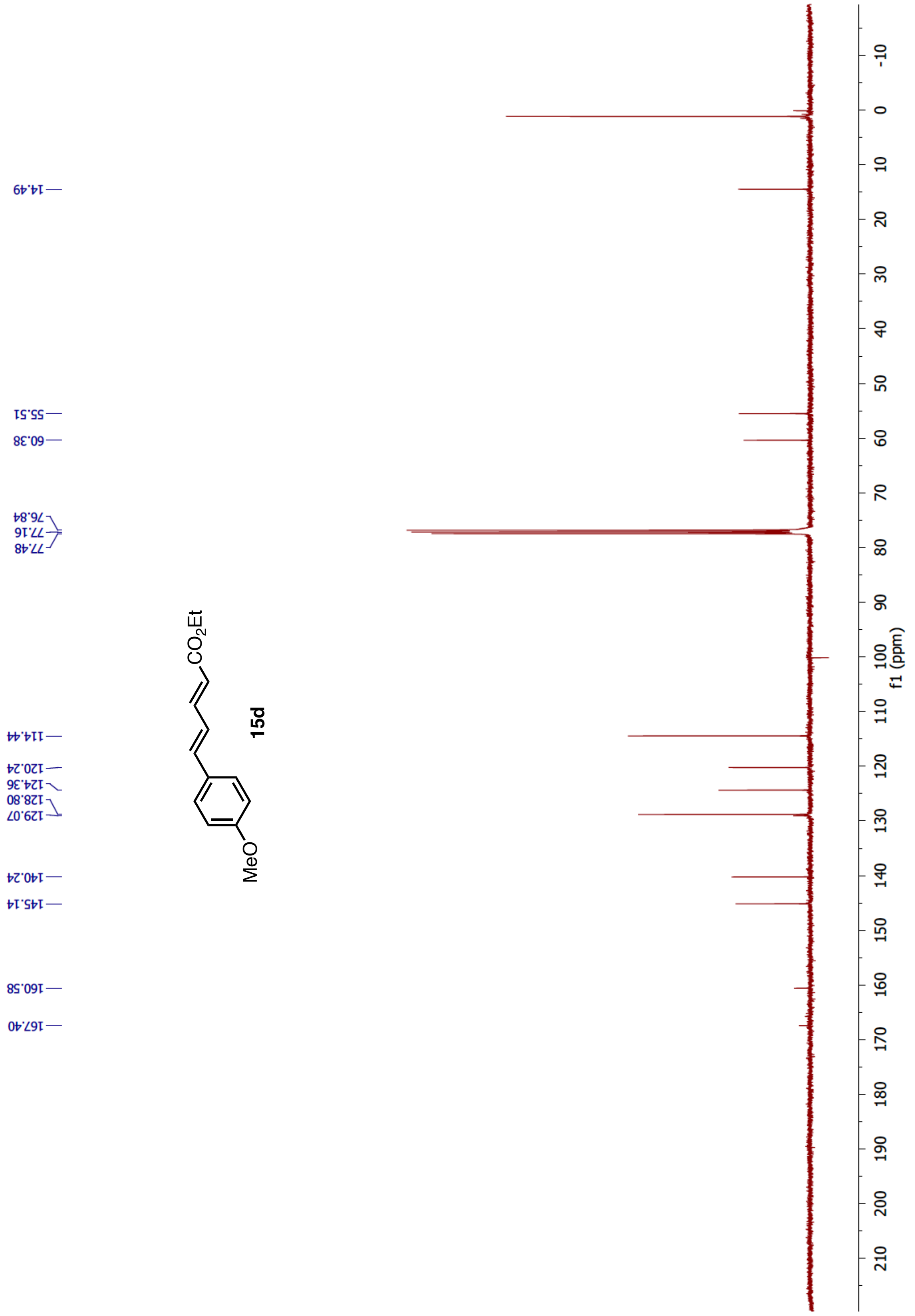

Figure S16. $^{13}C\{^{1}H\}$-NMR spectrum of **15d** in $CDCl_3$ (100 MHz).

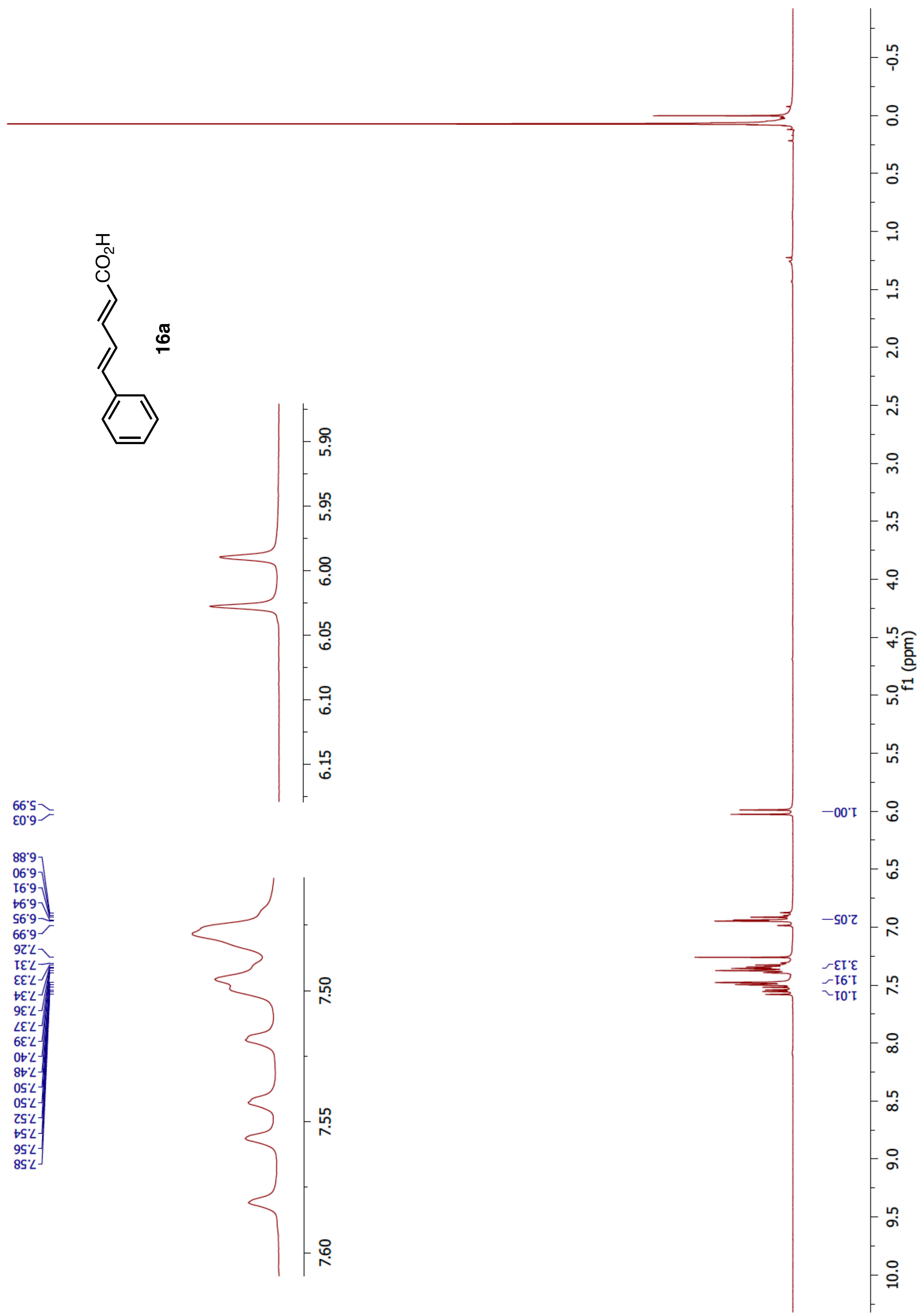

Figure S17. ^{1}H-NMR spectrum of **16a** in $CDCl_3$ (400 MHz).

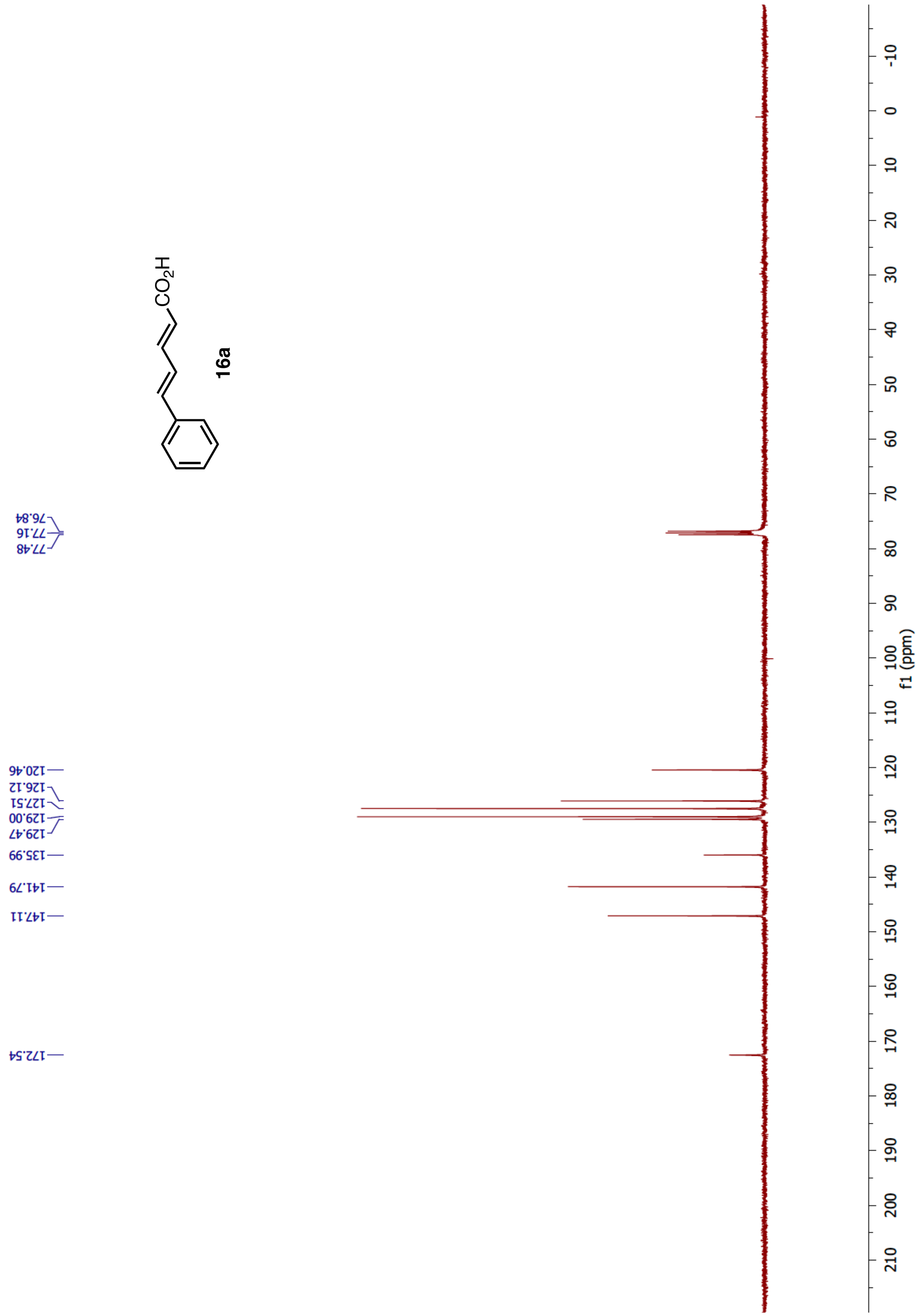

Figure S18. $^{13}C\{^{1}H\}$-NMR spectrum of **16a** in $CDCl_3$ (100 MHz).

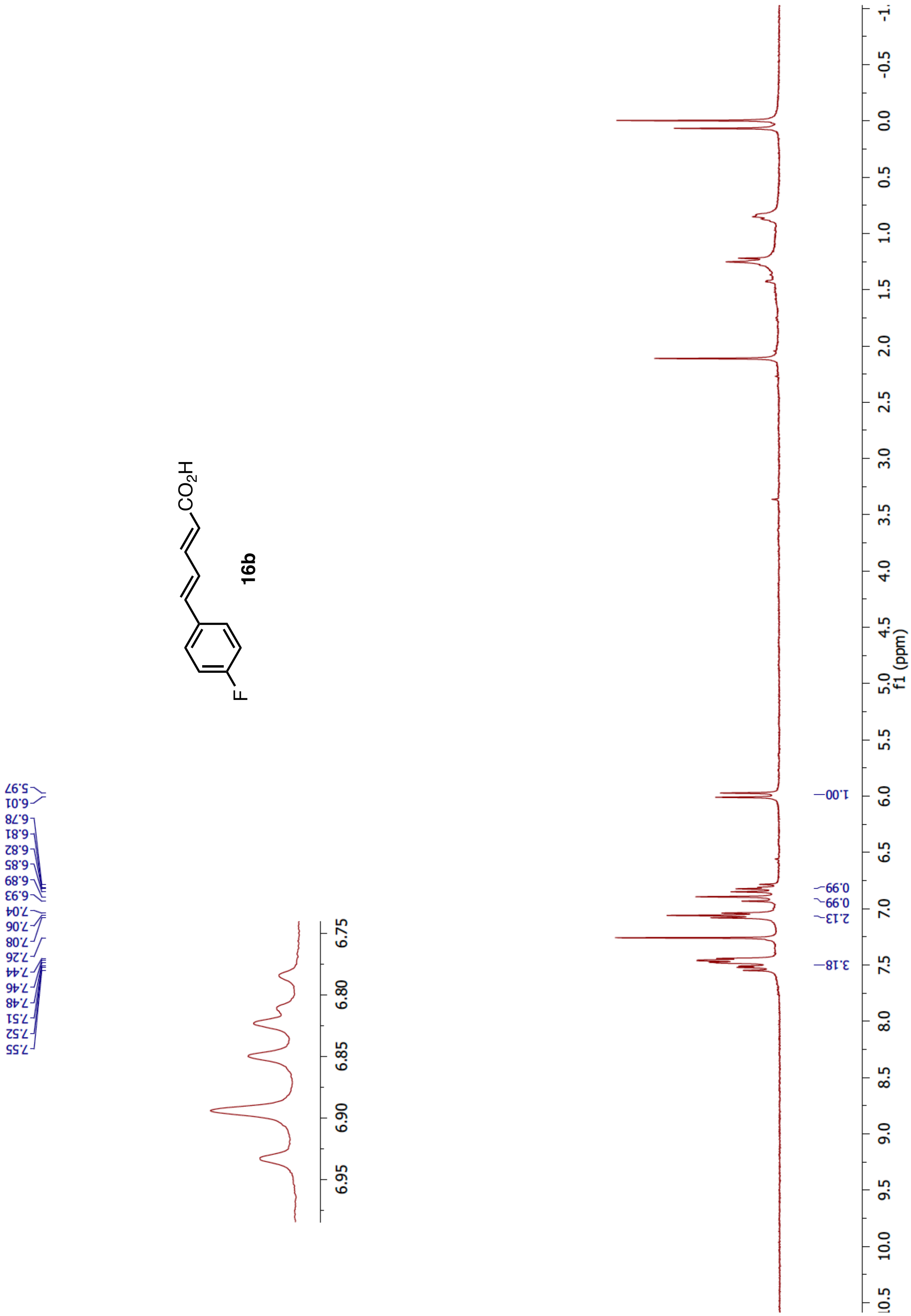

Figure S19. ^{1}H-NMR spectrum of **16b** in $CDCl_3$ (400 MHz).

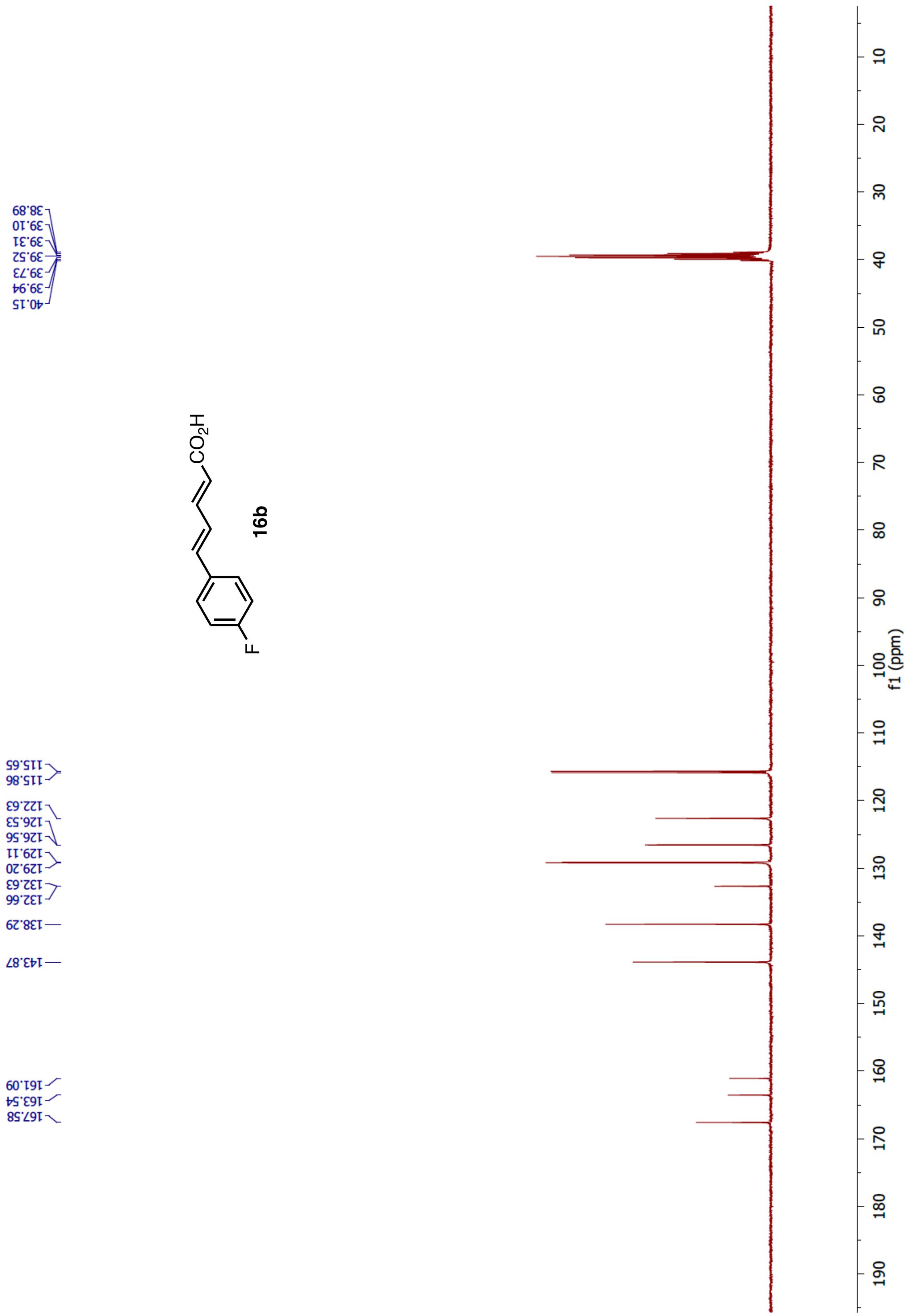

Figure S20. $^{13}C\{^1H\}$-NMR spectrum of **16b** in DMSO-d_6 (100 MHz).

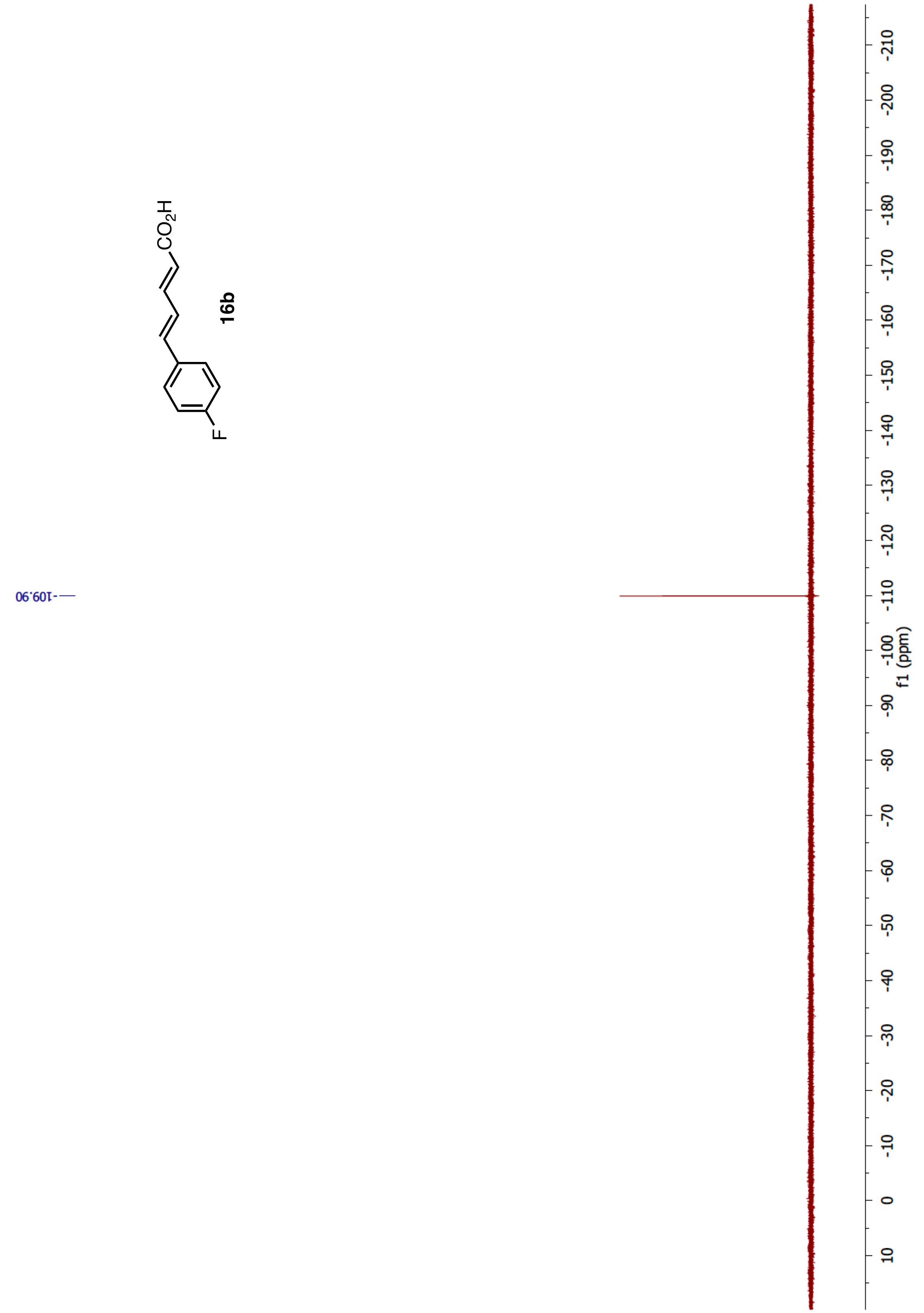

Figure S21. $^{19}F\{^{1}H\}$-NMR spectrum of **16b** in $CDCl_3$ (376 MHz).

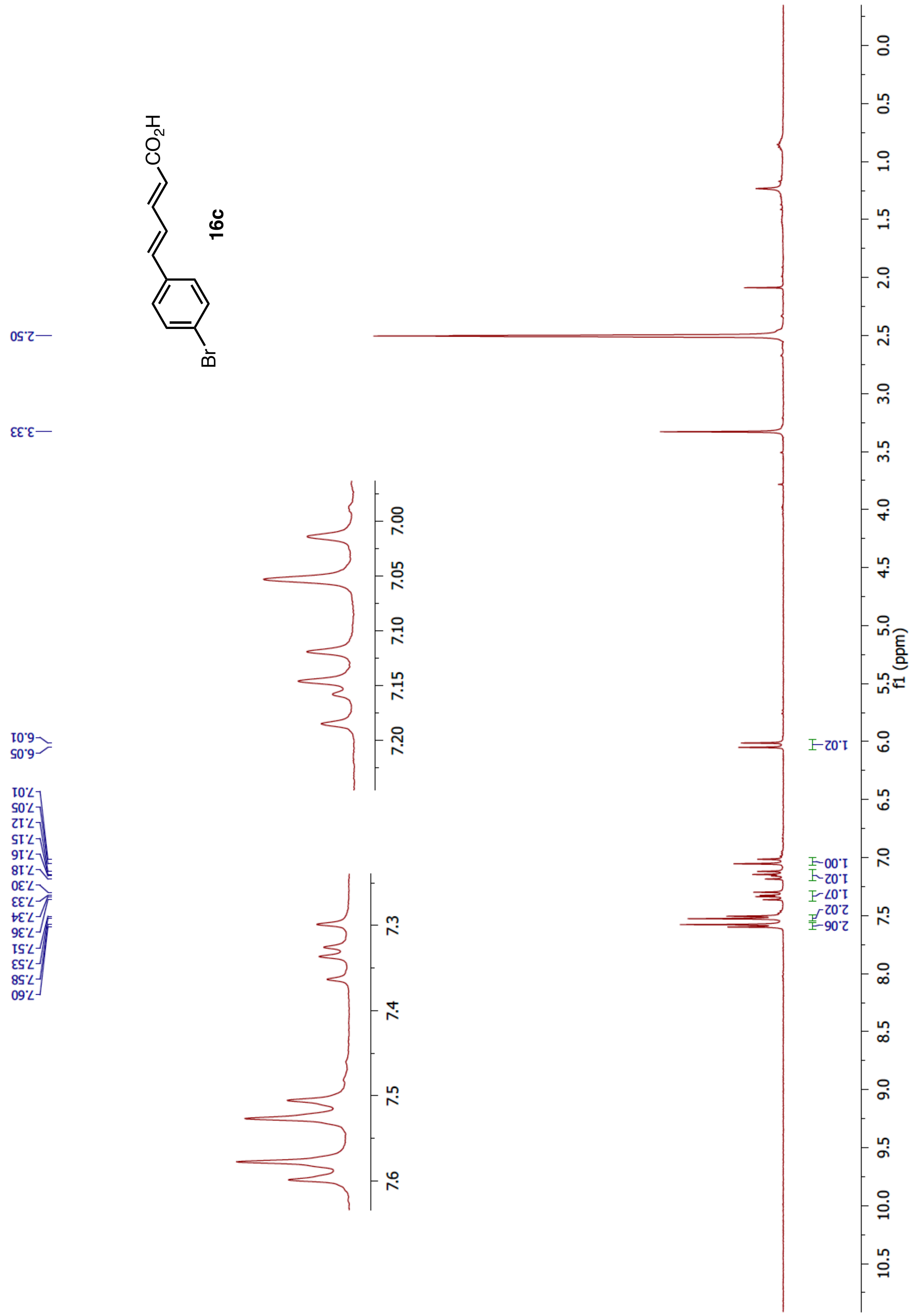

Figure S22. ^{1}H-NMR spectrum of **16c** in DMSO-d_6 (400 MHz).

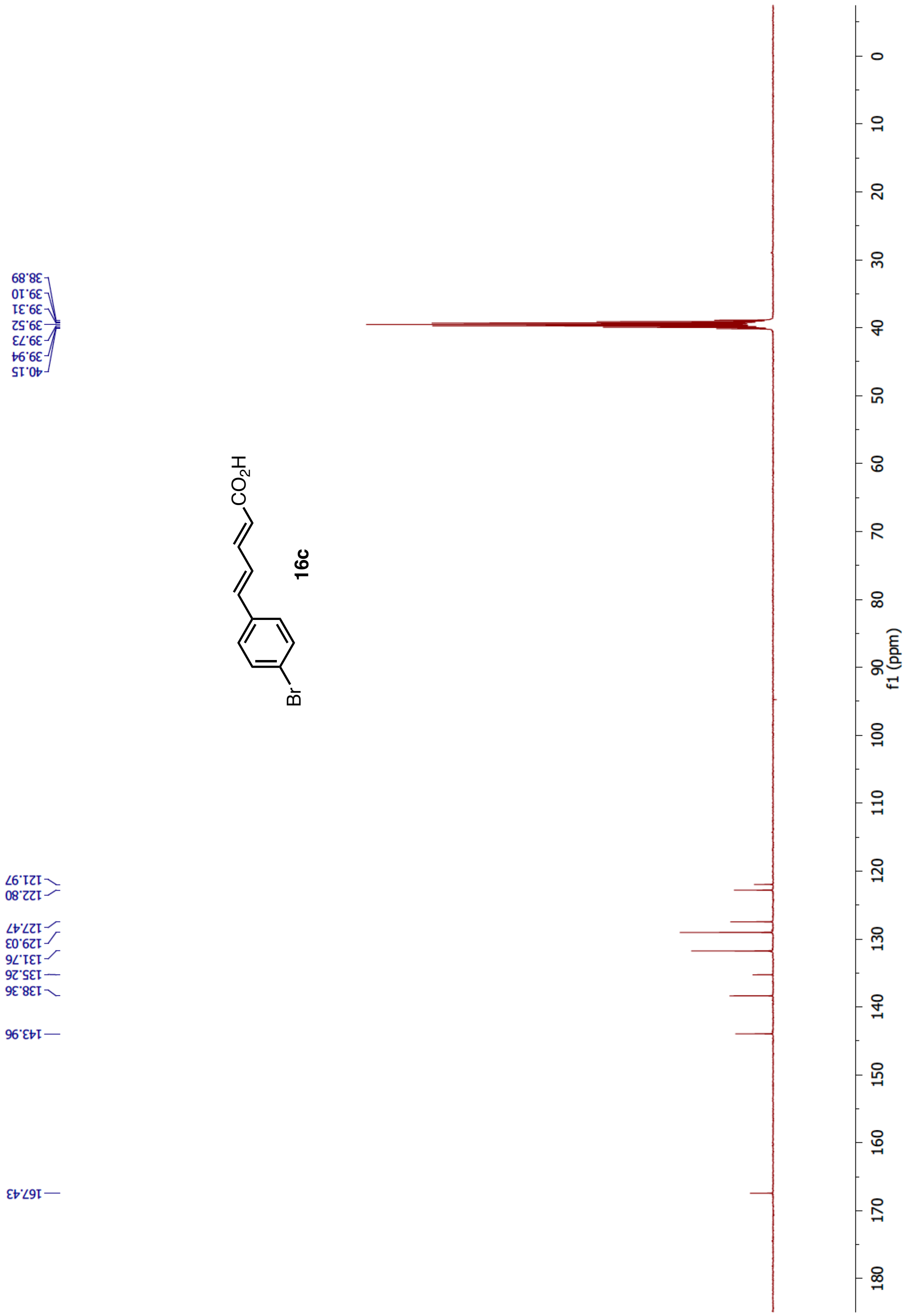

Figure S23. $^{13}C\{^{1}H\}$-NMR spectrum of **16c** in DMSO-d_6 (100 MHz).

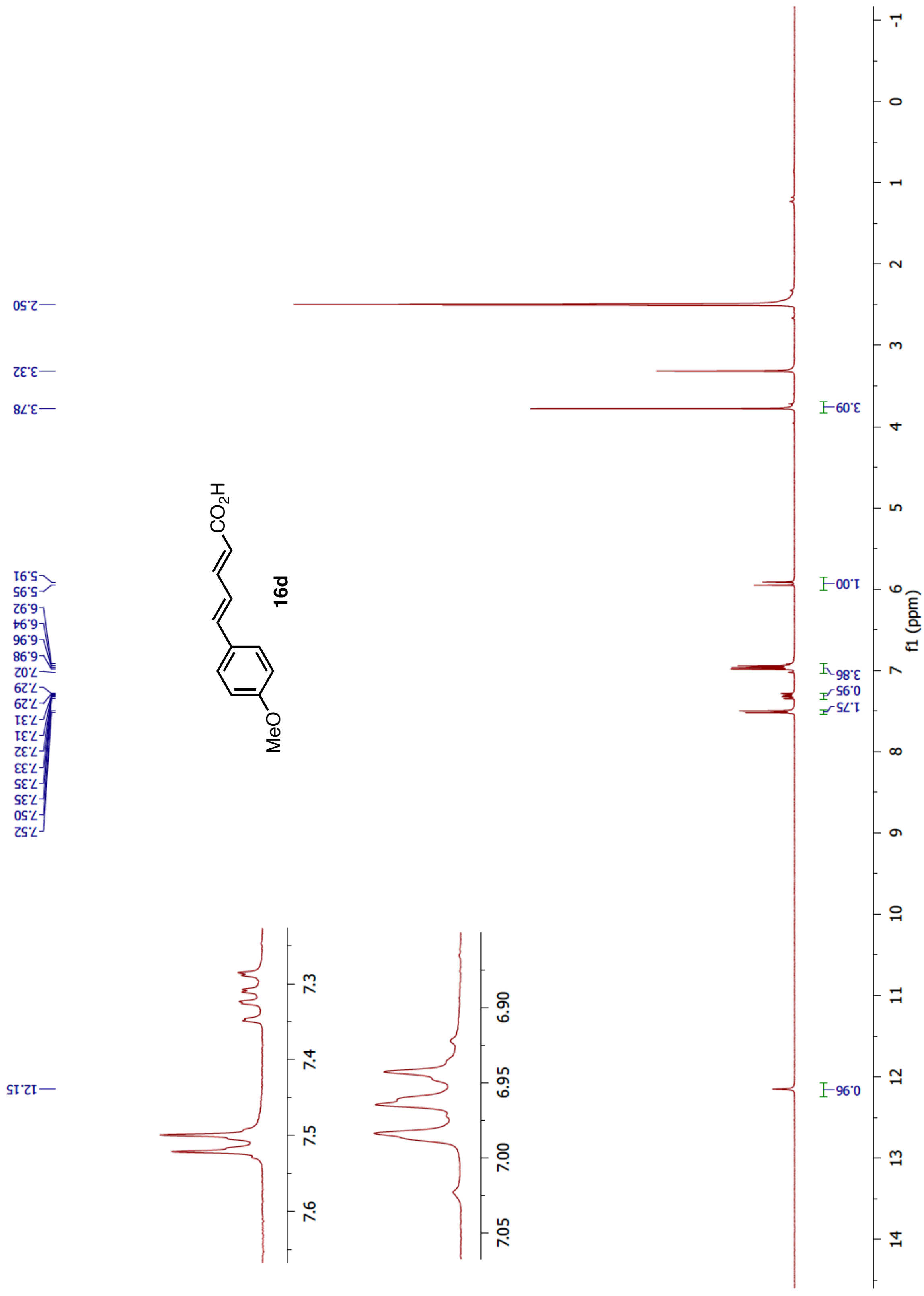

Figure S24. ^{1}H-NMR spectrum of **16d** in DMSO-d_6 (400 MHz).

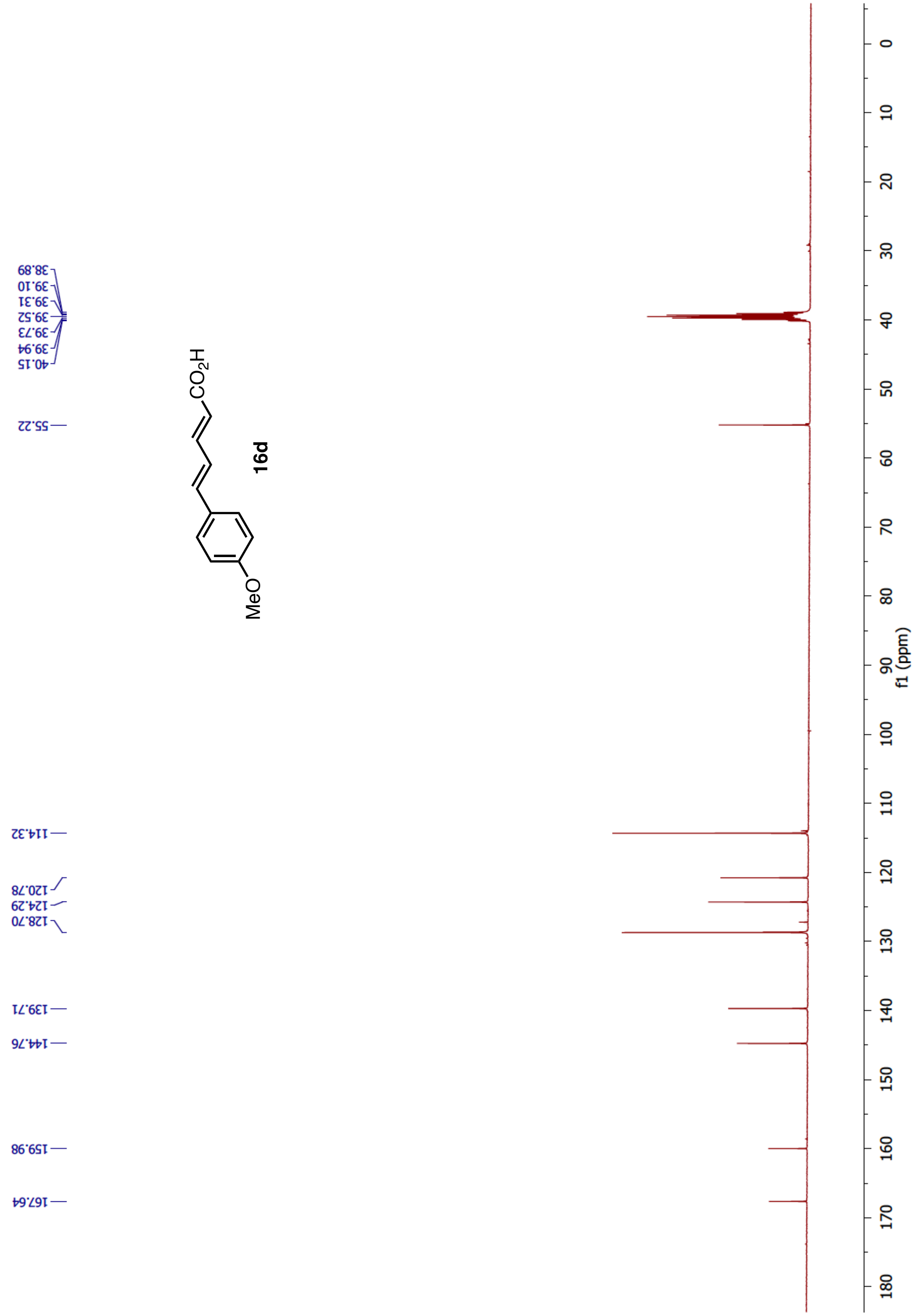

Figure S25. $^{13}C\{^1H\}$-NMR spectrum of **16d** in DMSO-d_6 (100 MHz).

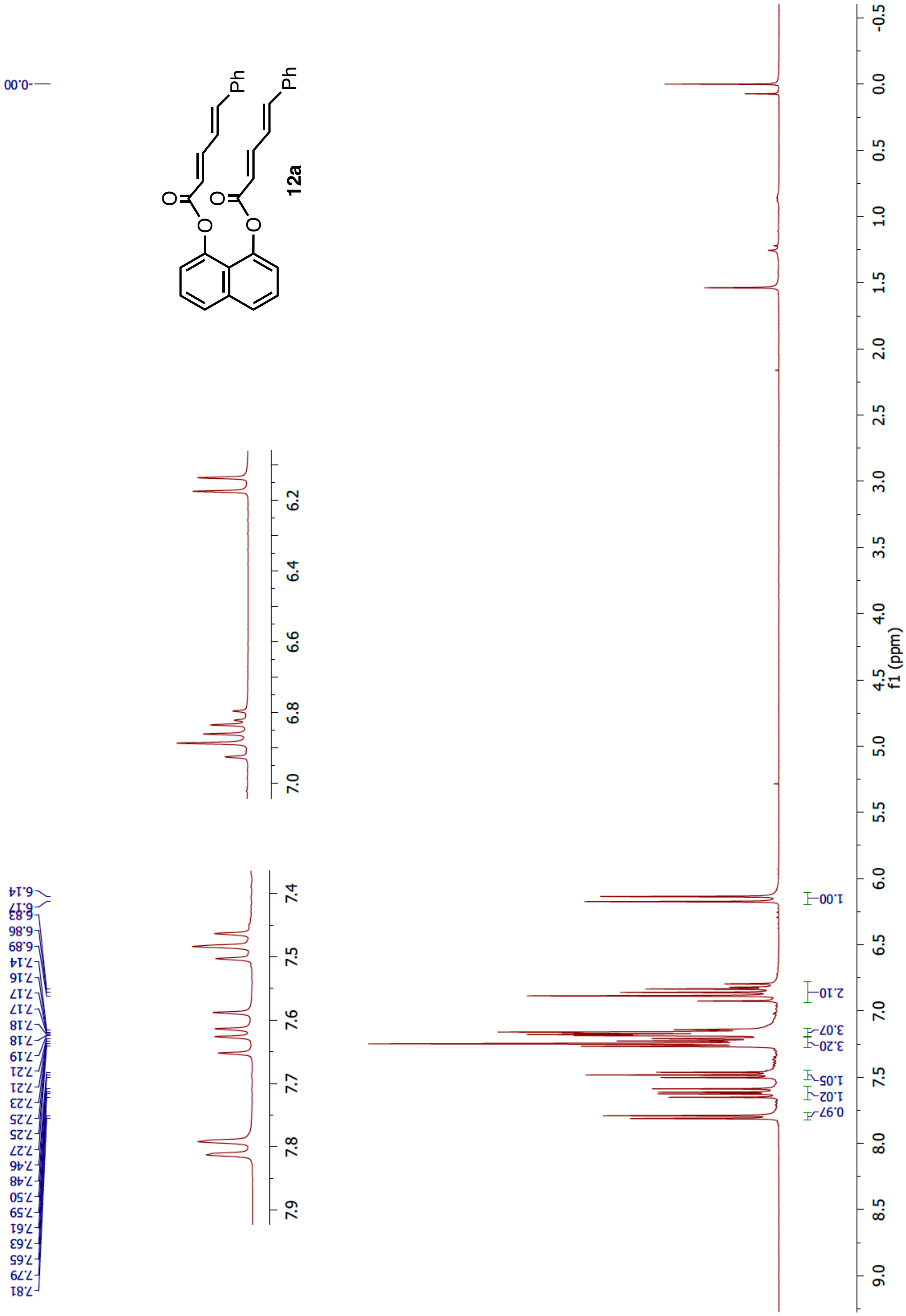

Figure S26. ^{1}H-NMR spectrum of **12a** in $CDCl_3$ (400 MHz).

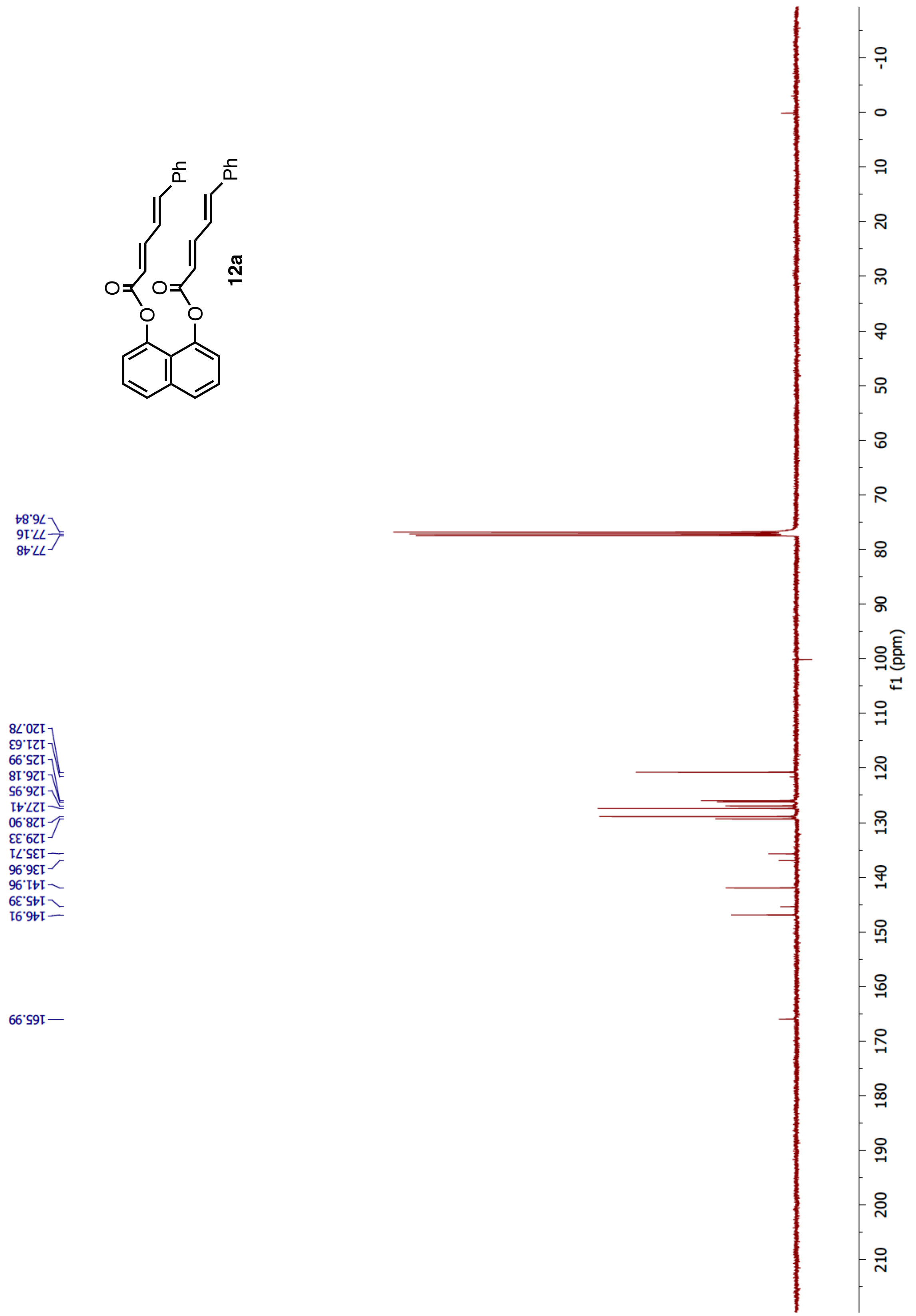

Figure S27. $^{13}C\{^{1}H\}$-NMR spectrum of **12a** in $CDCl_3$ (100 MHz).

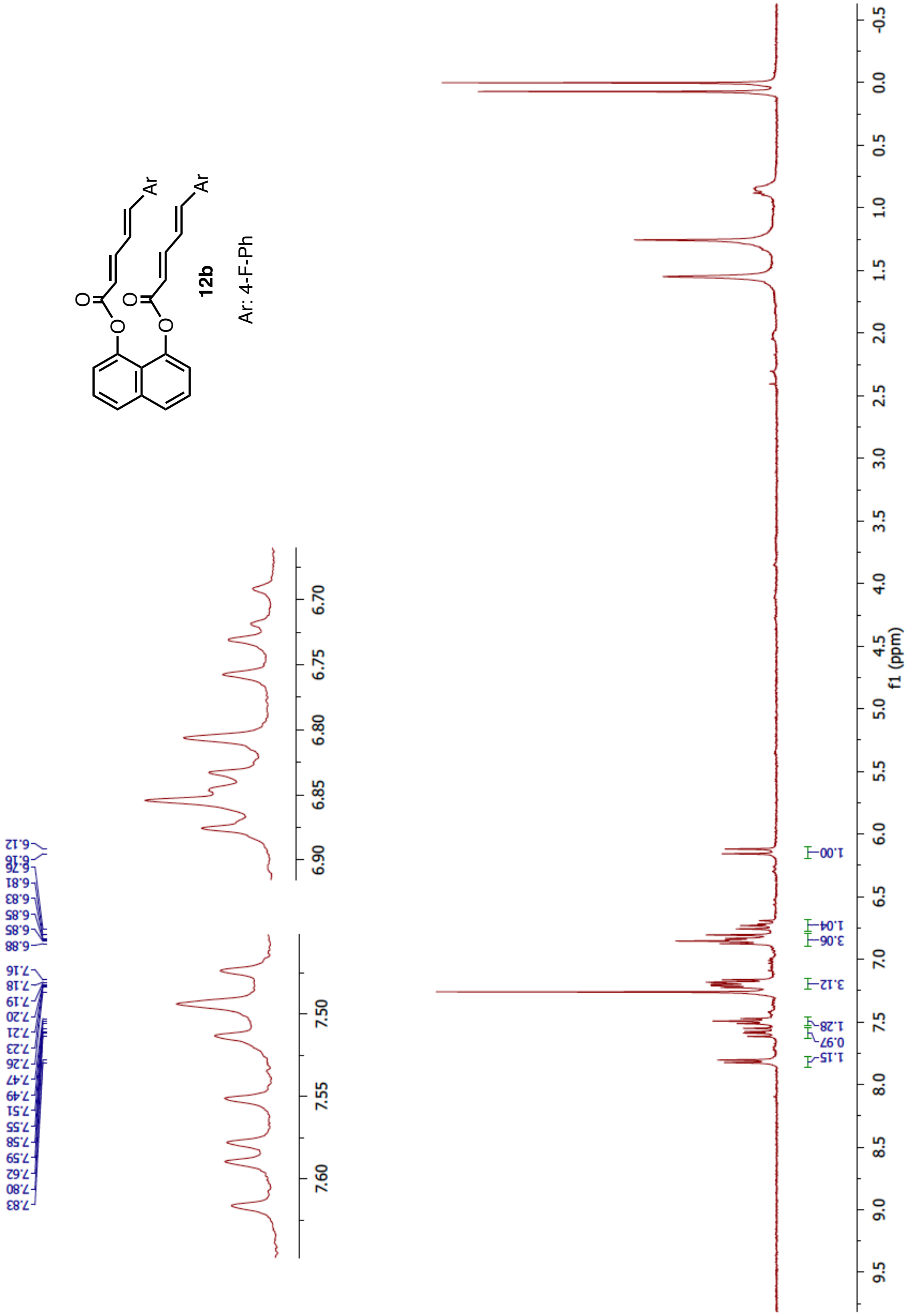

Figure S28. ^{1}H-NMR spectrum of **12b** in $CDCl_3$ (400 MHz).

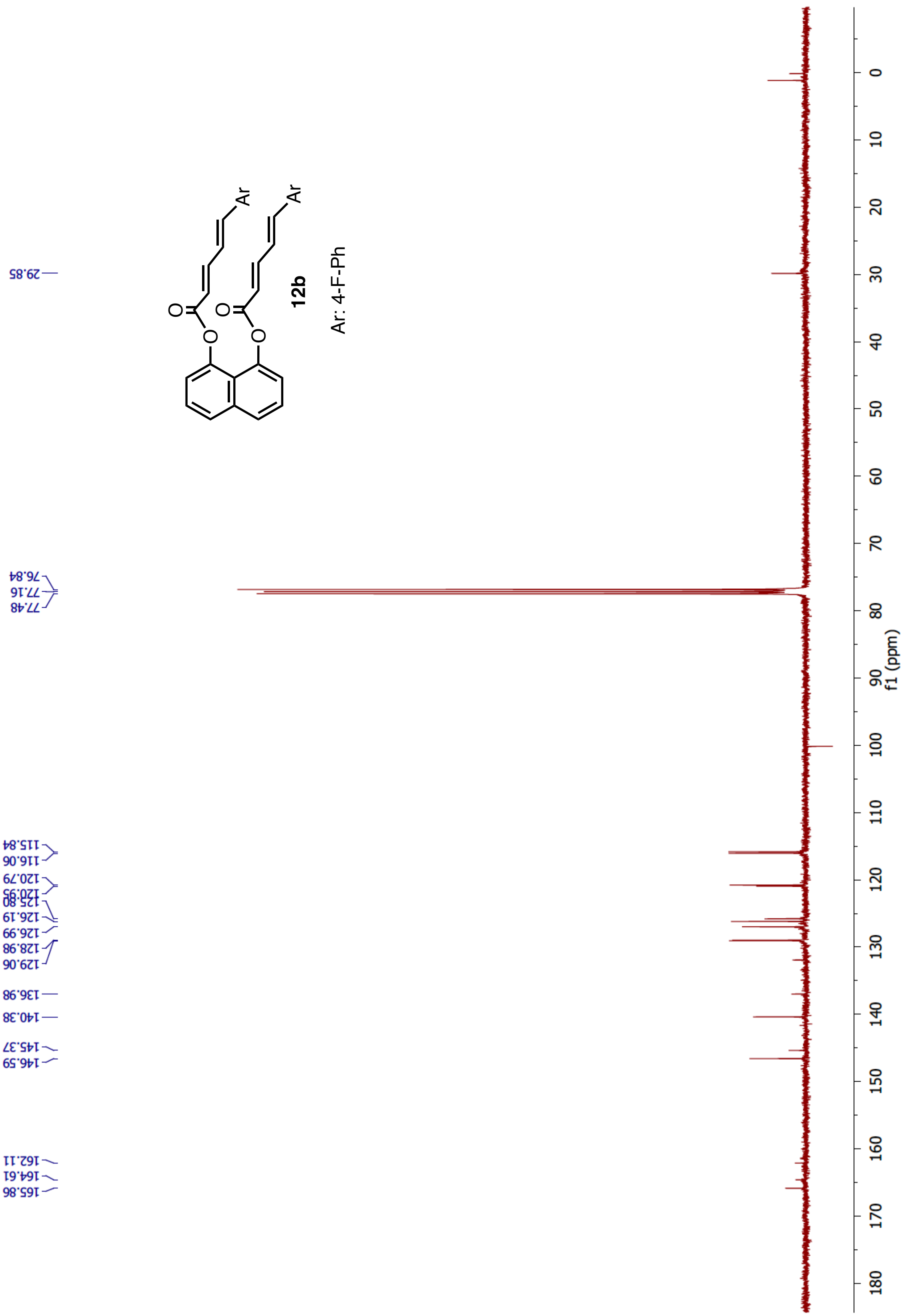

Figure S29. $^{13}C\{^1H\}$-NMR spectrum of **12b** in $CDCl_3$ (100 MHz).

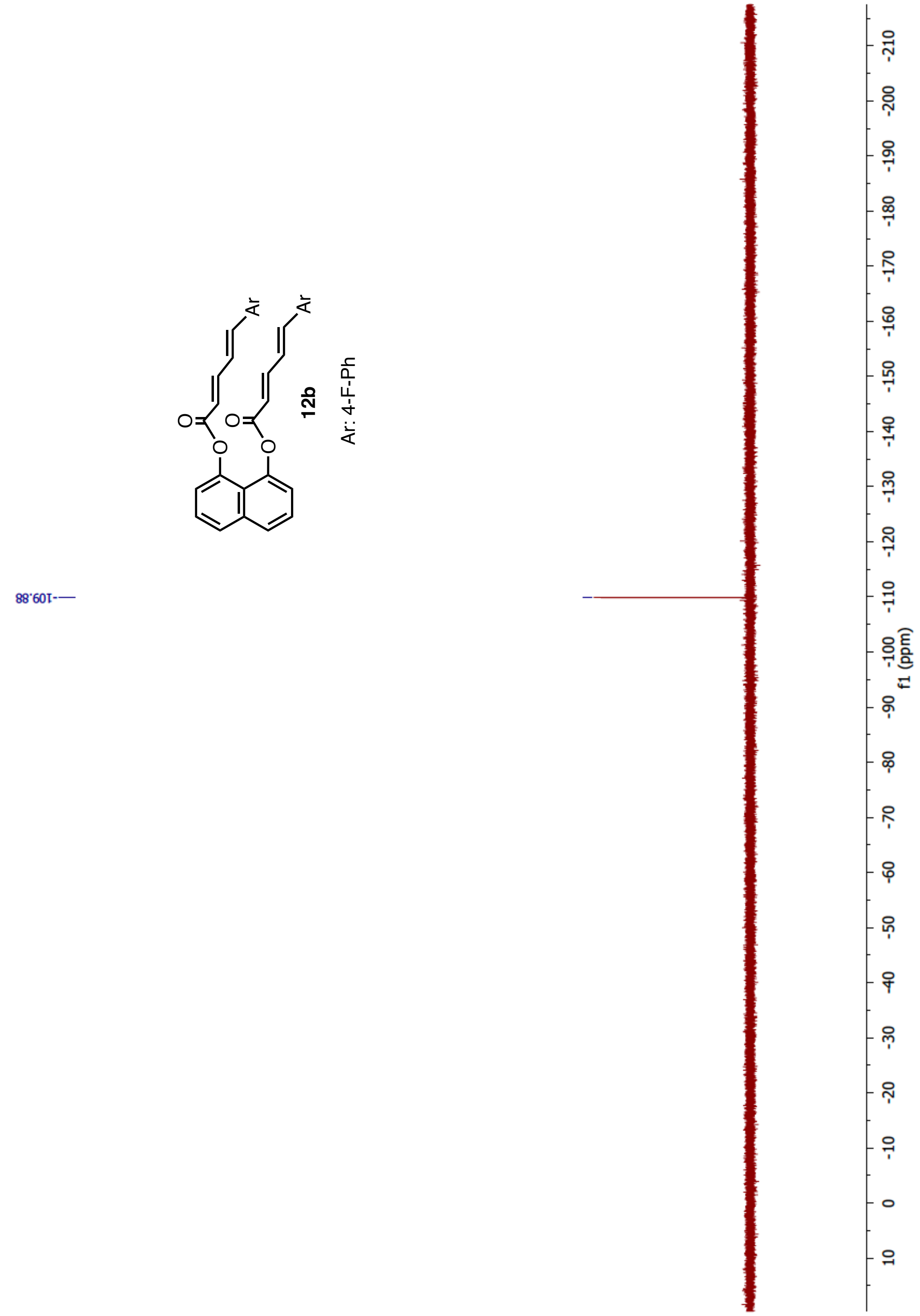

Figure S30. $^{19}F\{^{1}H\}$-NMR spectrum of **12b** in $CDCl_3$ (376 MHz).

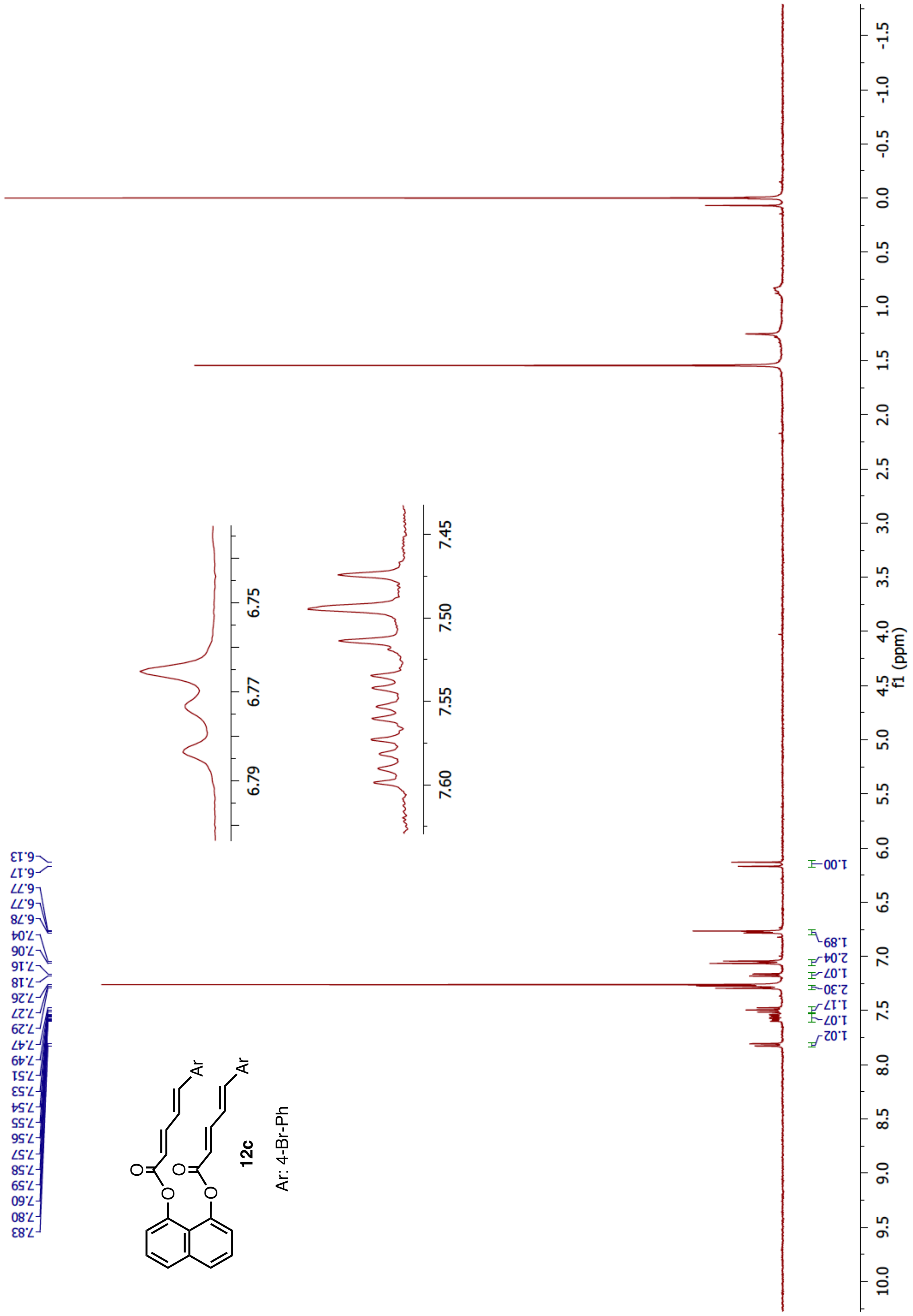

Figure S31. ^{1}H-NMR spectrum of **12c** in $CDCl_3$ (400 MHz).

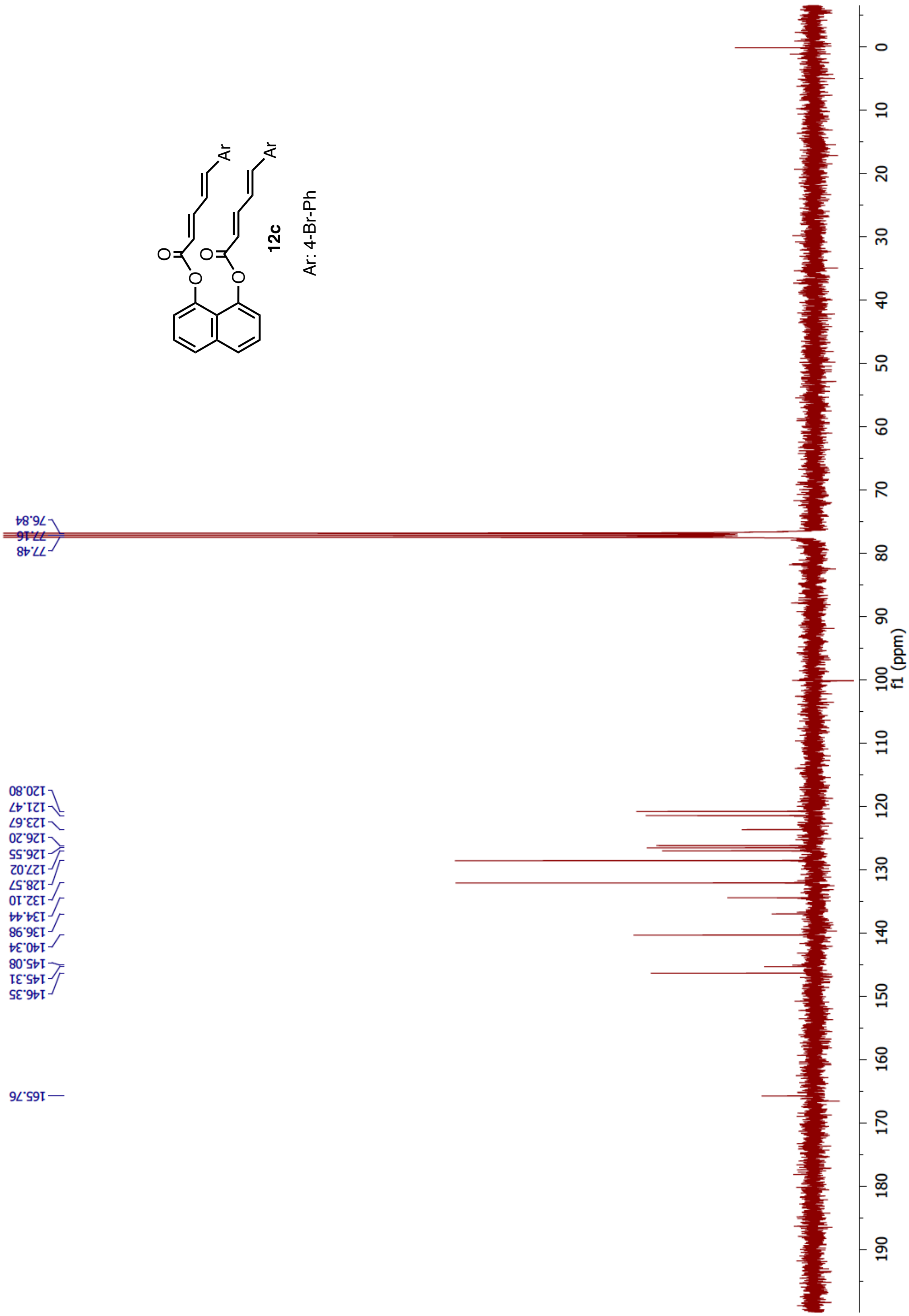

Figure S32. $^{13}C\{^{1}H\}$-NMR spectrum of **12c** in $CDCl_3$ (100 MHz).

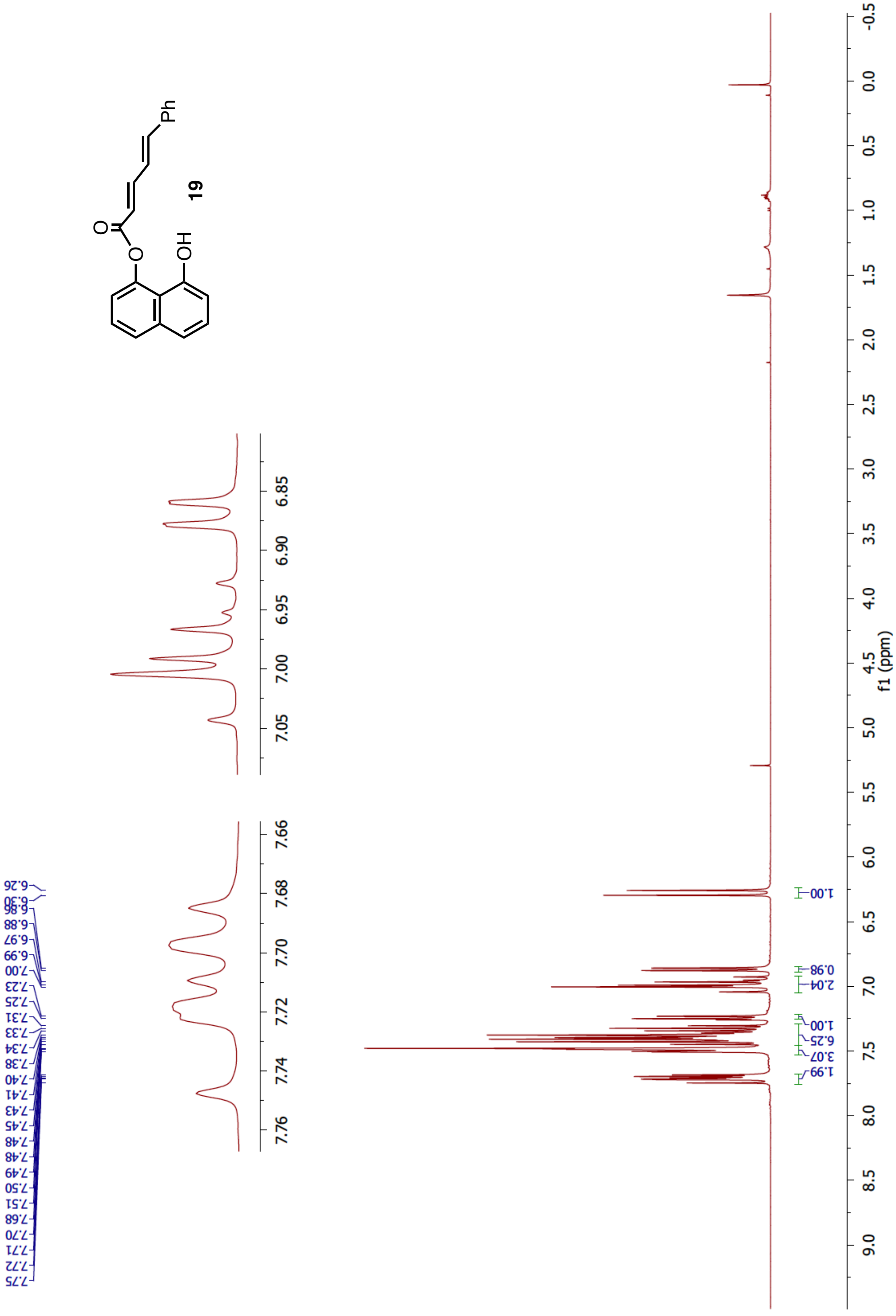

Figure S33. ^{1}H-NMR spectrum of **19** in $CDCl_3$ (400 MHz).

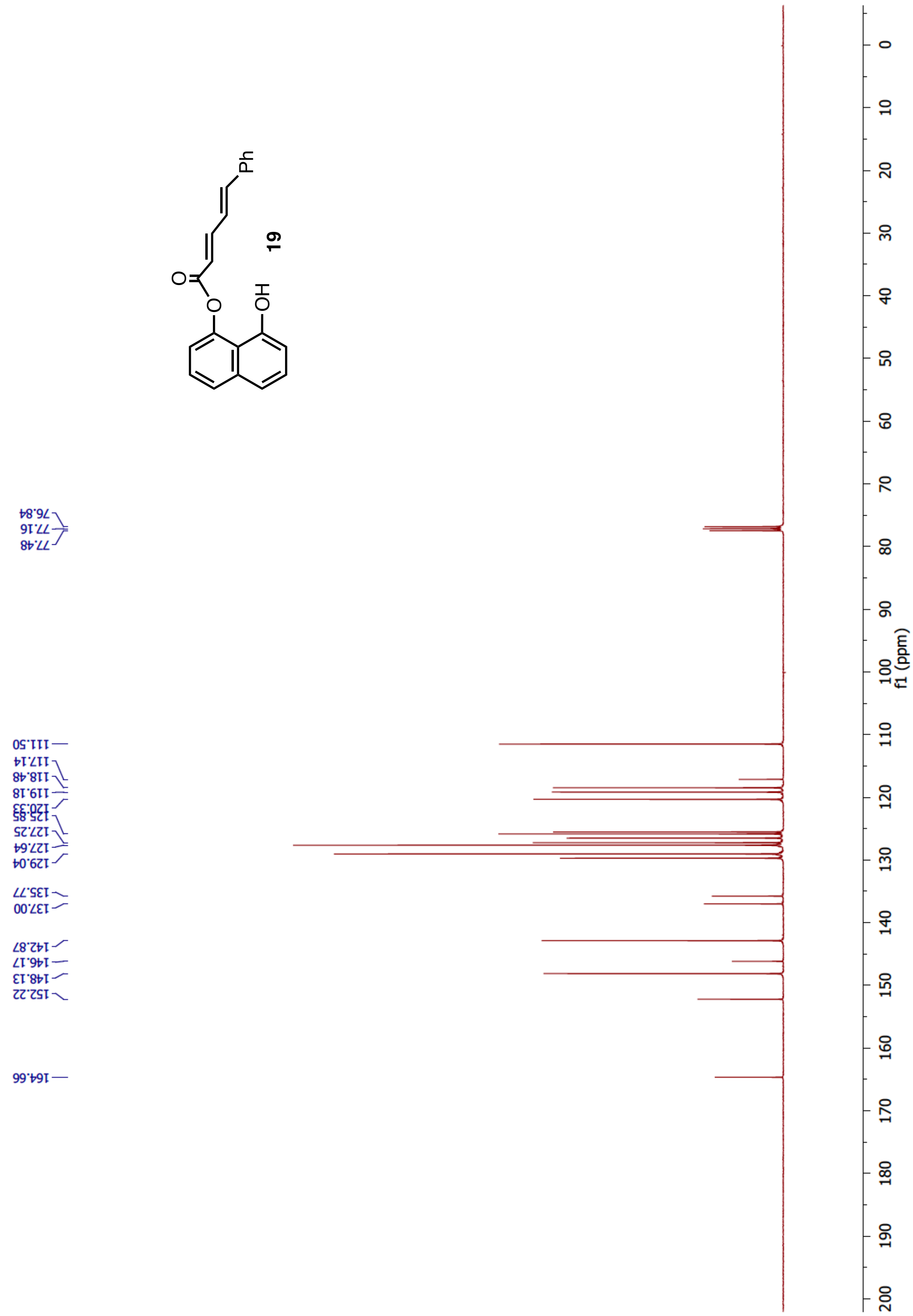

Figure S34. $^{13}C\{^{1}H\}$-NMR spectrum of **19** in $CDCl_3$ (100 MHz).

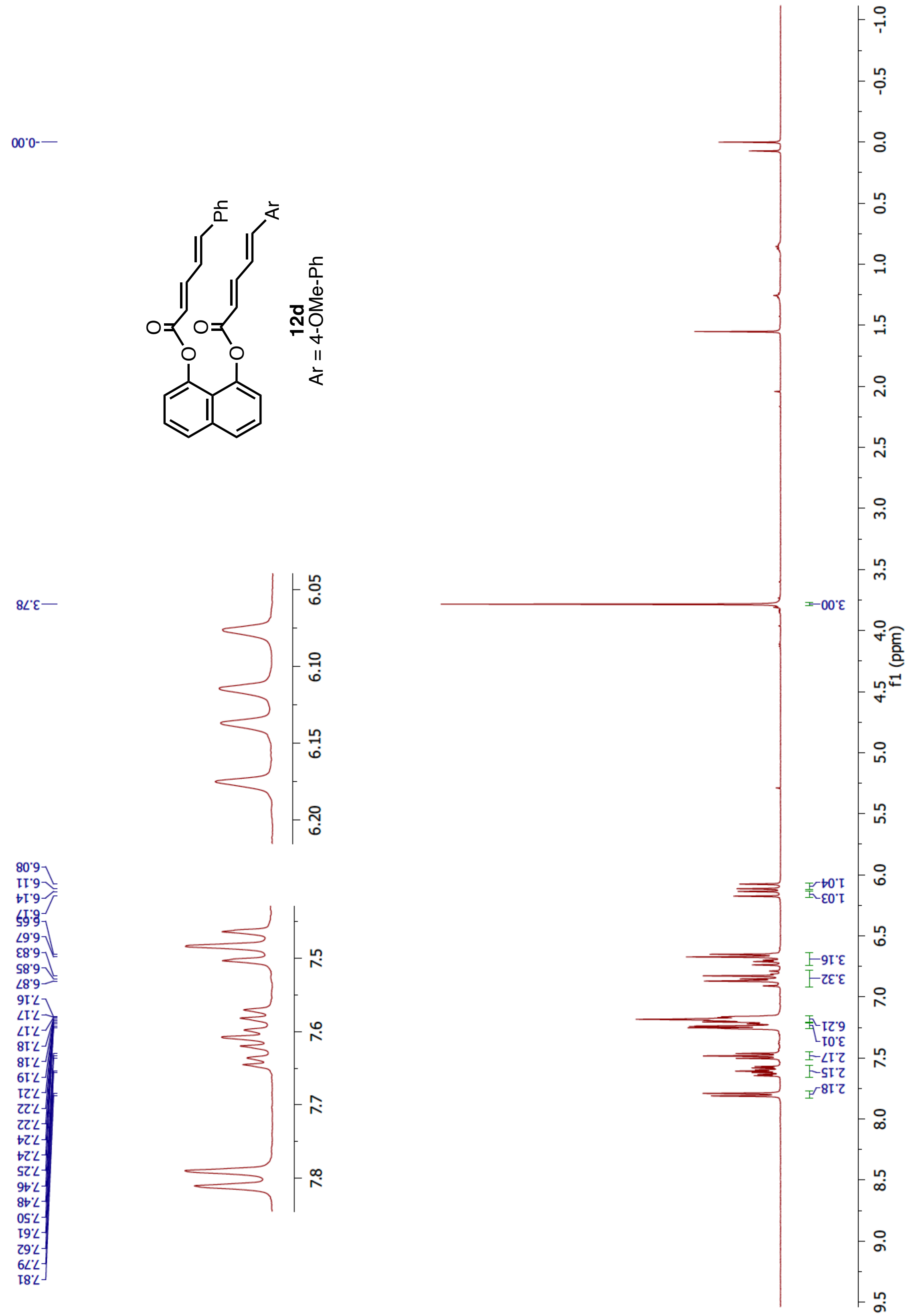

Figure S35. ^{1}H-NMR spectrum of **12d** in $CDCl_3$ (400 MHz).

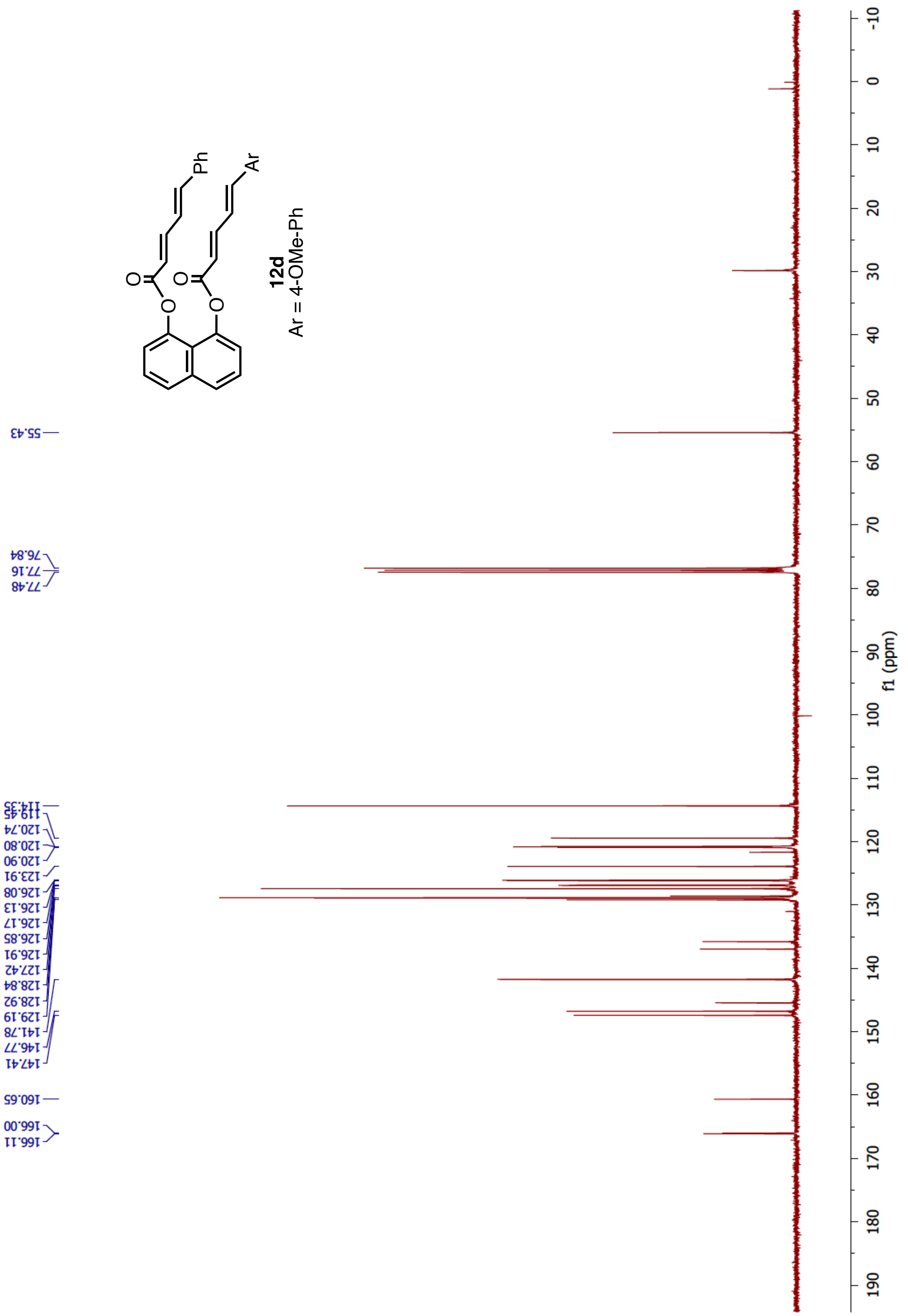

Figure S36. $^{13}C\{^{1}H\}$-NMR spectrum of **12d** in $CDCl_3$ (100 MHz).

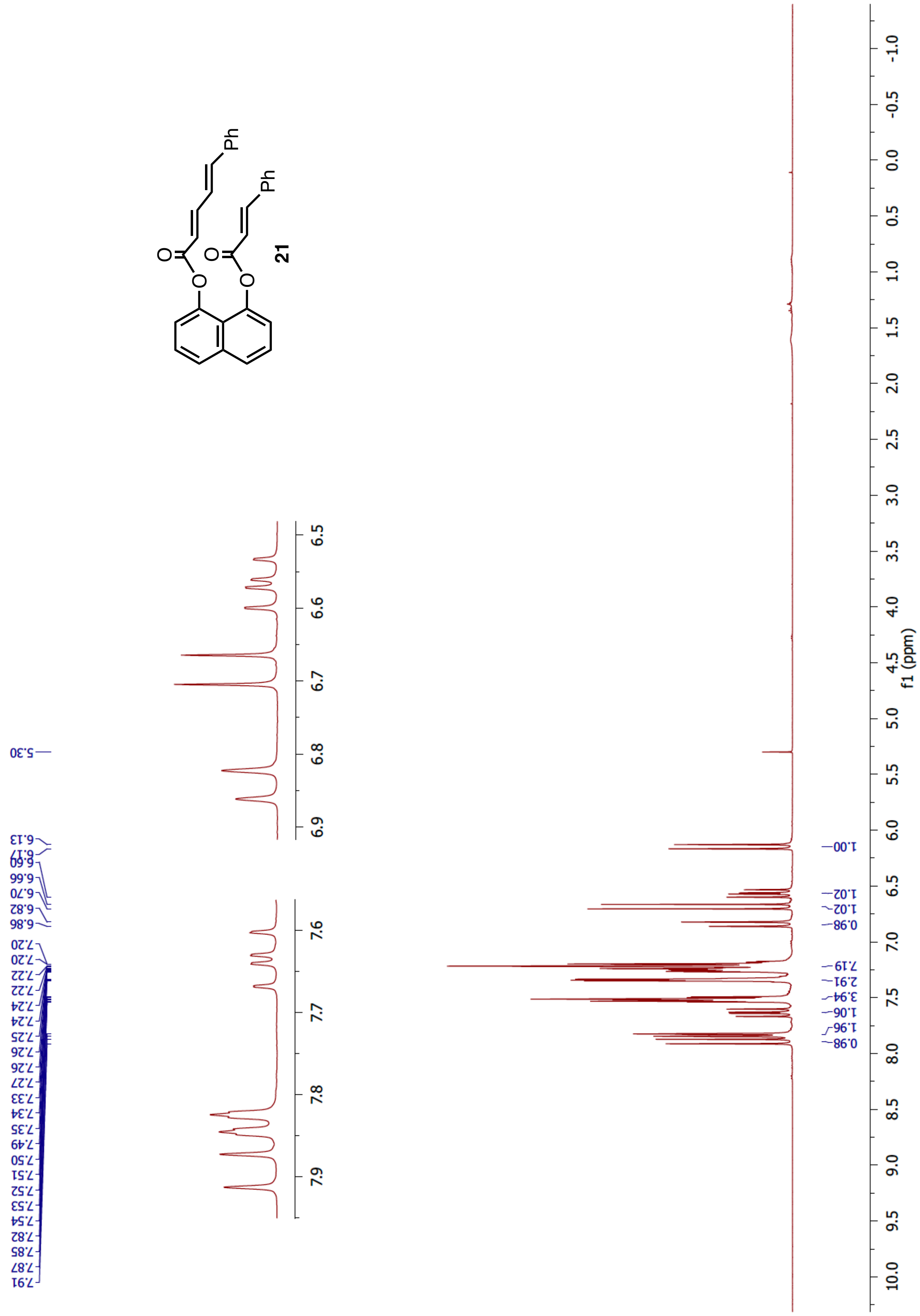

Figure S37. ^{1}H-NMR spectrum of **21** in $CDCl_3$ (400 MHz).

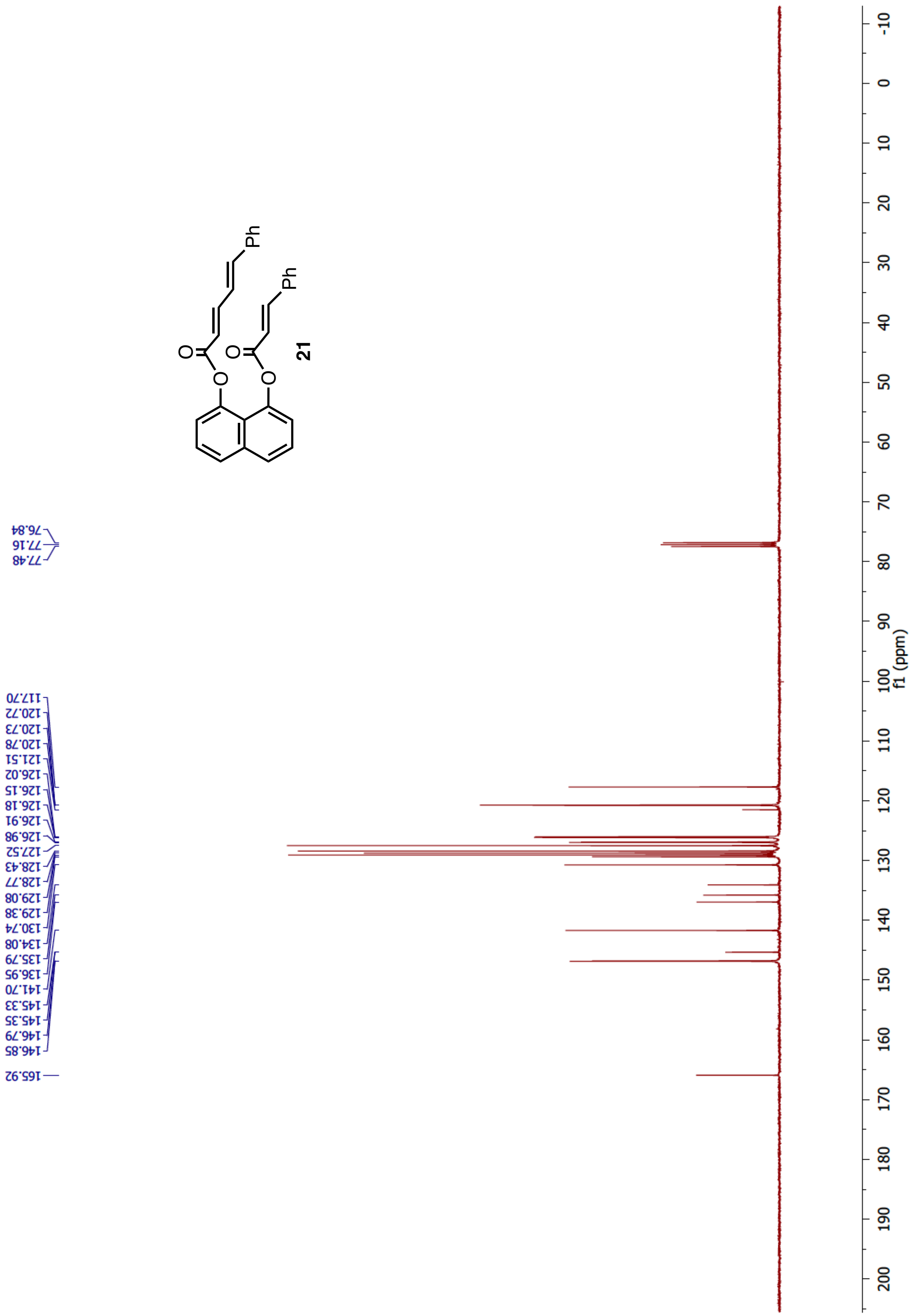

Figure S38. $^{13}C\{^{1}H\}$-NMR spectrum of **21** in $CDCl_3$ (100 MHz).

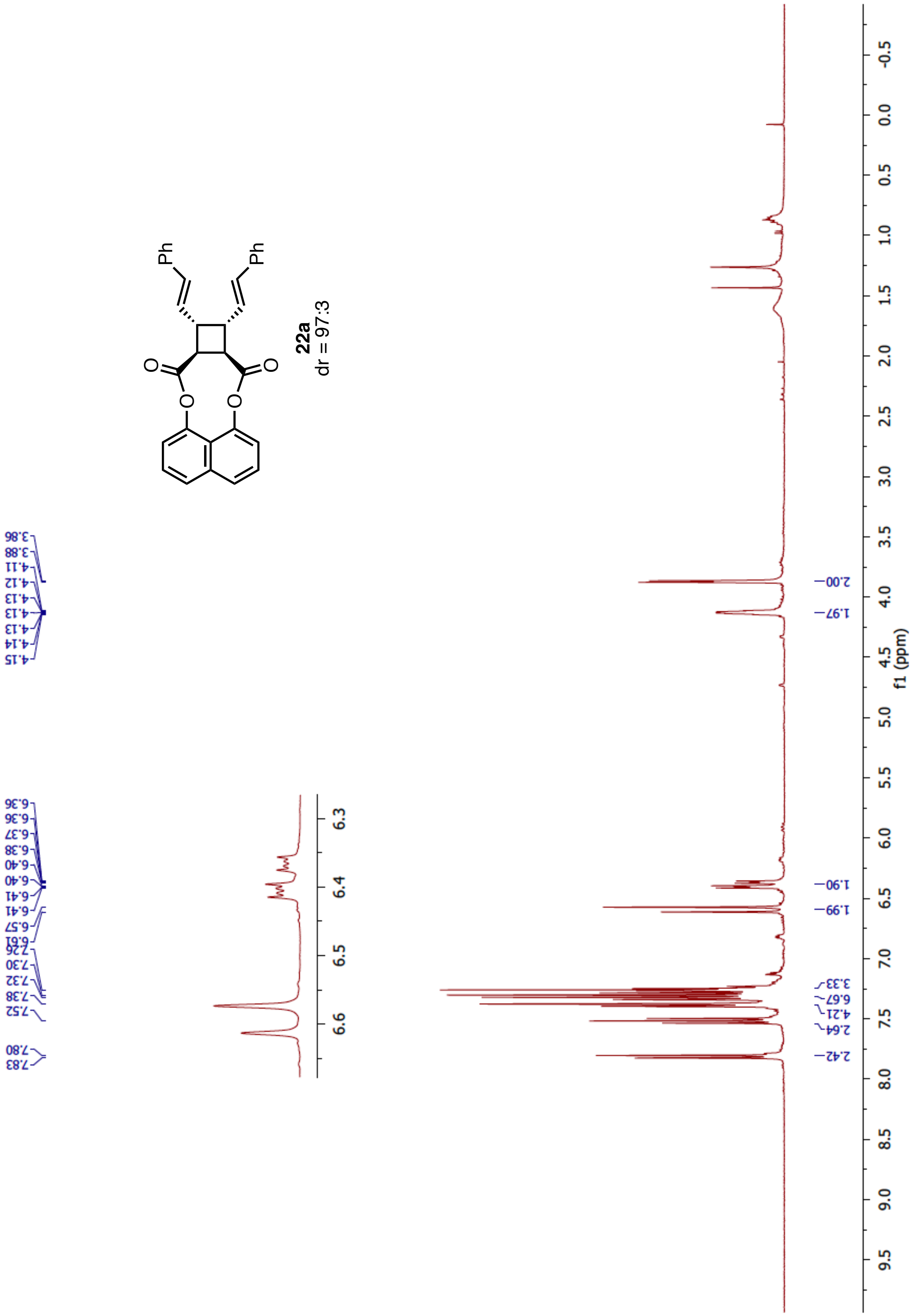

Figure S39. ^{1}H-NMR spectrum of **22a** in $CDCl_3$ (400 MHz).

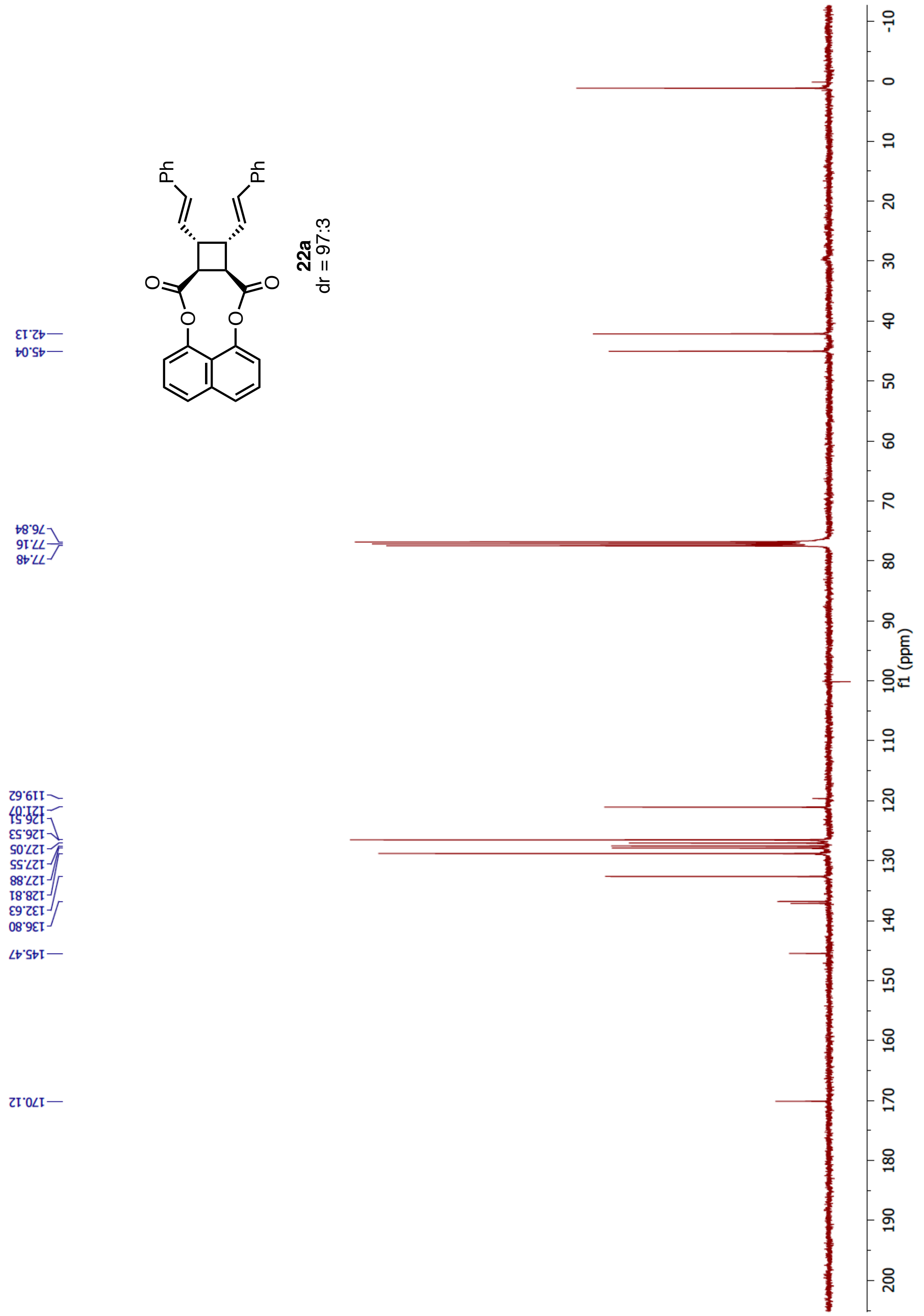

Figure S40. $^{13}C\{^{1}H\}$-NMR spectrum of **22a** in $CDCl_3$ (100 MHz).

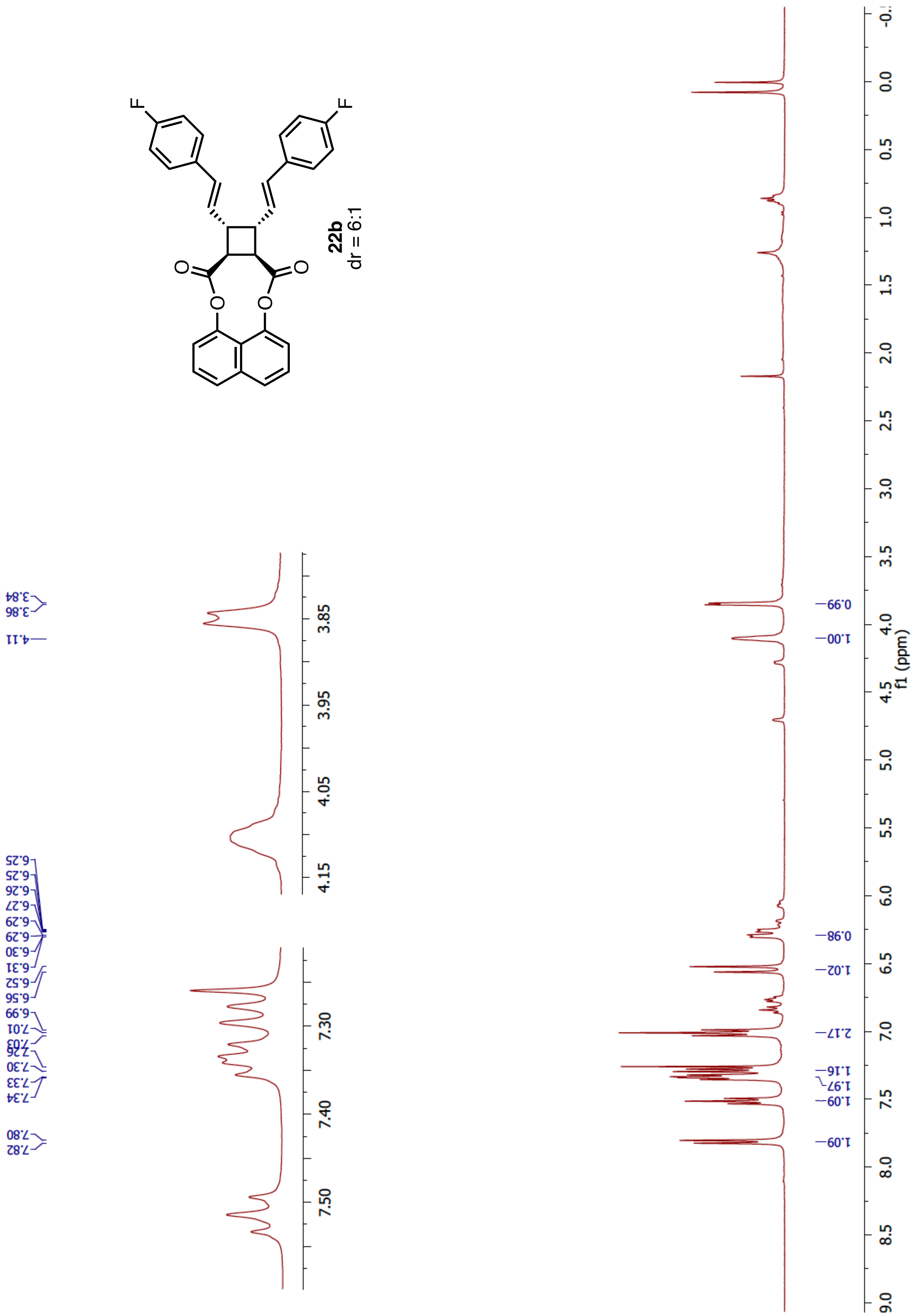

Figure S41. ^{1}H-NMR spectrum of **22b** in $CDCl_3$ (400 MHz).

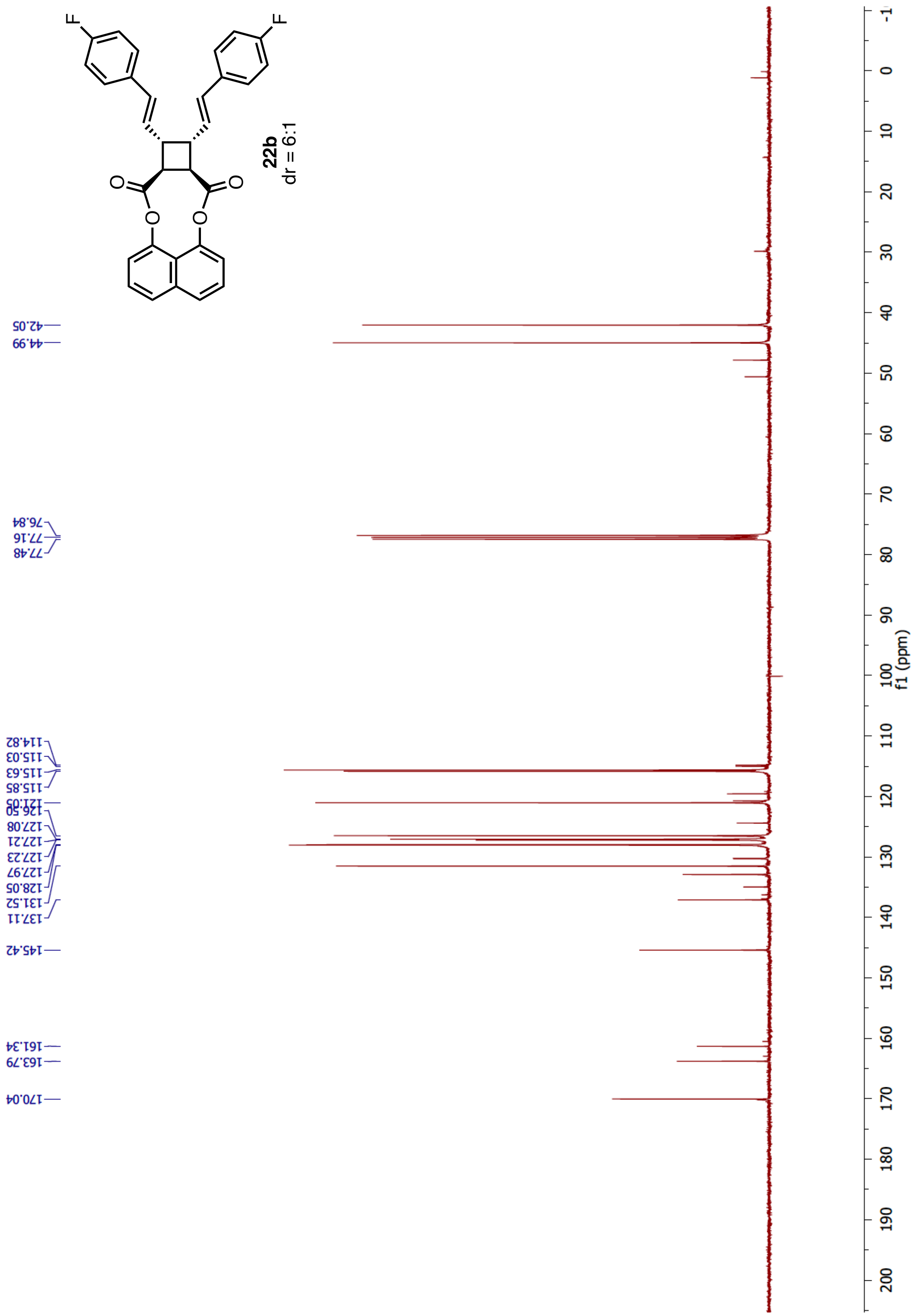

Figure S42. $^{13}C\{^{1}H\}$-NMR spectrum of **22b** in $CDCl_3$ (100 MHz).

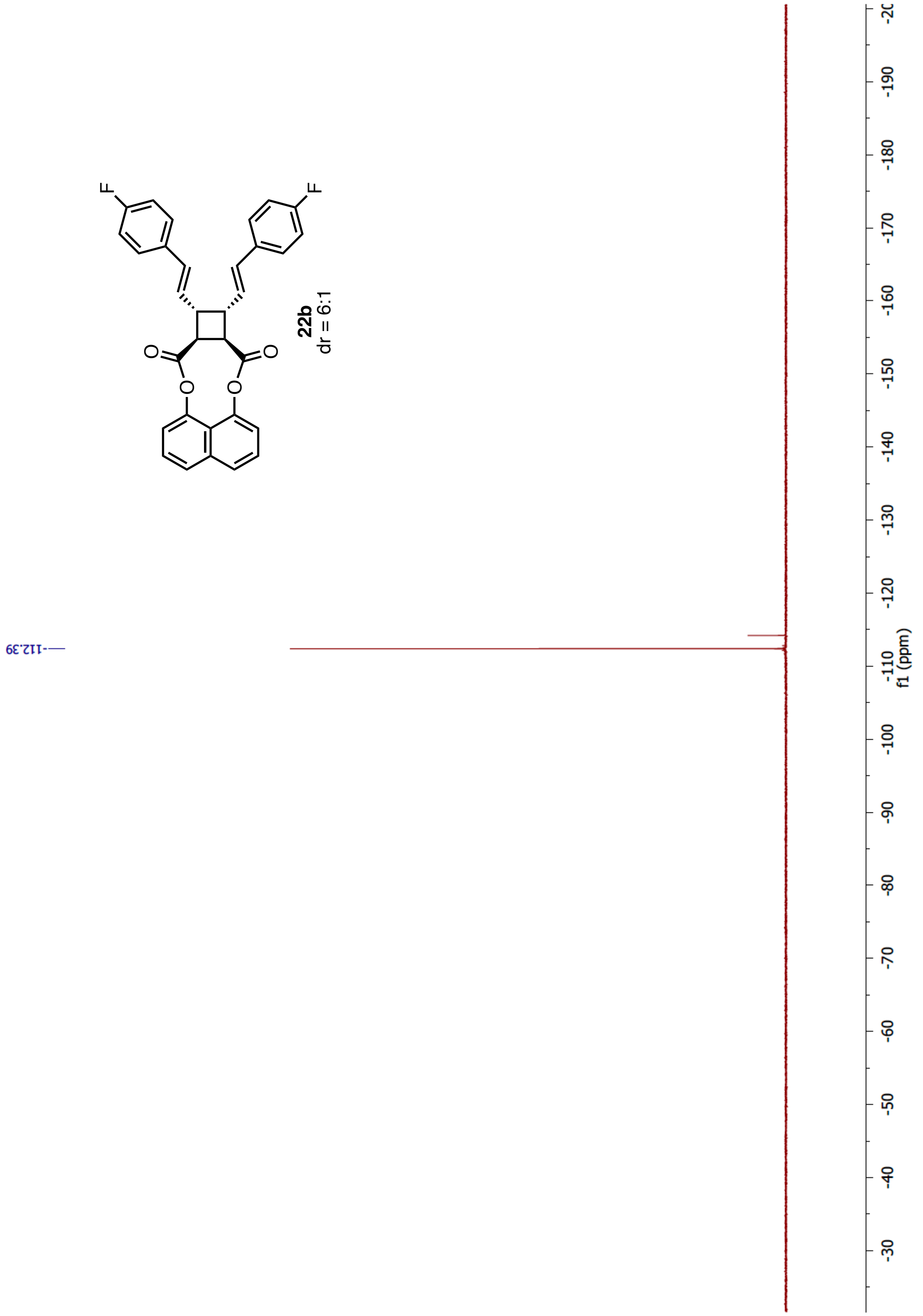

Figure S43. ^{19}F-NMR spectrum of **22b** in $CDCl_3$ (376 MHz).

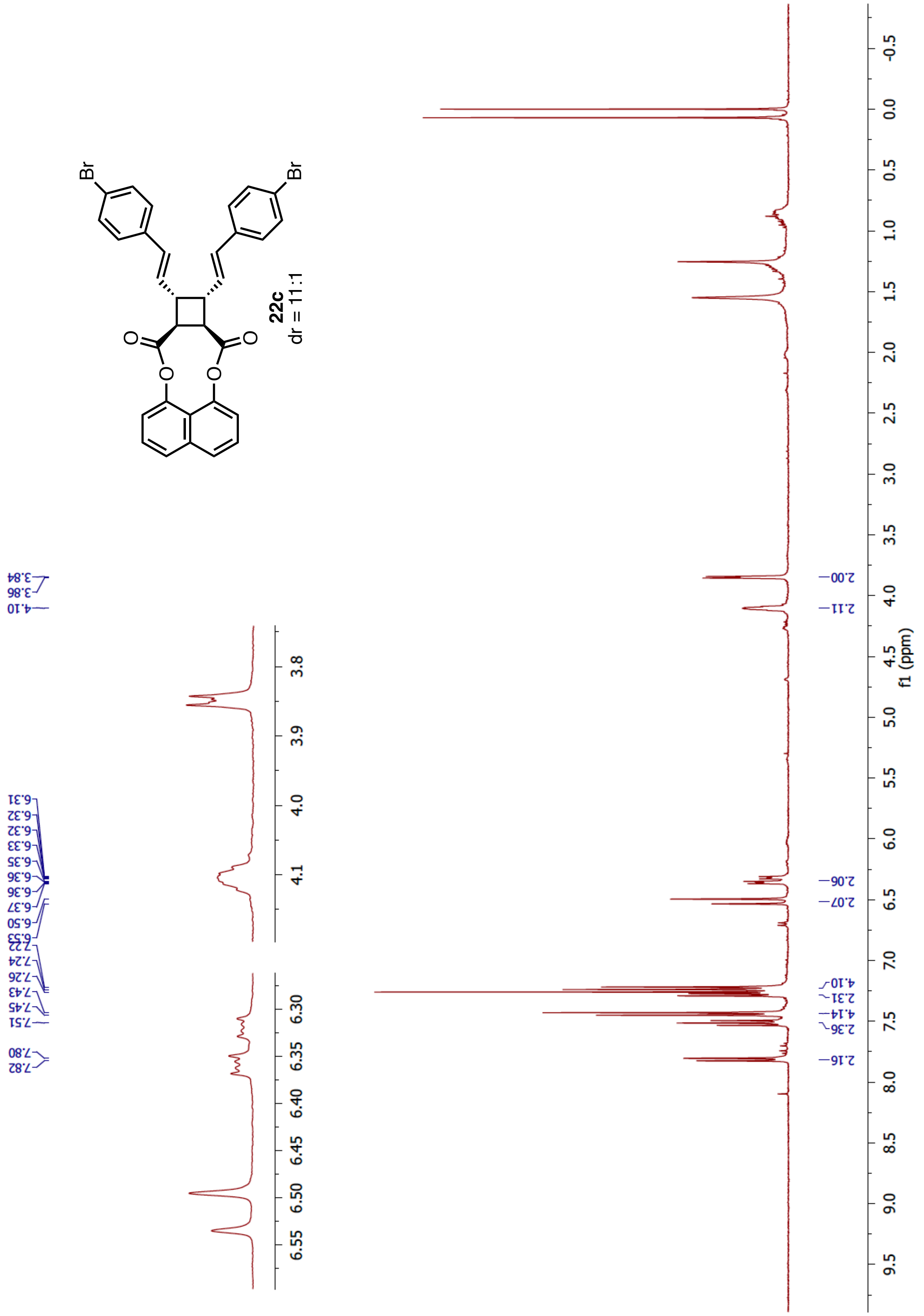

Figure S44. ^{1}H-NMR spectrum of **22c** in $CDCl_3$ (400 MHz).

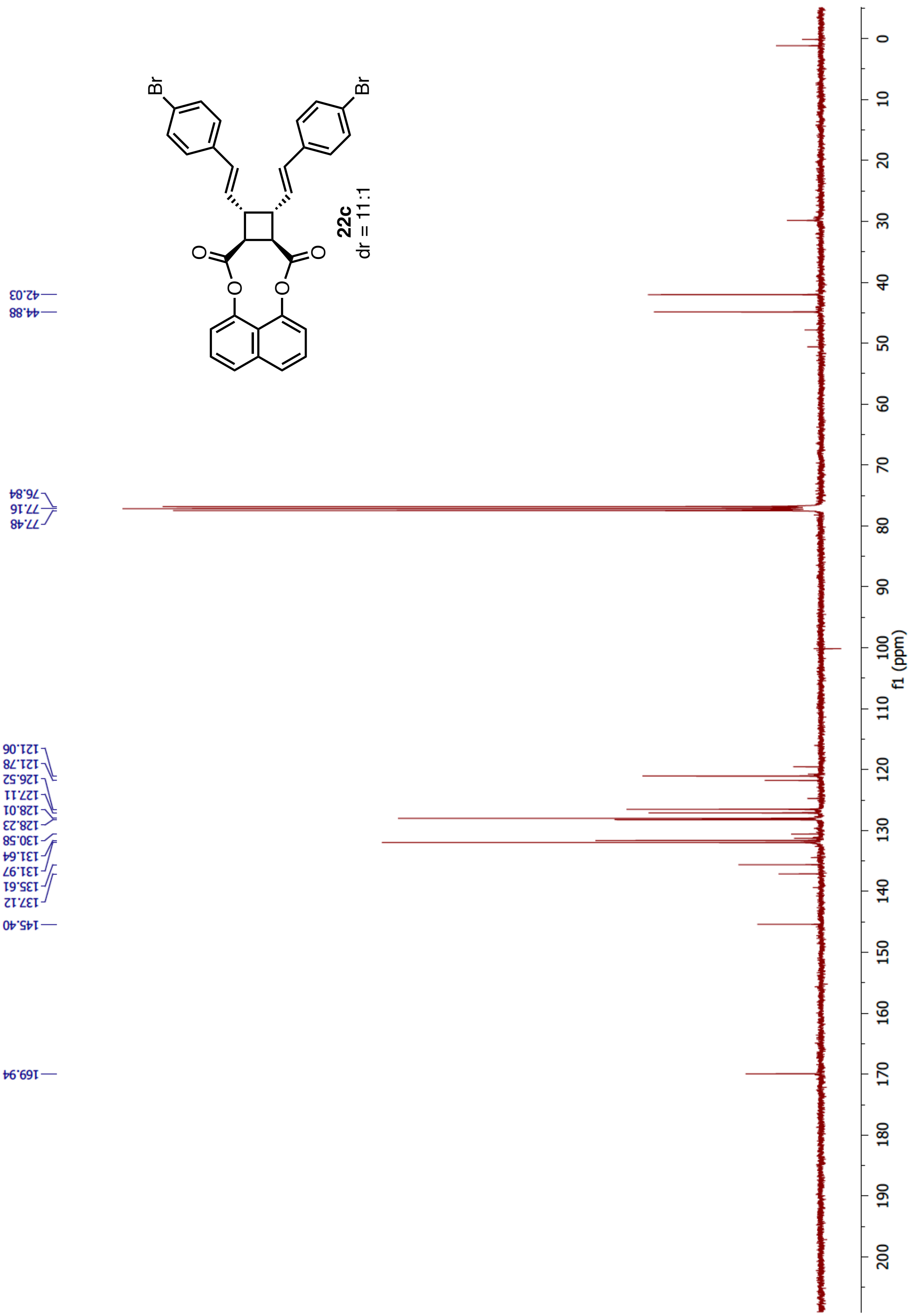

Figure S45. $^{13}C\{^{1}H\}$-NMR spectrum of **22c** in $CDCl_3$ (100 MHz).

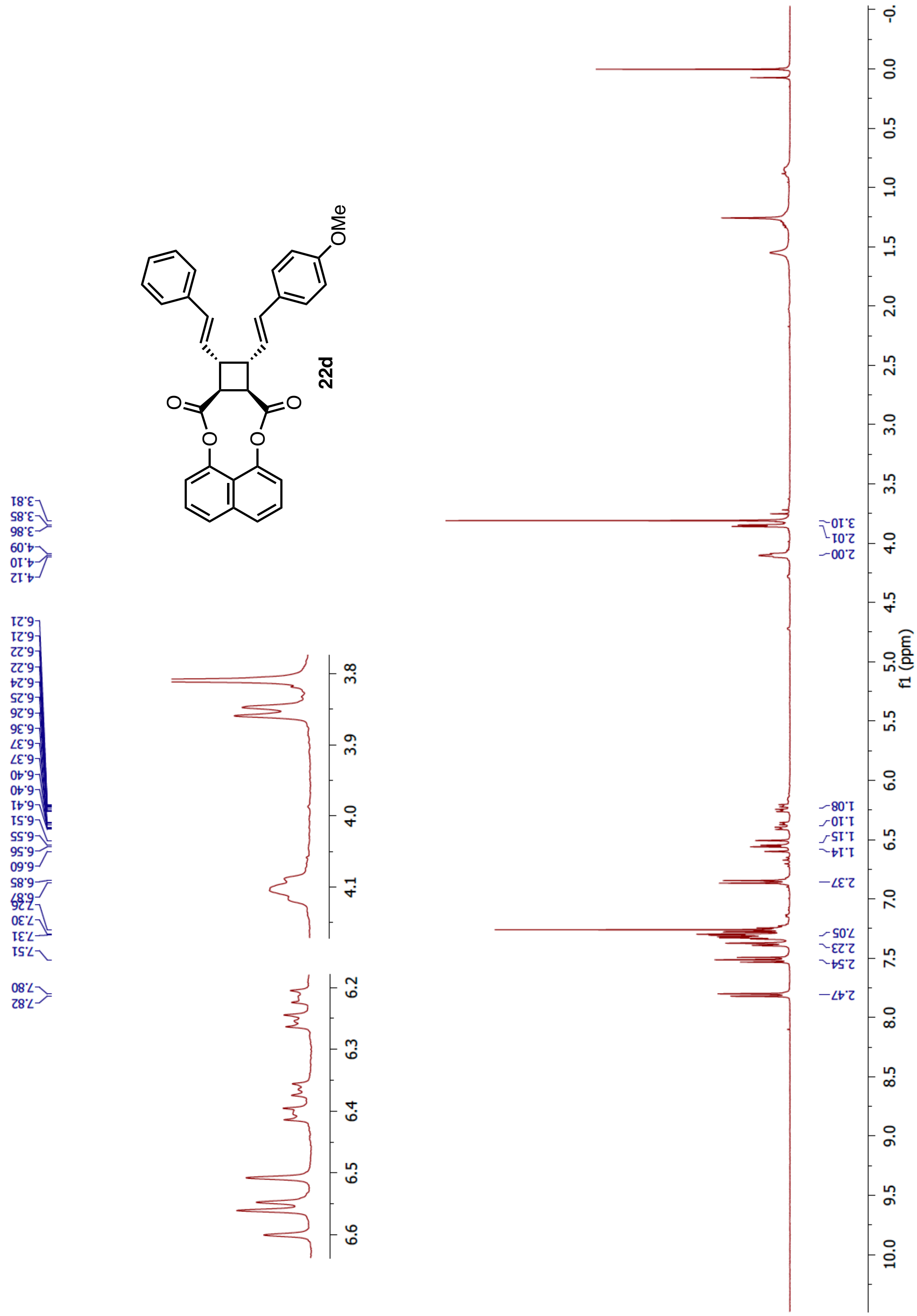

Figure S46. ^{1}H-NMR spectrum of **22d** in $CDCl_3$ (400 MHz).

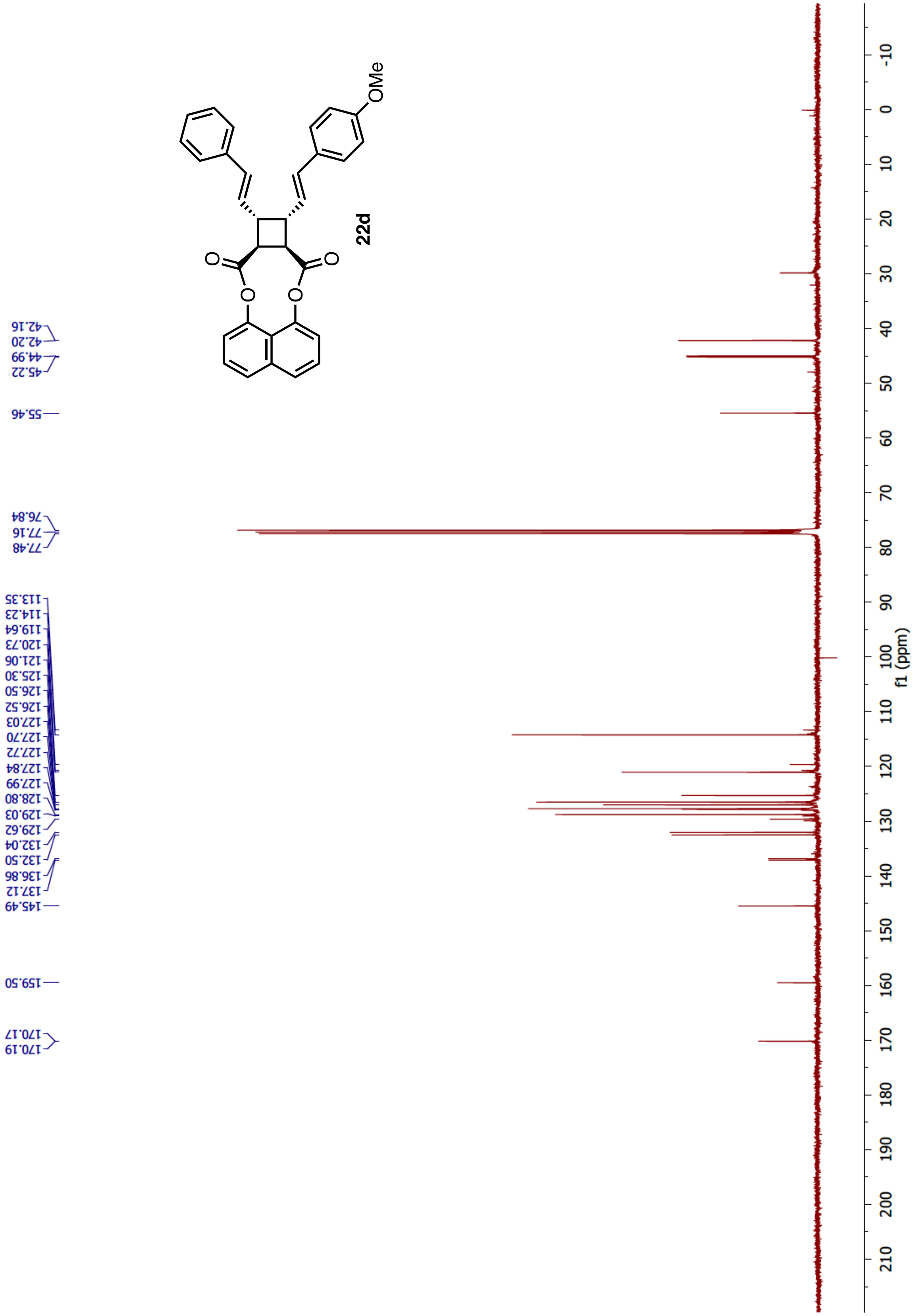

Figure S47. $^{13}C\{^{1}H\}$-NMR spectrum of **22d** in $CDCl_3$ (100 MHz).

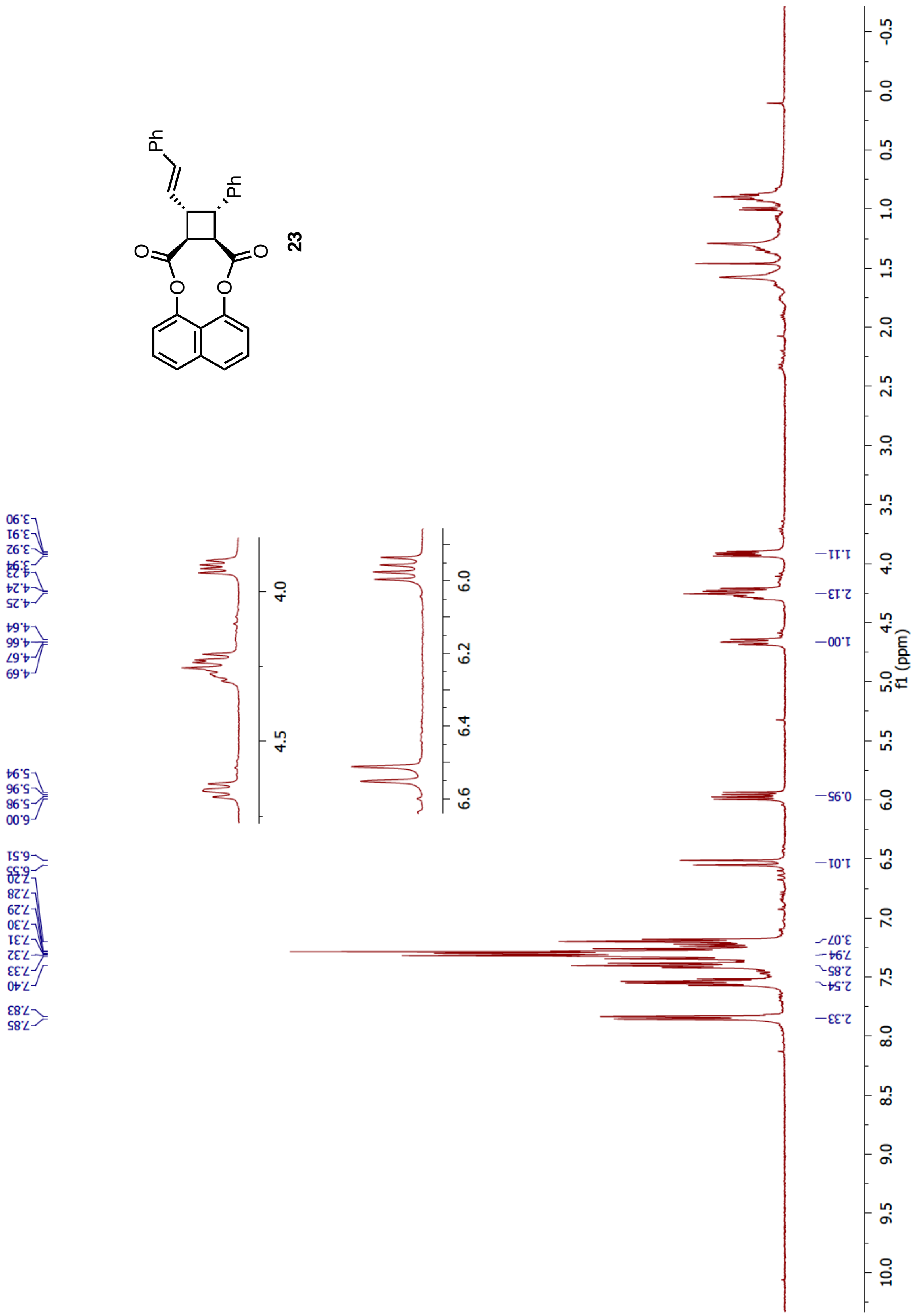

Figure S48. ^{1}H-NMR spectrum of **23** in $CDCl_3$ (400 MHz).

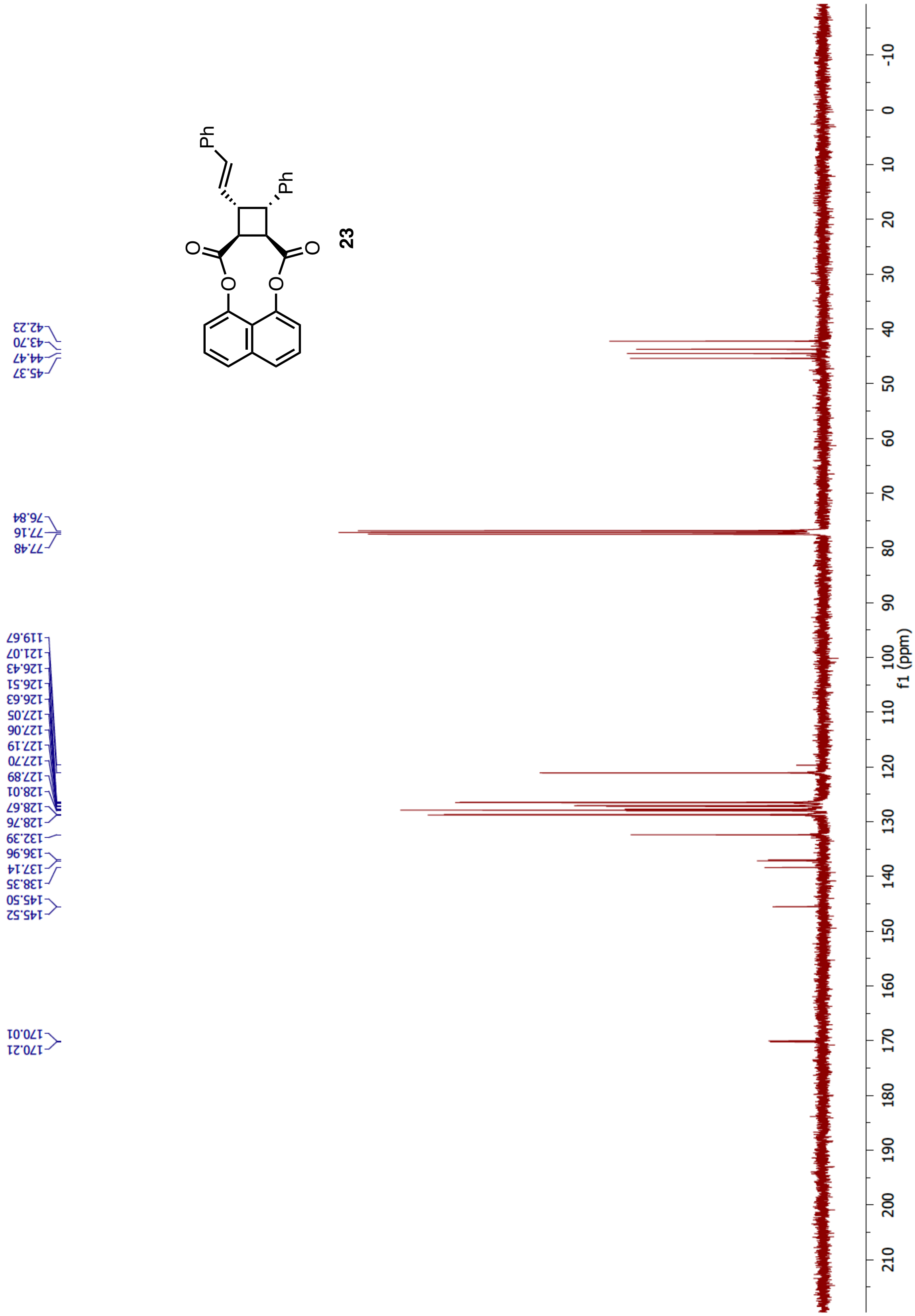

Figure S49. $^{13}C\{^1H\}$-NMR spectrum of **23** in $CDCl_3$ (100 MHz).

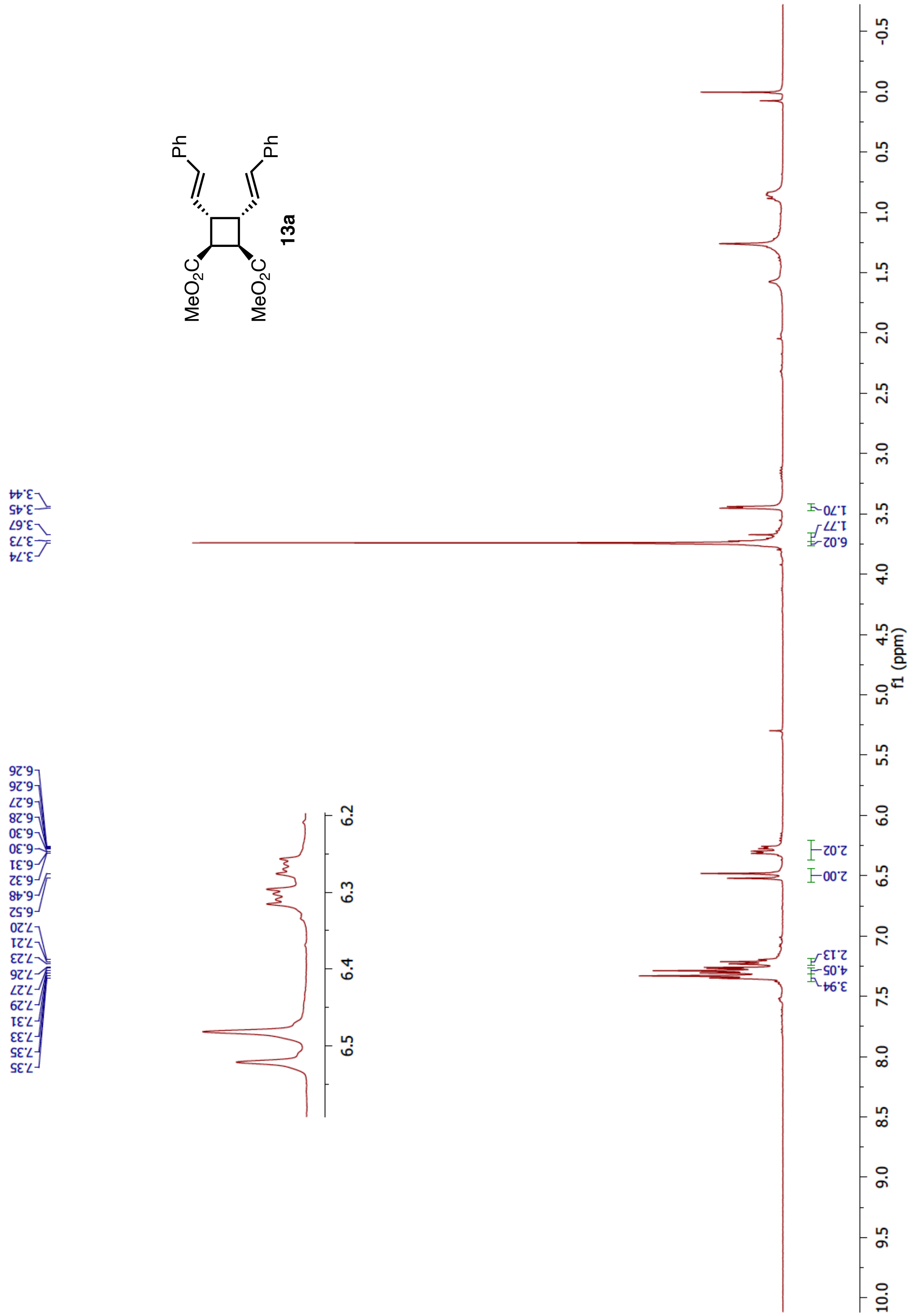

Figure S50. ^{1}H-NMR spectrum of **13a** in $CDCl_3$ (400 MHz).

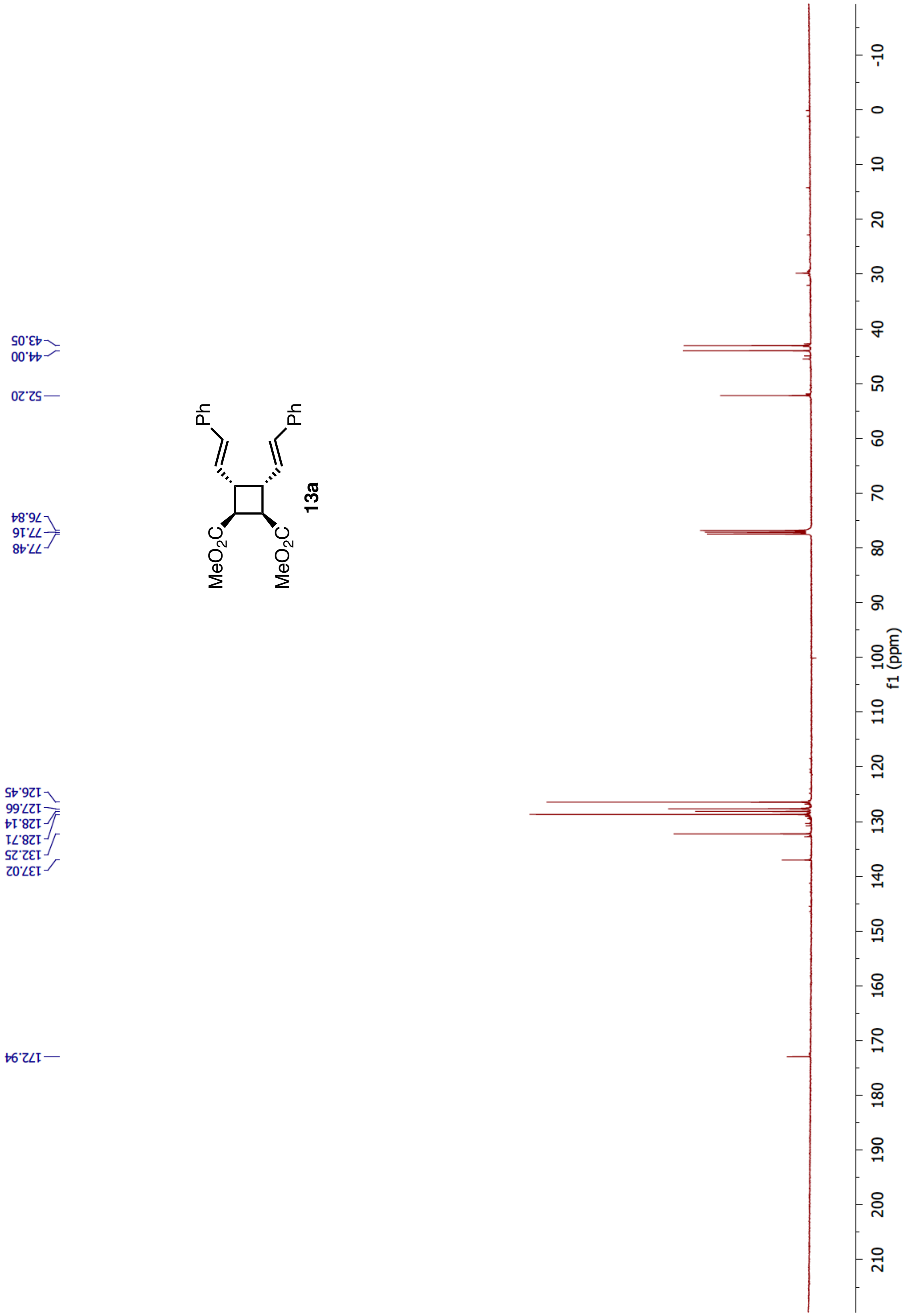

Figure S51. $^{13}C\{^{1}H\}$-NMR spectrum of **13a** in $CDCl_3$ (100 MHz).

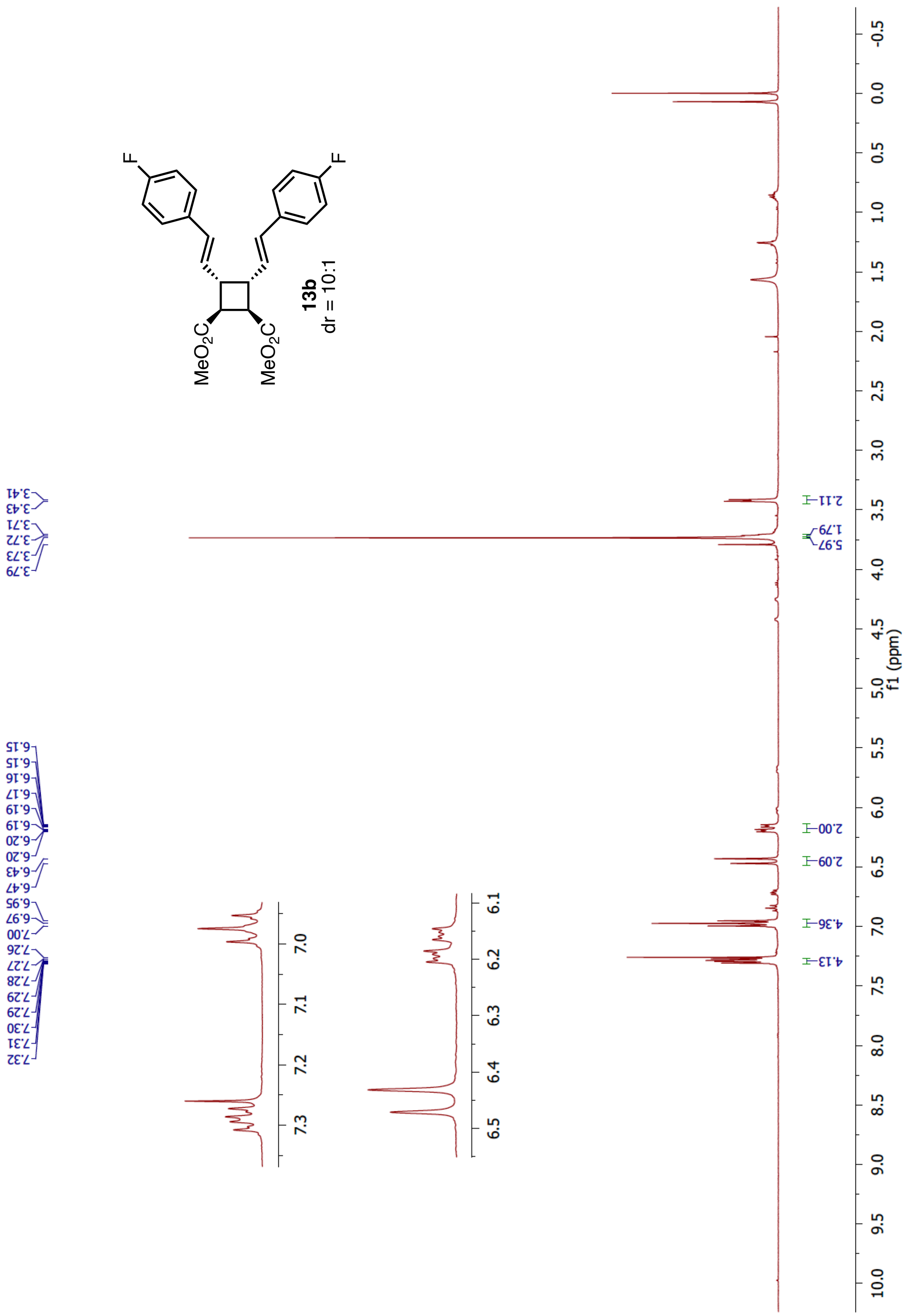

Figure S52. ^{1}H-NMR spectrum of **13b** in $CDCl_3$ (400 MHz).

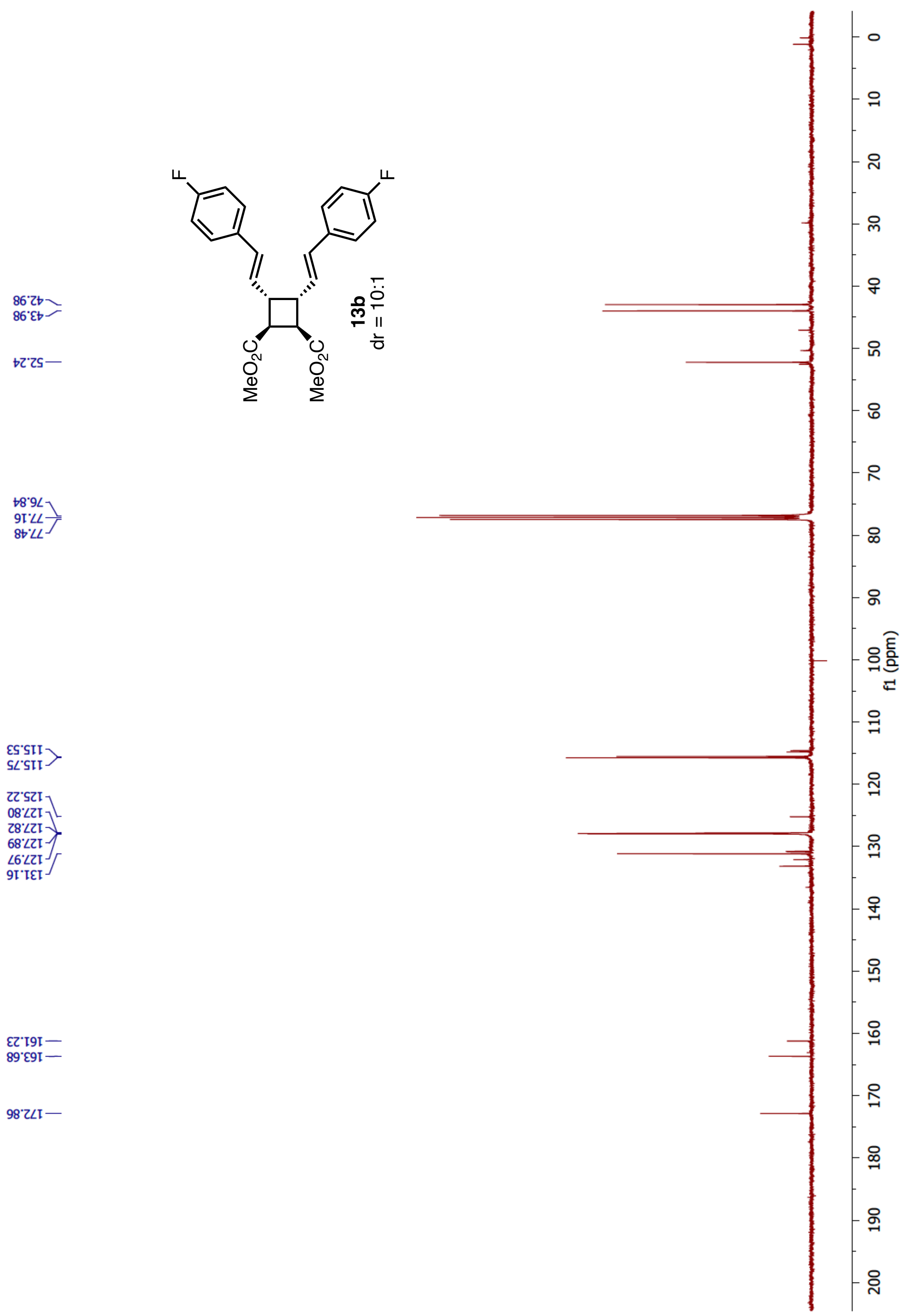

Figure S53. $^{13}C\{^{1}H\}$-NMR spectrum of **13b** in $CDCl_3$ (100 MHz).

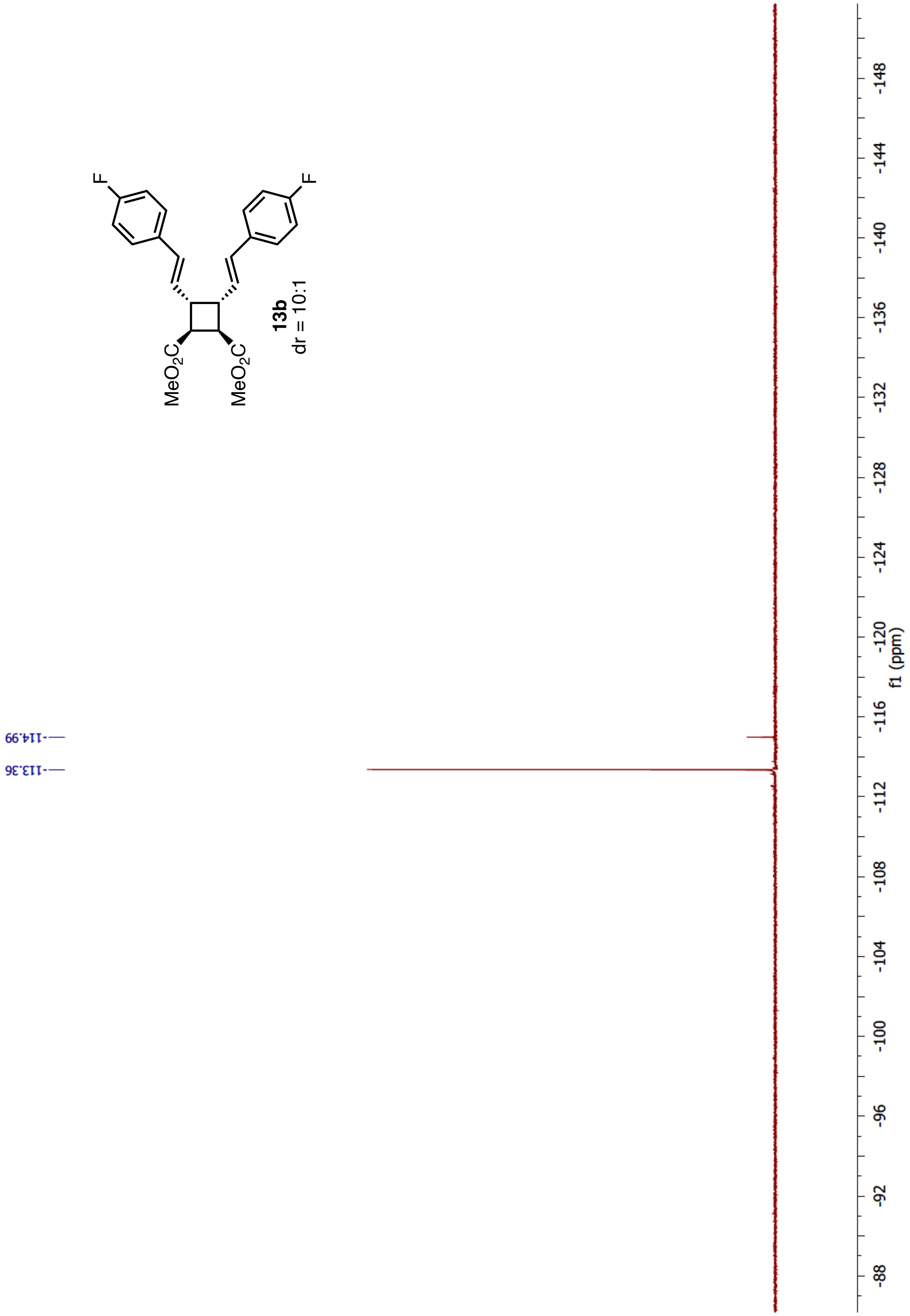

Figure S54. ^{19}F-NMR spectrum of **13b** in $CDCl_3$ (376 MHz).

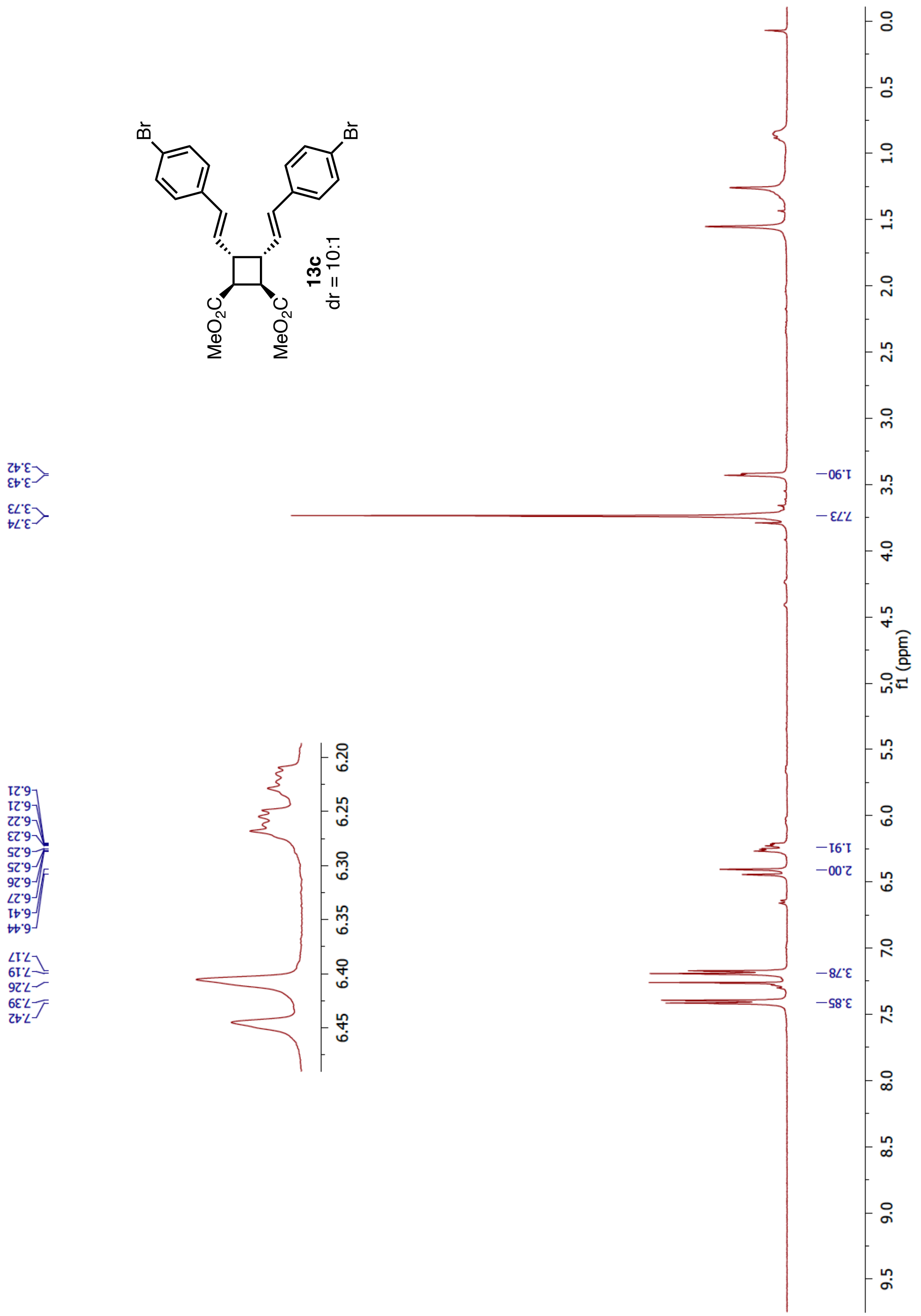

Figure S55. ^{1}H-NMR spectrum of **13c** in $CDCl_3$ (400 MHz).

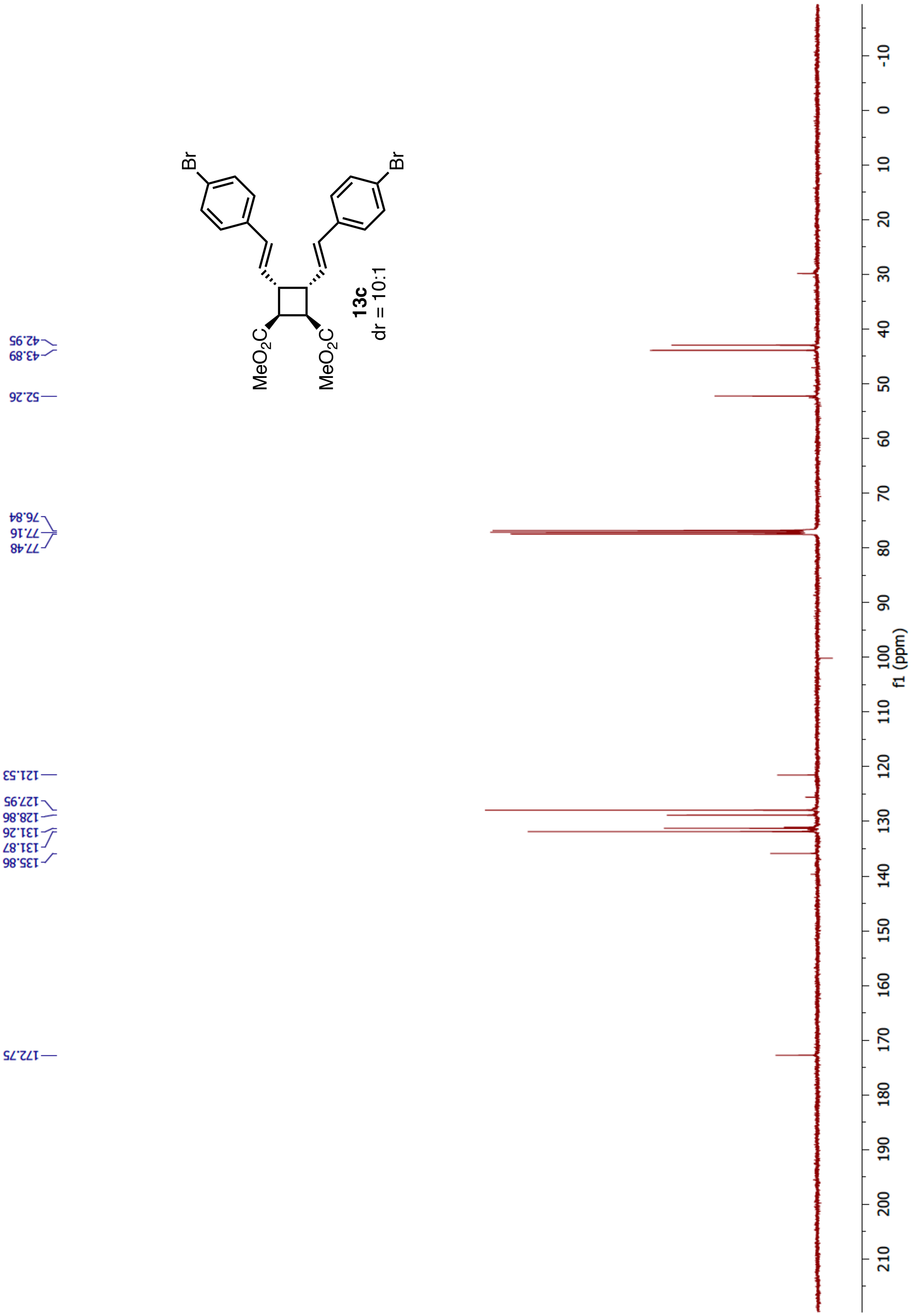

Figure S56. $^{13}C\{^{1}H\}$-NMR spectrum of **13c** in $CDCl_3$ (100 MHz).

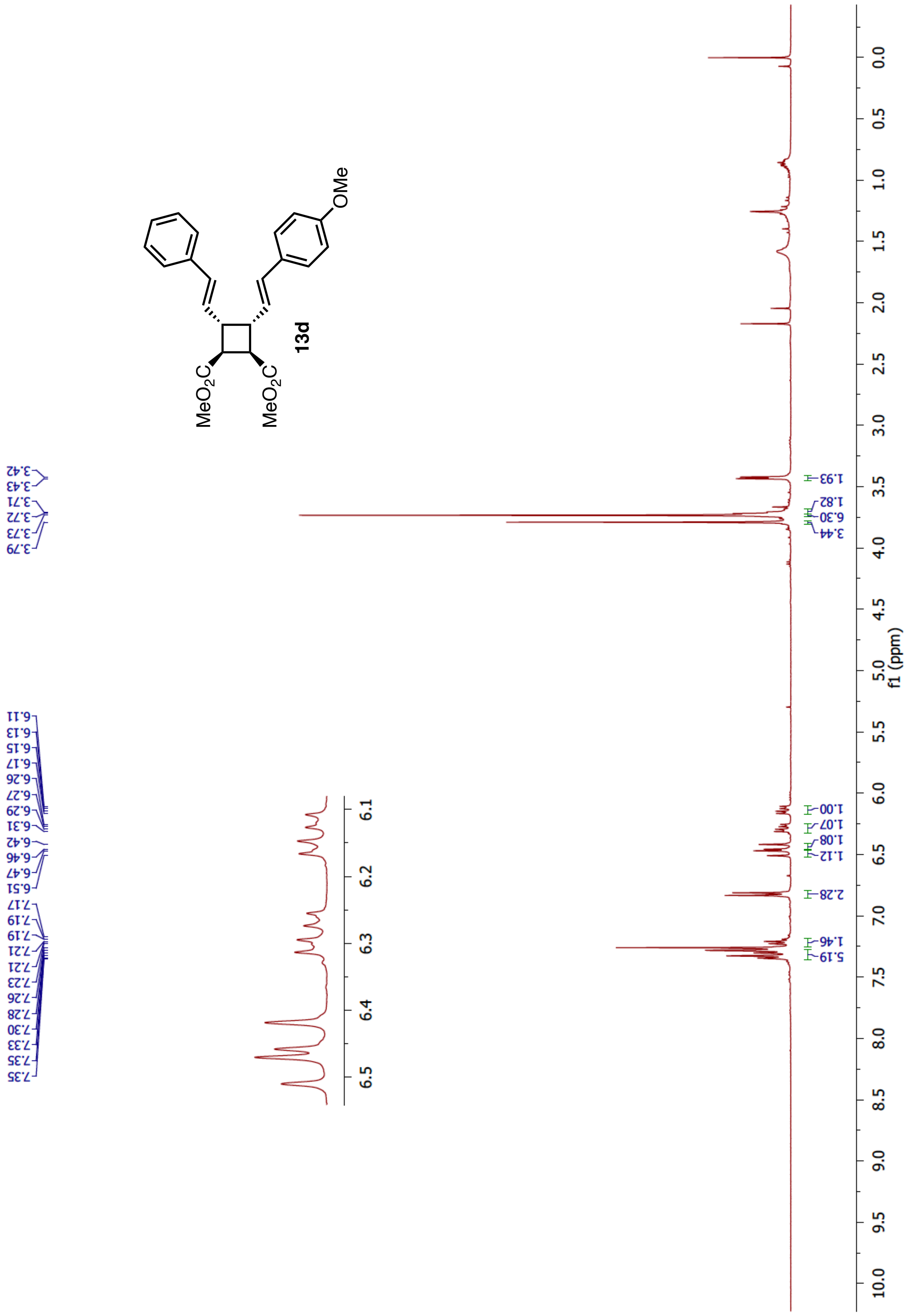

Figure S57. ^{1}H-NMR spectrum of **13d** in $CDCl_3$ (400 MHz).

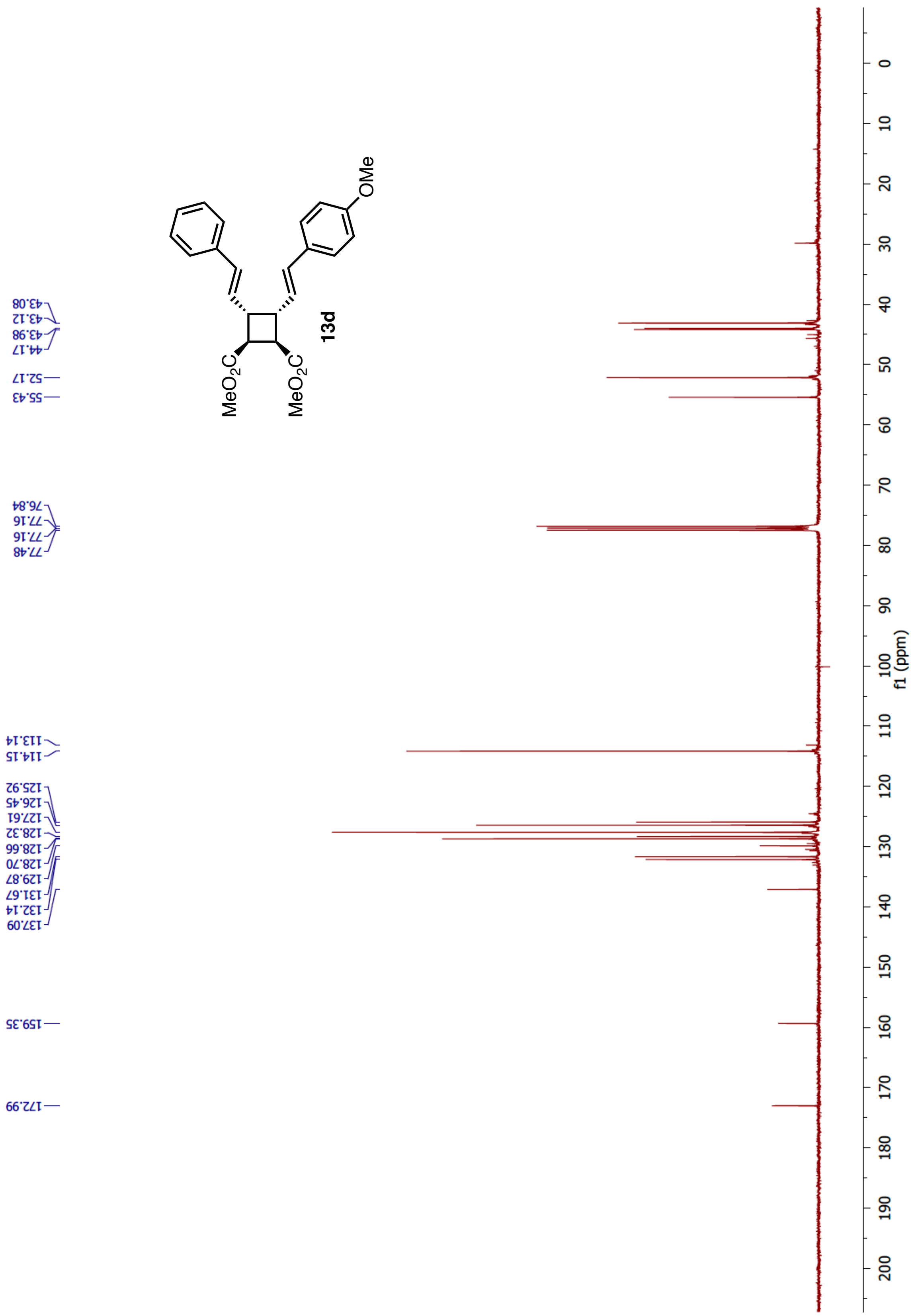

Figure S58. $^{13}C\{^{1}H\}$-NMR spectrum of **13d** in $CDCl_3$ (100 MHz).

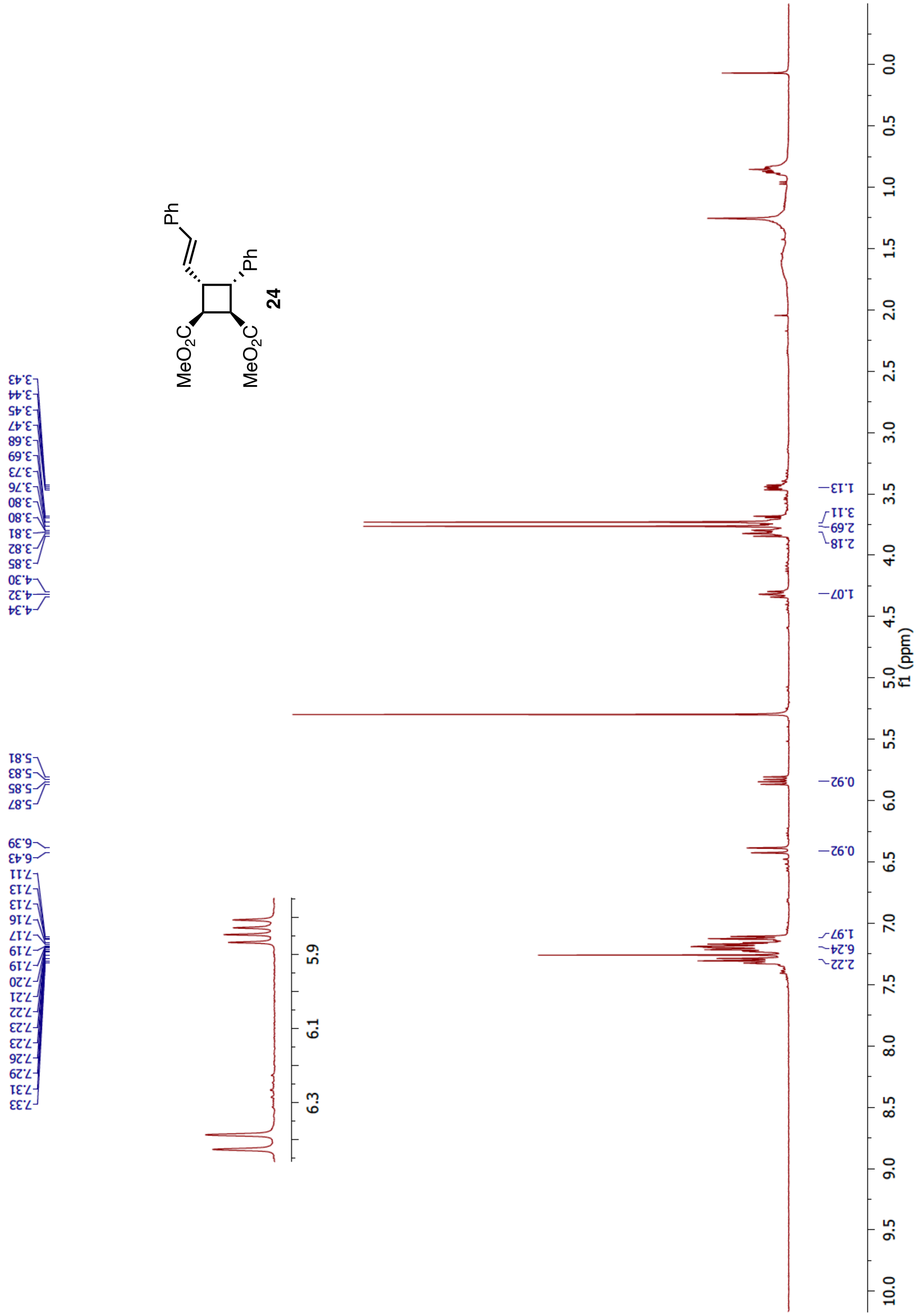

Figure S59. ^{1}H-NMR spectrum of **24** in $CDCl_3$ (400 MHz).

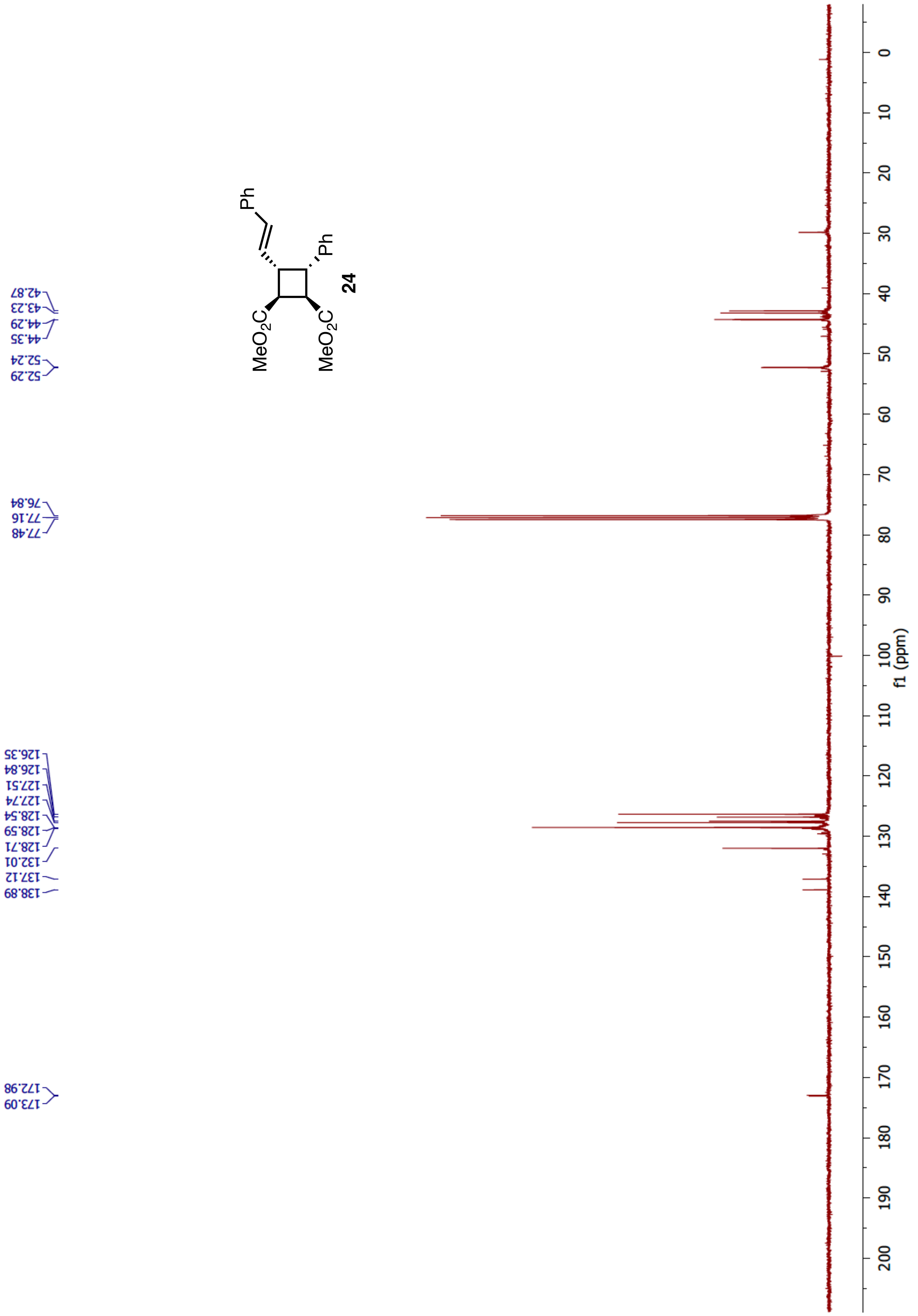

Figure S60. $^{13}C\{^{1}H\}$-NMR spectrum of **24** in $CDCl_3$ (100 MHz).

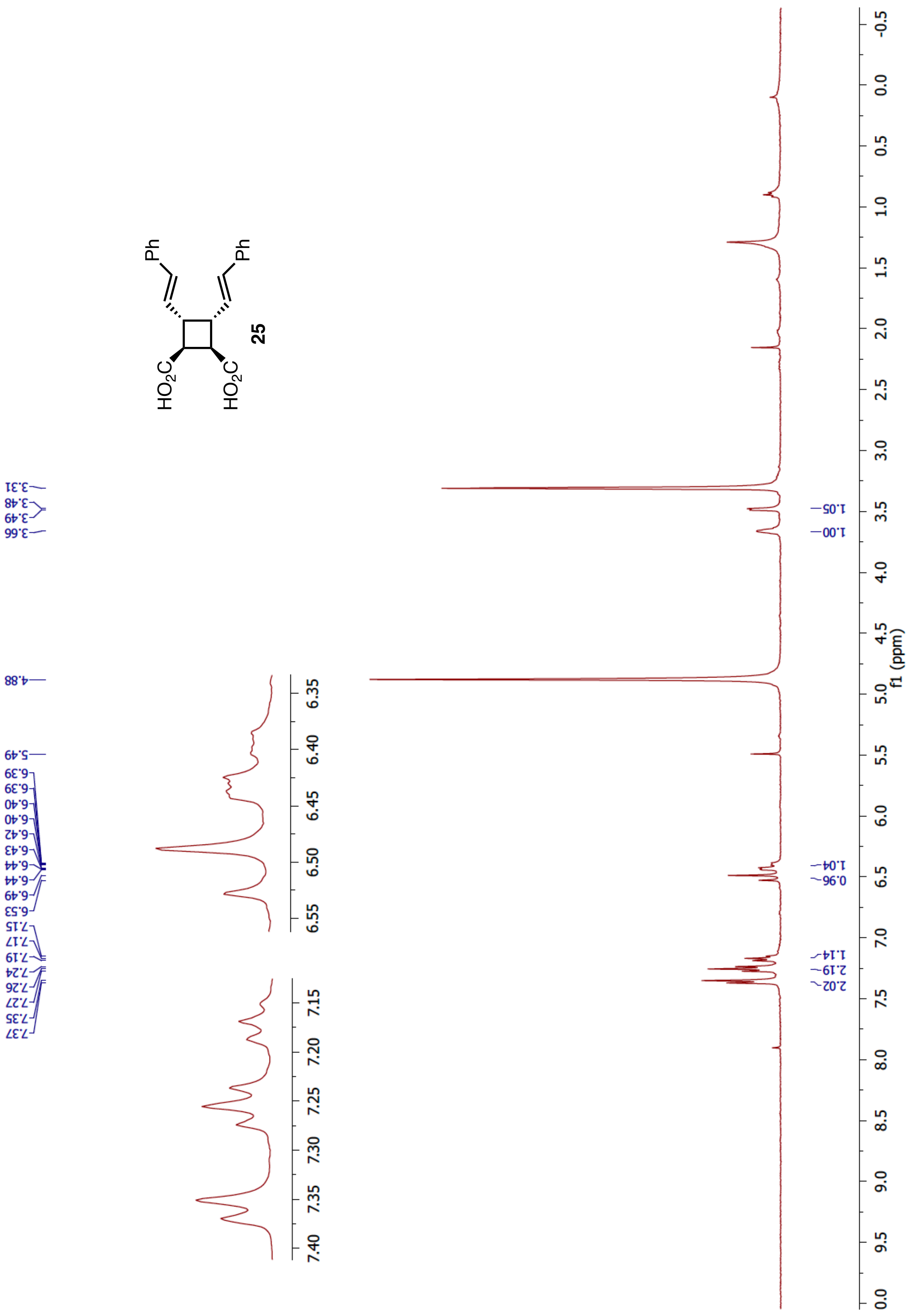

Figure S61. ^{1}H-NMR spectrum of **25** in CD_3OD (400 MHz).

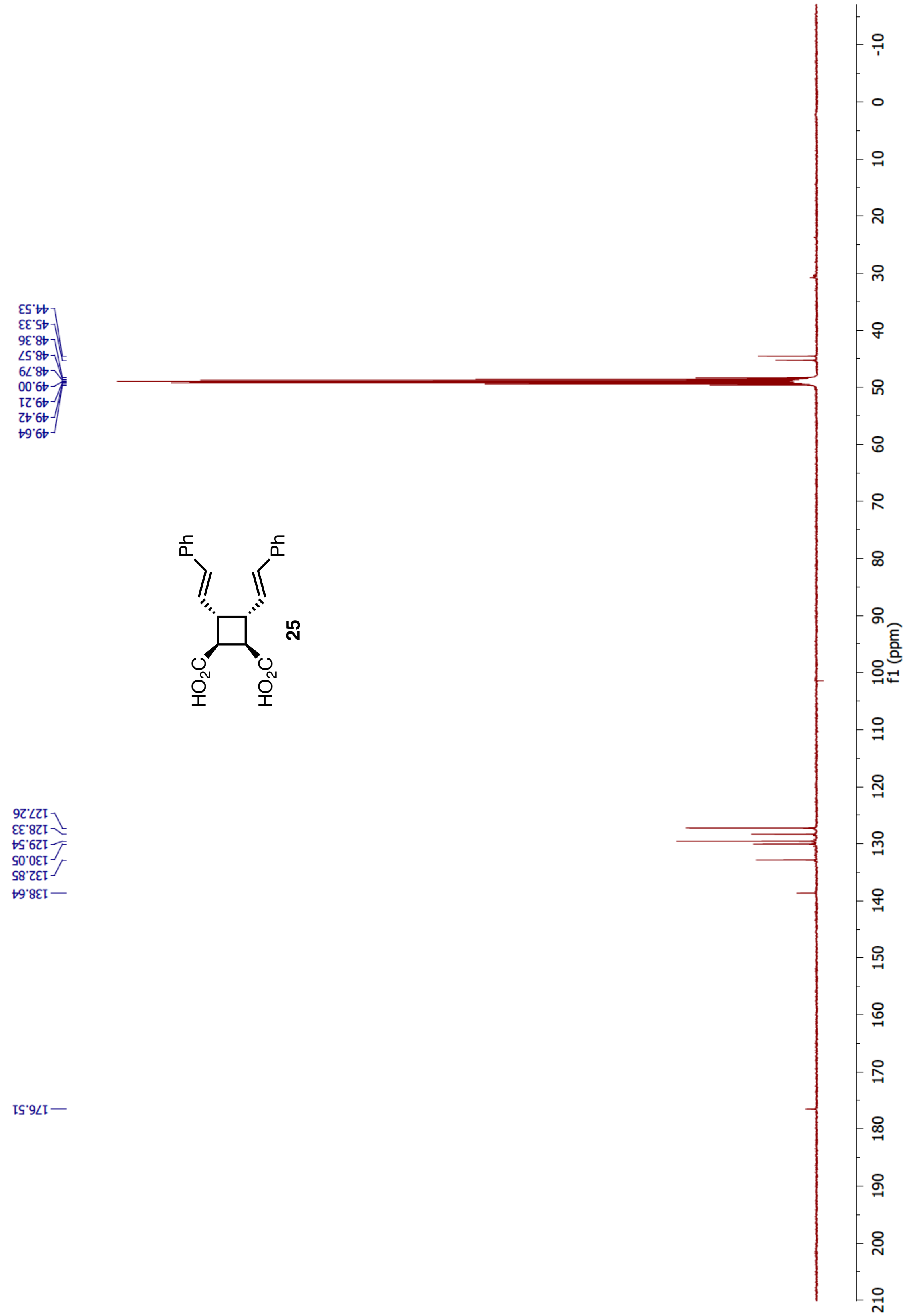

Figure S62. $^{13}C\{^{1}H\}$-NMR spectrum of **25** in CD_3OD (100 MHz).

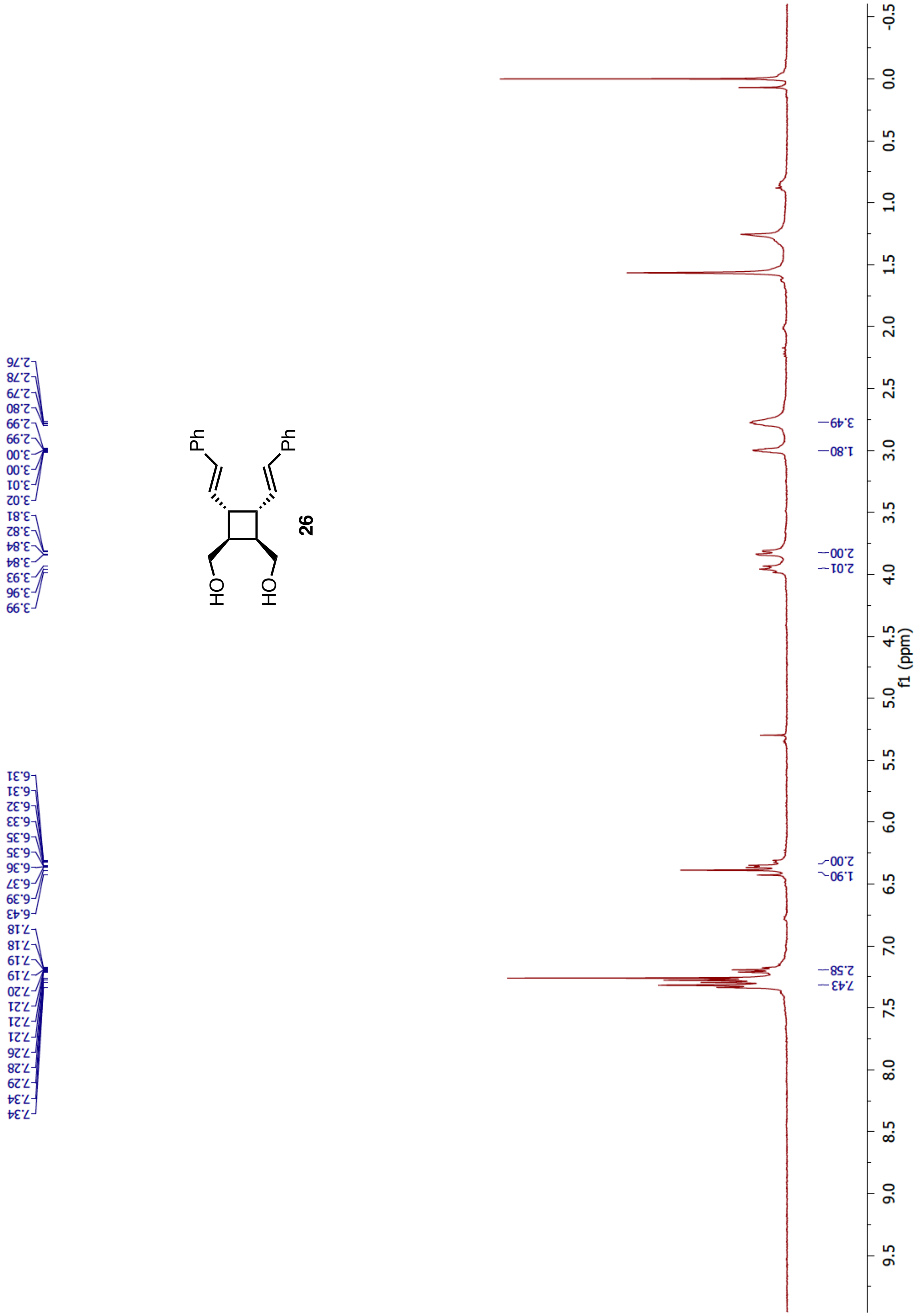

Figure S63. ^{1}H-NMR spectrum of **26** in $CDCl_3$ (400 MHz).

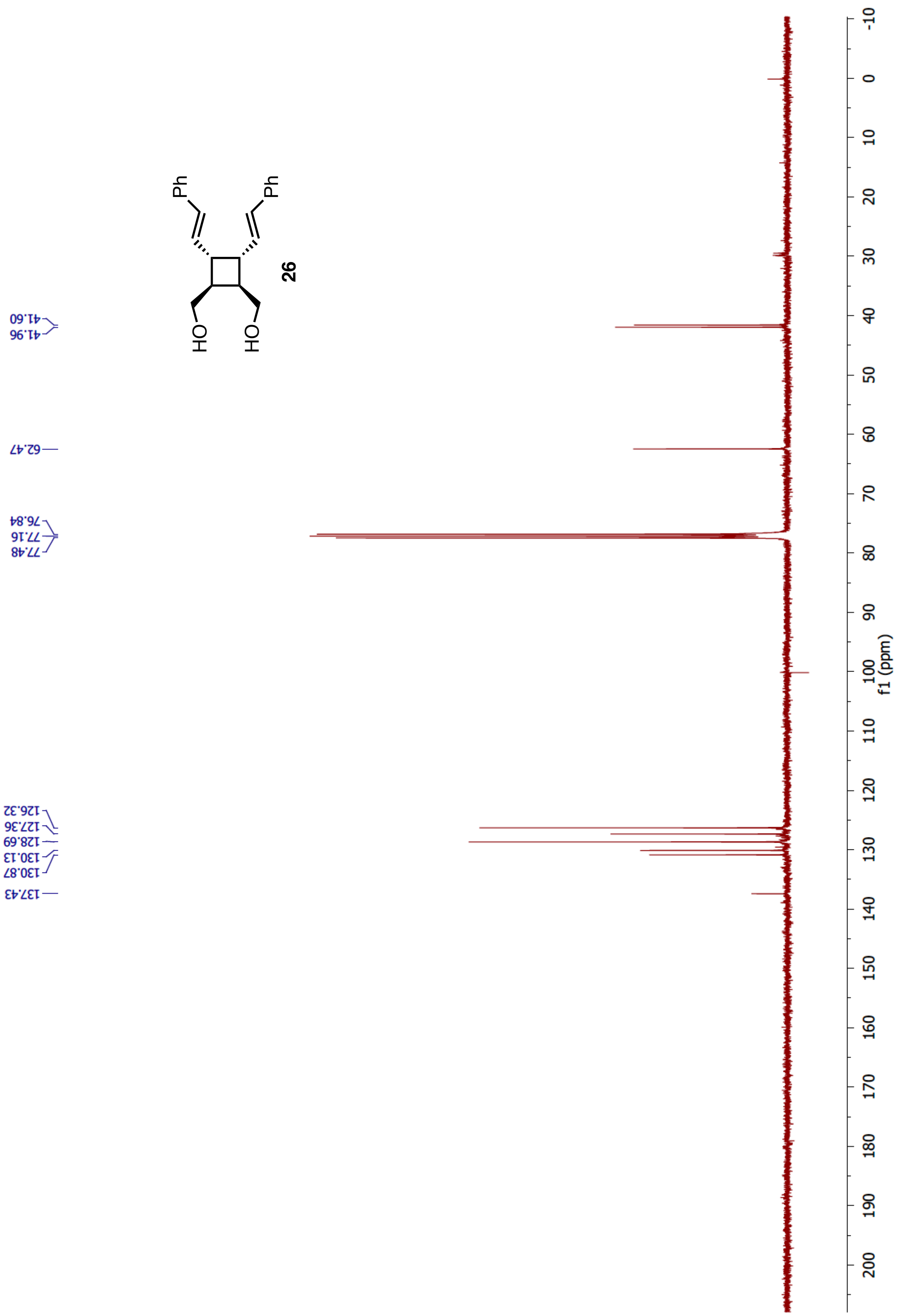

Figure S64. $^{13}C\{^{1}H\}$-NMR spectrum of **26** in $CDCl_3$ (100 MHz).
